# Potential Adverse Public Health Effects Afforded by the Ingestion of Dietary Lipid Oxidation Product Toxins: Significance of Fried Food Sources

**DOI:** 10.3390/nu12040974

**Published:** 2020-04-01

**Authors:** Martin Grootveld, Benita C. Percival, Justine Leenders, Philippe B. Wilson

**Affiliations:** Leicester School of Pharmacy, De Montfort University, The Gateway, Leicester LE1 9BH, UK; P11279990@my365.dmu.ac.uk (B.C.P.); justine.leenders@dmu.ac.uk (J.L.); philippe.wilson@dmu.ac.uk (P.B.W.)

**Keywords:** lipid oxidation products, lipid hydroperoxides, aldehyde toxins, frying oils, fried foods, cytogenicity/gentoxicity/mutagenicity, cancer, atherosclerosis, acrolein, cooking oil fumes, maximum human dietary intake (MHDI)

## Abstract

Exposure of polyunsaturated fatty acid (PUFA)-rich culinary oils (COs) to high temperature frying practices generates high concentrations of cytotoxic and genotoxic lipid oxidation products (LOPs) via oxygen-fueled, recycling peroxidative bursts. These toxins, including aldehydes and epoxy-fatty acids, readily penetrate into fried foods and hence are available for human consumption; therefore, they may pose substantial health hazards. Although previous reports have claimed health benefits offered by the use of PUFA-laden COs for frying purposes, these may be erroneous in view of their failure to consider the negating adverse public health threats presented by food-transferable LOPs therein. When absorbed from the gastrointestinal (GI) system into the systemic circulation, such LOPs may significantly contribute to enhanced risks of chronic non-communicable diseases (NCDs), e.g. , cancer, along with cardiovascular and neurological diseases. Herein, we provide a comprehensive rationale relating to the public health threats posed by the dietary ingestion of LOPs in fried foods. We begin with an introduction to sequential lipid peroxidation processes, describing the noxious effects of LOP toxins generated therefrom. We continue to discuss GI system interactions, the metabolism and biotransformation of primary lipid hydroperoxide LOPs and their secondary products, and the toxicological properties of these agents, prior to providing a narrative on chemically-reactive, secondary aldehydic LOPs available for human ingestion. In view of a range of previous studies focused on their deleterious health effects in animal and cellular model systems, some emphasis is placed on the physiological fate of the more prevalent and toxic α,β-unsaturated aldehydes. We conclude with a description of targeted nutritional and interventional strategies, whilst highlighting the urgent and unmet clinical need for nutritional and epidemiological trials probing relationships between the incidence of NCDs, and the frequency and estimated quantities of dietary LOP intake.

## 1. Introduction

An increasingly large proportion of the human population consuming Western World diets frequently ingest oxidised/peroxidised lipids, and the possibility that regular ingestion of such agents may be deleterious to human health has recently attracted a large amount of high-impacting research interest and focus [1,2,3,4,5]. 

**Figure 1:**
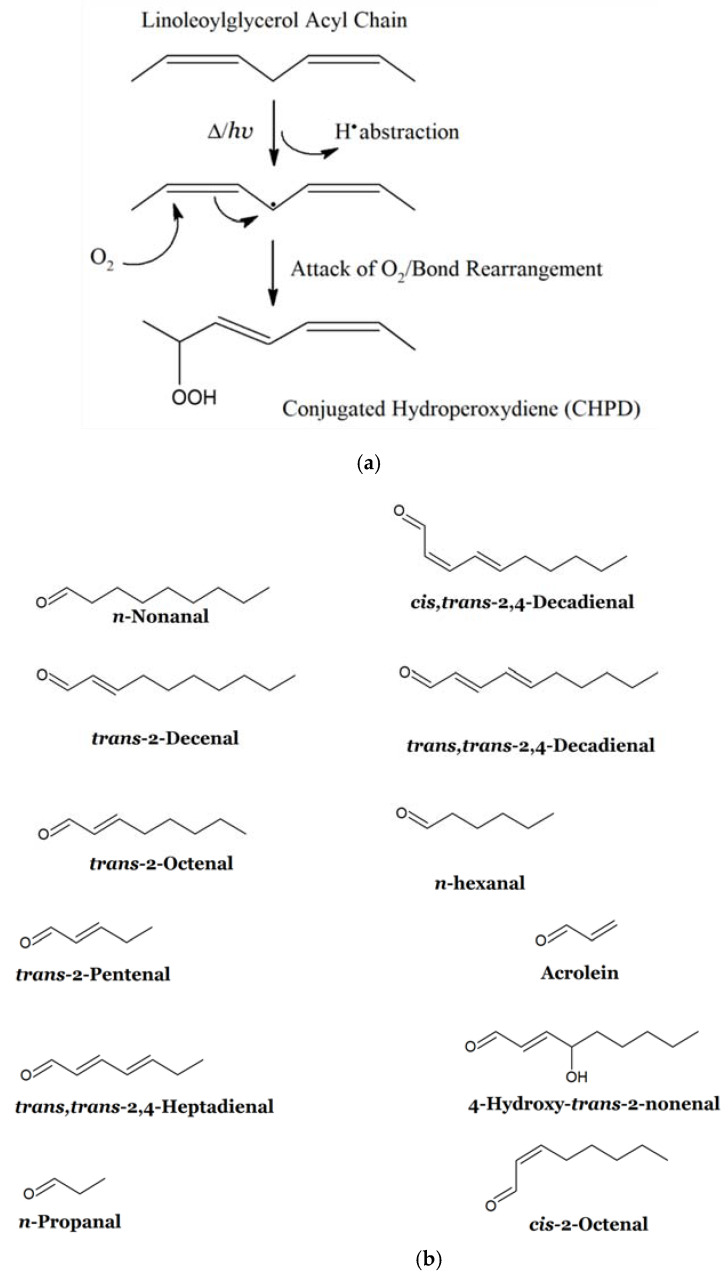
(**a**) Simplified reaction scheme for the peroxidation of a linoleic acid substrate molecule present in a culinary oil linoleoylglycerol species (H^●^ represents a hydrogen atom); the conjugated hydroperoxydiene (CHPD) species shown is one of the *cis,trans*-CHPD classification. (**b**) Molecular structures of aldehydes arising from the fragmentation of lipid hydroperoxides (HPMs and CHPDs). *n*-Nonanal and *trans*-2-decenal arise from the fragmentation of oleoylglycerol-derived HPMs; *n*-hexanal, *trans*-2-octenal and *trans,trans*-deca-2,4-dienal from the fragmentation of linolenoylglycerol-derived CHPDs; and propanal, acrolein, *trans*-2-pentenal and *trans,trans*-hepta-2,4-adienal from linolenoylglycerol-derived CHPD fragmentation. *cis,trans*-Deca-2,4-dienal may arise from the thermally-induced isomerism of its *trans,trans*-isomer [12].

Such lipid oxidation products (LOPs), which include cytotoxic and genotoxic aldehydes, along with their lipid hydroperoxide precursors, epoxy-fatty acids, and many other secondary or even tertiary LOPs [6,7], arise from the peroxidative deterioration of unsaturated fatty acids (UFAs), particularly polyunsaturated fatty acids (PUFAs), and are commonly encountered in UFA-rich culinary oils (COs), e.g. , refined, non-genetically-engineered natural corn, sunflower or soybean oils, when exposed to high temperature frying practices at *ca.* 180 °C, or when stored at ambient temperatures for prolonged durations [8,9,10,11,12] (Figure 1).

Aldehydes act as potent toxins since they are extremely chemically-reactive [1,3,7,13]. Indeed, they cause damage to critically important biomolecules such as DNA: since they are powerful electrophilic alkylating agents, the α,β-unsaturated classes of these aldehydes readily alkylate DNA base adducts, and this generally but not exclusively serves to explain their now established mutagenic, genotoxic and carcinogenic properties. Higher concentrations of this reactive aldehyde are effective in potently suppressing a wide range of cellular processes, which leads to indiscriminant cellular damage and ultimately apoptosis [13]. 

This Commentary paper focuses on the very wide range of potential public health threats presented by both primary LOPs (lipid hydroperoxides) and their secondary fragmentation products (aldehydes, etc.). Primarily, Section 2 provides an extensive comprehensive review of all possible dietary sources of LOPs, and includes subsections focused on estimates of individual dietary aldehyde intake, most especially the molecular nature and contents of those detectable in fried foods, along with estimated risk assessments of their consumption by humans. Section 3 delineates the GI system interactions, in vivo absorption, metabolism and biotransformation, toxicological properties and potential adverse health effects of these agents respectively. Data available has provided powerful evidence that only secondary LOPs (particularly aldehydes and epoxy-acids), and not their primary hydroperoxide precursors, are transferred to foods during high-temperature frying practices, and that these toxins have sufficient longevity therein [12], a factor which renders them freely available for ingestion by human populations. Unequivocally, such considerations are also of major concern for home-cooking domestic consumers, and perhaps more importantly restaurant workers, who are frequently exposed to aldehyde-containing emissions arising from cooking oils during such frying episodes, or alternative high-temperature cooking practices such as those involving Chinese-style woks [14]. Section 3 begins with a full evaluation of the biomolecular pathways and probable physiological fates of aldehydic LOPs and their conjugated hydroperoxydiene (CHPD) precursors; potential associations of the fractional contents of different classes of aldehydes in fried foods and those of human blood plasma are also explored for the first time. Since many previous investigations have focused on the potential roles of dietary LOPs and their fried food sources as major risk factors for the induction and development of atherosclerosis and its cardiovascular disease sequelae, and cancer, a review of these involvements and their adverse health implications are provided in Section 4 and Section 5, respectively (acrolein, crotonaldehyde and *trans,trans*-2,4-decadienal as inhaled or ingested carcinogens represent special cases for consideration). Subsequently, Section 6 discusses potential mechanisms available for the toxicities and associated adverse health effects of dietary aldehydes, with a critical consideration of the concentrations of these agents available in the GI system, the systemic circulation and elsewhere in vivo. Finally, Section 7 explores targeted nutrition and potential interventional strategies for diminishing the amounts of dietary LOPs available in the human diet, and which hopefully will provide effective barriers to health risks posed by their ingestion; alternative ‘anti-aldehyde’ prophylactic or therapeutic strategies are also discussed. This section also considers the performance of further, more intense research investigations to establish, optimize and validate maximum human daily intake (MHDI) values for a full range of such dietary aldehydes, rather than relying on the very limited data currently available. Throughout the text, reference to a series of examples of the multicomponent analysis of secondary aldehydic LOPs in COs and foods (fried or otherwise) is made in the Figures provided. The urgent requirement for future clinical feeding trials or epidemiological investigations focused on explorations of relationships between the incidence and/or severity of chronic non-communicable human diseases (NCDs), and the frequency and levels of dietary LOP intake, is stressed in the Conclusions section. In view of the focus of this Commentary on lipid hydroperoxides and their aldehydic chain-cleavage products, the in vivo absorption and toxicities of dietary epoxy-fatty acids (FAs) are summarized in Section S1 of the Supplementary Materials section. 

## 2. Systematic Review of Major Points

### 2.1. Lipid Peroxidation Process: Mechanistic Considerations and Relative Susceptibilities of Acylglycerol FAs

The peroxidation of UFAs during high-temperature frying practices (*ca.* 180 °C) represents a complex oxidative deterioration process involving chemically-reactive free radical species (i.e., reactive chemical species with one or more unpaired electrons), and similarly-reactive singlet oxygen (^1^O_2_). For PUFAs, primarily this process involves the heat- and/or light-induced loss of a hydrogen atom (H^●^) from relatively weak *bis*-allylic-CH_2_- function carbon-hydrogen (C-H) bonds to generate a resonance-stabilised pentadienyl carbon-centred radical (L^●^), which then reacts with atmospheric dioxygen (O_2_) to form a reactive peroxyl radical (LOO^●^) (Figure 1a). Structurally, these PUFA-specific *bis*-allylic-CH_2_- functions may be viewed as being ‘sandwiched’ between two strongly electron-withdrawing carbon-carbon double bonds (>C=C<), and this explains the weakness of their C-H bonds, which facilitates the abstraction of H^●^ therefrom. Once formed, LOO^●^ radicals can then continue to react with another, adjacent PUFA molecule to generate an additional L^●^ radical, the unpaired oxygen-centred electron of the peroxyl radical being converted to a more stable lipid hydroperoxide (LOOH) species, which for PUFAs are known as conjugated hydroperoxydienes (CHPDs). Hence, this process is known as an autocatalytic, self-propagating chain reaction, which unless terminated by the donation of another H^●^ from a suitable lipid-soluble chain-breaking antioxidant (e.g. , phenolic agents such as alpha-tocopherol (α-TOH)), will continue relentlessly until all PUFAs have been consumed. In view of its autocatalytic nature, plots of aldehydic LOP concentrations generated against time usually appear as S-shaped curves, i.e., as sigmoidal relationships [3,12]. 

Once formed, CHPDs fragment to a wide range of degradation products (secondary LOPs), particularly at high frying temperatures, and these include extremely toxic aldehydes in particular [1,7,8]. Further CHPD deterioration products include alcohols, ketones, oxo-acids, alkanes and alkenes [1,7,15,16,17], in addition to epoxy-fatty acids such as 9,10-epoxy-12-octadecenoate, which is also known as leukotoxin [18].

However, monounsaturated fatty acids (MUFAs), which produce corresponding hydroperoxymonoenes (HPMs) in the same manner, are much more resistant to peroxidation than PUFAs since they only have *mono*-allylic-CH_2_- functions, with stronger C-H bonds than those of the *bis*-allylic-CH_2_- fucntions in PUFAs. Hence, MUFAs give rise to lower or much lower levels of both primary and secondary LOPs when heated in this manner, with a much less broader range of secondary aldehydic LOP classifications than those derived from PUFAs, and generally only after prolonged exposures at standard frying temperatures [12]. For example, thermal stressing of MUFA-rich olive oil generates much lower levels of aldehydes than those observed with PUFA-rich sunflower or corn oils, and are predominantly limited to only *trans*-2-alkenals and longer-chain *n*-alkanals. As expected, saturated fatty acids (SFAs) are virtually completely resistant to peroxidative damage, even at high frying temperatures. Therefore, the order of toxic LOP production in culinary oils is PUFAs >> MUFAs >>>>> SFAs, and hence PUFA-rich culinary oils represent the riskiest choice for use as frying media, especially when exposed to repeated frying episodes [2,3,12]. Indeed, the relative oxidative susceptibilities of these lipid classes are 1:100:1,200:2,500 for 18-carbon chain length fatty acids containing 0:1:2:3 >C=C< functions respectively [8]. Moreover, the rate of fragmentation of CHPDs or HPMs to the above series of smaller molecular LOPs also increases with increasing FA unsaturation status, i.e., it is in the order linolenoyl- > linoleoyl- >>> oleoylglycerols [9].

### 2.2. Dietary Sources of LOPs

An extensive review of the dietary availability of LOPs is provided in Section S2 of the Supplementary Materials. These comprise outlines of the adverse generation and analysis of LOPs in red meat, chicken and poultry (Section S2.1); fish products (Section S2.2); dairy products (Section S2.3); grain products (Section S2.4); fruits and vegetables (Section S2.5); and alcoholic beverages (Section S2.6). Moreover, a full outline of the use of aldehydes as food flavouring agents is presented in Section S2.7, and their deleterious generation in thermoplastic food packaging materials is summarised in Section S2.8.

A review of estimations of dietary aldehyde intake by humans, and the essential considerations required for such criteria, is provided in this section. Used and reused culinary frying oils, and more especially foods which have been fried therein and therefore have uptaken variable levels of such LOP-containing media, e.g. , potato chips, chicken portions and beef patties, etc., serve as rich and very important dietary sources of these toxins (especially aldehydes) in view of their frequent human consumption in Western diets. Therefore, this area is covered in full detail below (Section 2.3). Section 2.4 outlines the exposure of human populations to fried food sources of dietary LOPs, along with rational estimates of their ingestions (including sub-sections focused on acrolein and 4-hydroxy*-trans*-2-nonenal/4-hydroxy-trans-2-hexenal (HNE/HHE)). Moreover, Section 2.5 provides information on the risk assessment of environmental aldehydes, with special reference to the computation of margin of exposure (MOE) values. 

Wang et al. [19] conducted an extensive review of the availability of human exposure to environmental aldehydes from various water, food, tobacco cigarette and ambient air sources (Table 1). Of the foods listed, dietary acrolein intake from vegetables, donuts, cheese and red wine are very high, the latter providing as much as 3.8 g/kg of this aldehyde alone! Similarly, vinegar and coffee are very rich sources of acetaldehyde (1.06 g/kg) and furfural (255 mg/kg) respectively, whereas anise contains a staggeringly high content of anisaldehyde (25 g/kg).

However, in Table 1, only estimated acrolein concentration values were provided for heated lard and sunflower oil, these values being 109 and 163 mg/L repectively, and therefore our determined concentrations of all other alkenals (predominantly *trans*-2-alkenals such as *trans*-2-octenal) and the range of all other aldehyde classifications (including relatively predominant *trans,trans*-alka-2,4-dienals, along with lower levels of substituted alkenals and *n*-alkanals) will undoubtedly provide an additional and significant contribution to these environmental aldehyde levels, most notably for consumers with a high incidence of fried food intake. Indeed, the only mention of fried food sources in Table 1 is codfish fillets, which contained an acrolein content of 100 mg/kg. Notwithstanding, heated butter was noted to provide low levels of 2-pentenal and 2,4-nonadienal, but this was the only other peroxidised UFA source of aldehydes mentioned. 

Wang et al. [19] also provided estimates of the mean daily consumption of acrolein form a range of food sources, and these were cheese (40 µg); donuts (380 µg); codfish fillets (10 µg); wine (1,520 µg); fruits (15 µg); vegetables (250 µg); potatoes (150 µg); and edible cooking oils (10 µg). However, the latter value will be critically dependent on frying oil sources (those with relatively high levels of ω-3 fatty acids yielding higher levels of this aldehyde), and also their use and reuse status. From these 8 classes of foods, the average human daily consumption of acrolein alone was estimated to be 2.35 mg. However, since at that time it was known that this LOP was detectable in 35 food classes, the estimated maximal daily food consumption level in [19] was 5.0 mg/day. Moreover, exposure from the smoking of tobacco cigarette products (50–100 µg per cigarette) was estimated, which is equivalent to an additional 1.0–2.0 mg for a human smoking 20 cigarettes per day.

Of the above unsaturated aldehyde estimate, that for the maximal daily human exposure of acrolein from a combination of food and water sources alone was found to be 0.1 mg/kg BW, along with an equivalent quantity estimated from tobacco smoking, if appropriate [19].

Consideration of only the estimated non-smoking human contribution, this value in itself is already 200-fold greater than the ADI value of 0.5 μg/kg BW specified for this aldehyde by the Australian Government Department of Health (AGDH) [20], which is clearly a major cause for concern. Dietary sources and estimated dietary intakes of acetaldehyde and formaldehyde, both of which are also generated in the lipid peroxidation process (from the degradation of secondary aldehydic LOPs, e.g. , MDA for the latter [12]), are provided in Section S3 of the Supplementary Materials. Moreover, comparative evaluations of the dietary availability for ingestion of aldehydic LOPS with those of the process toxins acrylamide, monochloropropanediol (MCPD) adduct toxins, and *trans*-FAs are discussed in Section S4 (Supplementary Materials).

### 2.3. Fried Food Sources of LOPs

Very high concentrations of LOPs, particularly secondary aldehydic ones, are generated during such processes in view of the autocatalytic, self-propagating nature of this singlet oxygen (^1^O_2_)-catalysed and lipid peroxyl radical (LOO)-mediated peroxidation process [14,15,16,17]. Indeed, the total α,β-unsaturated aldehyde concentration measured in PUFA-rich COs such as sunflower oil thermally-stressed for a period of 90 min. according to laboratory-simulated shallow frying episodes (LSSFEs) can reach values as high as 50 mmol/kg [12]. These unsaturated aldehydes are more toxic than the saturated classes of these compounds which are also generated, and adversely represent 70–75% of the total aldehyde remaining in COs heated in this manner. Notwithstanding, the very high total levels of aldehydes often found in used UFA-rich frying oils only represent those retained after the loss of substantial amounts of them through volatilisation processes, so that they also represent components of very harmful cooking oil fumes. Health hazards arising from the human inhalation of such aldehyde-laden fumes in poorly-ventilated kitchen areas are also discussed herein.

**Figure 2:**
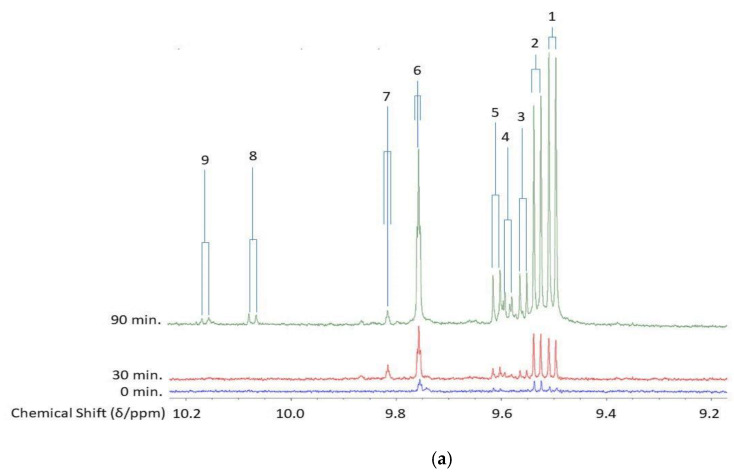

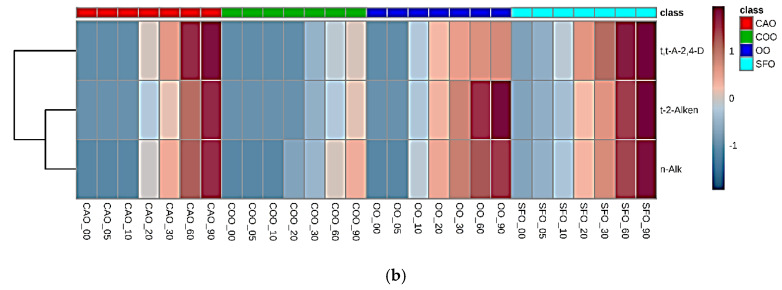
(**a**) Expanded aldehydic-CHO proton (9.20–10.20 ppm) regions of 600 MHz ^1^H-NMR spectra of corn oil exposed to laboratory-simulated frying episodes at 180 °C for periods of 0 (blue), 30 (red) and 90 min. (green). Typical spectra are shown. Abbreviations: -CHO function resonances of 1, *trans*-2-alkenals; 2, *trans,trans-*2,4-alkadienals; 3, 4,5-epoxy-*trans*-2-alkenals; 4, combined 4-hydroxy and 4-hydroperoxy-*trans*-2-alkenals; 5, *cis,trans*-2,4-alkadienals; 6, *n*-alkanals; 7, low-molecular-mass short-chain *n*-alkanals, particularly propanal and *n*-butanal from the peroxidation of linolenoylglycerols; 8, *cis*-2-alkenals, potentially arising from the thermally-induced isomerism of *trans*-2-alkenals; 9, unassigned aldehyde doublet resonance. All resonances visible are doublets, with the exception of signals 6 and 7, which are triplets (*J* = 1.73 and 1.74 Hz respectively). Samples were prepared for ^1^H NMR analysis by the method described in [11], and spectra were acquired on a JEOL-ECZR600 NMR spectrometer (De Montfort University facility, Leicester, UK) operating at a frequency of 600.17 MHz. (**b**) Heatmap profile showing the time-dependent generation of the three major secondary aldehydic LOPs, i.e., *trans*-2-alkenals (t-2-Alken), *trans*,*trans*-2,4-alkadienals (t,t-A-2,4-D) and *n*-alkanals (n-Alk) in canola (CAO), coconut (COO), extra-virgin olive (OO) and sunflower (SFO) oils exposed to LSSFEs for periods of 0, 5, 10, 20, 30 60 and 90 min. (ordinate axis codes 00, 05, 10, 20, 30, 60 and 90 respectively). Generalised log- (glog-) transformed aldehyde concentrations (mmol/mol. FA) are shown on the right-hand side abscissa axis. Deep blue and red colourations depict extremes of low and high concentrations respectively. The left-hand abscissa axis shows agglomerative hierarchal clustering of these 3 aldehyde classes, which demonstrate that *trans*,*trans*-alka-2,4-dienals, which are generated only from PUFA peroxidation, have some independence (orthogonality) from a combination of *trans*-2-alkenals and *n*-alkanals, which arise from the fragmentation of both MUFA and PUFA hydroperoxide sources. Manufacturer-specified SFA, MUFA and PUFA contents of these oils were 7.5, 63.7 and 28.8% for canola oil; 90.1, 8.1 and 1.8% (w/w) respectively for coconut oil; 13.0, 77.4 and 9.4% for extra virgin olive oil; and 10.3, 29.3 and 60.4% (w/w) for sunflower oil. For canola oil, 9.8% of the 28.8% (w/w) PUFA content was linolenic acid (as linolenoylglycerols).

**Figure 3:**
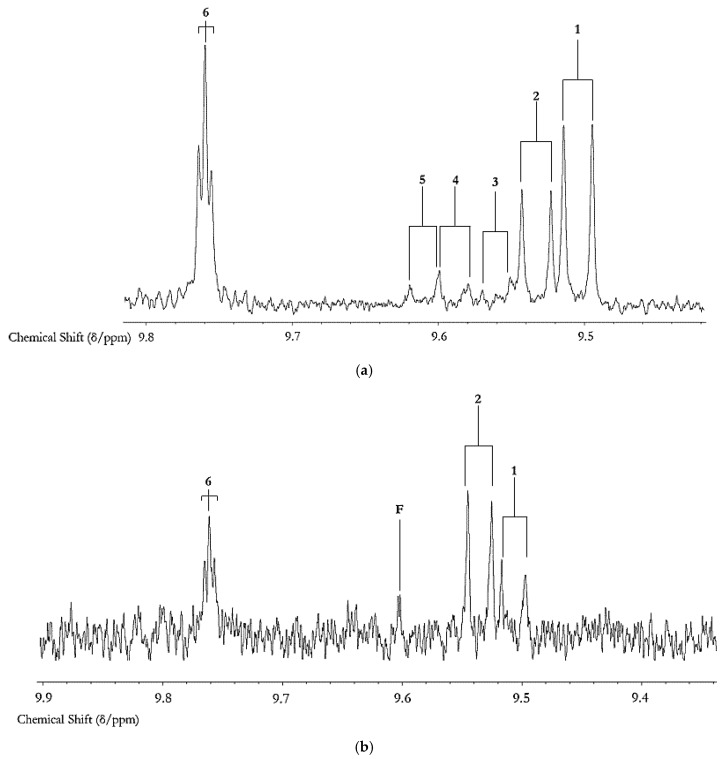
^1^H-NMR Analysis of Aldehydic LOPs in C^2^HCl_3_ Extracts of Fast-Food Restaurant Fried Food Samples. (**a**) and (**b**), Expanded aldehydic-CHO proton (9.40–9.90 ppm) regions of the 400 MHz ^1^H NMR spectra of C^2^HCl_3_ extracts of fried potato chip and chicken (batter portion) servings purchased from fast-food restaurants, which contain *trans*-2-alkenal, *trans,trans*-2,4-alkadienal, 4,5-epoxy-*trans*-2-alkenal, combined 4-hydroxy-/4-hydroperoxy-*trans*-2-alkenal, *cis,trans*-2,4-alkadienal and *n*-alkanal aldehydic LOP resonances in (**a**), and *trans*-2-alkenal, *trans,trans*-2,4-alkadienal and *n*-alkanal resonances in (**b**). Typical spectra are shown. Typically, no aldehydic LOPs were ^1^H NMR-detectable in the corresponding meat portion of the fried chicken sample corresponding to the batter extract spectrum shown in (**b**). Samples were extracted and prepared for ^1^H NMR analysis by the method described in [11], and spectra were acquired on a 400 MHz Bruker Avance NMR spectrometer equipped with a QNP probe, and operating at 399.93 MHz (De Montfort University facility, Leicester, UK). Abbreviations: as Figure 1, with F representing formaldehyde in (**b**).

Our research group’s extensive historic investigations of the oxidative deterioration of PUFA-rich COs during standard frying practices, and their availability for uptake into fried foods such as potato chips and crisp snacks, etc. available for human ingestion, have been accessible to the scientific community since 1994 [6]. However, since that time, major advances in the development of analytical/bioanalytical techniques for the investigation of patterns and concentrations of LOPs in both food, and biofluid and solid biopsy samples, have been made. Indeed, earlier problems and complications experienced with the high-resolution NMR analysis of such samples, including sensitivity issues, have now been largely overcome via the application of newly-developed pulse sequences, for example. The aldehydic-CHO function regions of the 600 MHz ^1^H-NMR spectra of a commercially-available corn oil product exposed to laboratory-simulated shallow frying episodes (LSSFEs) for periods of 0, 30 and 90 min. are shown in Figure 2, together with a heatmap diagram displaying the critical dependence of the concentrations of three major aldehydic LOPs generated in four different COs (of variable SFA, MUFA and PUFA contents) on increasing LSSFE time-points. These data clearly demonstrate that the heating period-dependent levels of CO aldehydes generated are high in PUFA-rich oils (corn and sunflower oils), intermediate in MUFA-rich ones (canola oil), and much lower in SFA-laden coconut oil. More recently, we have confirmed passage of these secondary LOP toxins from thermally-stressed frying oils into foods fried therein (Figure 3), and have estimated their contents, which are consistently and considerably greater than those of acrylamide and monochloro-propanediol (MCPD) adducts [3,14] (Section S4). Indeed, samples of repeatedly-used frying oils collected from domestic kitchens, fast-food retail outlets and restaurants have confirmed the generation of aldehydic and further LOP toxins at high concentrations during ‘on-site’ frying practices [3,14]. Our original research studies have been repeated, replicated, and further exemplified by many research laboratories globally, e.g. , [9]. Encouragingly, it now appears that these highly important public health concerns are appreciated and respected by food science, nutrition and associated clinical researchers. 

### 2.4. Exposure of Human Populations to Fried Food Sources of Dietary Aldehydes: Rational Estimates of Their Dietary Ingestion from These Sources

Unfortunately, global governmental health recommendations for the maximum acceptable human daily intakes (MHDIs) of aldehydes (i.e., those which are considered to be an acceptable intake that may be ingested daily throughout an entire lifetime without these agents presenting any appreciable risk to human health) are either extremely limited or completely unavailable.

However, for acrolein (which is generated from the peroxidation of linolenoylyglycerols, or alternatively from direct oxidation of glycerol liberated from triacylglycerol backbones via hydrolysis reactions), the AGDH specified that this value was only 0.5 µg per kg of body weight, i.e., a total of only 35 μg for a mean human body weight of 70 kg [20]. Therefore, the observation that much higher contents of *trans*-2-alkenals, *trans,trans-2,4-alkadienals* and *n*-alkanals than this limit are present in fried potato chip servings purchased from a range of fast food ‘take-away/take-out’ restaurants [12] (Figure 3), indicates a critically-important public health concern.

Using the assumption that all the *trans*-2-alkenals generated in the frying oils employed by these outlets is *trans*-2-octenal (the predominant homologue of this aldehyde classification derived from the fragmentation of linoleoylglycerol hydroperoxides), its estimated content in what is described as a ‘large’ 154 g portion of this frequently-consumed fried food is 2.4 mg, a value which is *ca.* 70-fold larger than that of this acceptable daily human intake limit for its lower homologue acrolein (which corresponds to *ca.* 30-fold greater for its acrolein mass-equivalent figure of 1.04 mg). Parallel estimates for the most predominant *trans,trans*-2,4-alkadienal and *n*-alkanal (*n*-hexanal) agents produced from such linoleoylglycerol peroxidation sources were 3.8 and 1.9 mg (acrolein mass-equivalent values of 1.4 and 1.1 mg), respectively, within such a 154 g potato chip portion, and therefore the total aldehyde content of this typical fast-food source, or at least for that fried in a hypothetical vegetable-derived frying oil containing 100% (w/w) linoleoylglycerols, is (2.4 + 3.8 + 1.9) mg = 8.1 mg, with acrolein mass-adjusted values of (1.0 + 1.4 + 1.1) mg = 3.5 mg, of which *ca.* 70–75% (w/w) are the more toxic α,β-unsaturated classes. However, it should be noted that the value computed here is estimated from the consumption of a single staple fried food serving, and also that the above aldehydes are only three possible, albeit three of the most prevalent, classes of aldehydic LOPs detectable, out of a total of 10 or more of these generated in UFA-rich culinary vegetable oils during or following standard frying practices [3,14]. 

Similarly, assuming that all aldehydes are the most prevalent ones arising from the fragmentation of oleoylglycerol hydroperoxide (HPM) precursors, estimated potato chip portion contents of *trans*-2-alkenal and *n*-alkanal toxins generated are 2.9 and 2.8 mg respectively (*trans,trans-*2,4-alkadienals only arise from the fragmentation of PUFA-derived hydroperoxides) [12]. However, since oleoylglycerol peroxidation reaction rates are much slower than those of PUFAs, as are the rates of fragmentation of their HPMs to aldehydes and further products (i.e., it has a substantially lower peroxidative susceptibility index (PSI) value [1,16]), much lower levels of LOPs are therefore generated from such sources, and this explains why MUFA-rich oils such as olive oil are relatively highly resistant to thermally-induced oxidation during standard frying episodes. Indeed, peroxidative lag-phases for MUFA-rich cooking oils are much longer than those observed with oils which contain high PUFA contents, e.g. , sunflower or corn oils (Figure 2b). Several minor (lower content level) classes of cytotoxic/genotoxic aldehydes are also detectable in fried potato chip samples (e.g., *cis,trans*-2,4-alkadienals and formaldehyde), and this also adds significantly to the dietary aldehydic LOP load. 

Importantly, the above estimates pertain to only one 154 g-sized fried potato chip single meal serving, and those of 300 g, or more are also quite common in the Western diet. 

Recently, Grootveld et al. [3,14] demonstrated that, in addition to the unsaturation status of frying oils (reflected by their PSI values, which soar with increasing PUFA content), along with a range of other factors such as frying methods (i.e., deep *vs.* shallow frying practices), frying temperatures and durations, for example, the uptake of aldehydic LOP-containing culinary frying oils (monitored as total lipids through high-resolution ^1^H-NMR analysis) was a critical determinant of the aldehyde contents of fried potato chip products. However, the relative molecular content ratios of *trans*-2-alkenals, *trans,trans-*2,4-alkadienals and *n*-alkanals of these products did not reflect those present in their frying oil sources (which were also analyzed using ^1^H-NMR analysis in a C^2^HCl_3_ medium), with higher than expected *n*-alkanal contents. These results are best rationalized in terms of the higher level of reactivities of the α,β-unsaturated aldehyde classes with potato proteins, amino acids and further aldehyde-consuming biomolecules, over those of the saturated ones, or more specifically, the ability of these classes of aldehydes to engage in Michael addition reactions, unlike their saturated counterparts.

Interestingly, no further updates to the above AGDH ADI value of 0.5 µg per kg of BW, for acrolein were made in their updated Edition 1/2017 (current as of 31 March 2017). Neither CHPDs, nor HPMs, were detectable in these C^2^HCl_3_ fried food product sample extracts, and this observation is presumably ascribable to their reactions with potato chip electron donors such as the amino acid L-cysteine and/or other thiol function-containing biomolecules therein to form less toxic conjugated hydroxydiene species, and/or their catalytic deterioration to further aldehydes and other secondary LOP fragmentation products [16]. Indeed, formic acid, a product arising from the degradation of MDA, was also detectable in these extracts. Therefore, from this investigation, it appears that commonly-fried food products act as poor sources of CHPDs and HPMs, but nevertheless are rich in their aldehydic degradation products. 

Of further pertinence, shallow-frying episodes generate much higher levels of frying oil LOPs than deep-frying ones in view of the greater surface area of the oil in the former case, i.e., its much greater exposure to atmospheric O_2_ required for the lipid peroxidation process [1,7,14] (Figure 1). For deep-frying practices, LOPs are only formed on the O_2_-exposed and -richer surface environment of the oils employed for this purpose, and subject to a sufficient level of oil admixing/homogenisation during such processes, are then markedly diluted through their dissipation throughout the much larger volume bulk oil medium. Interestingly, Totani et al. [21] found that oxidation was active at the oil/air interface of bubbles produced by foods being fried in a canola-soybean oil blend according to deep-frying practices. Marked decreases in the O_2_ content of these oil blends commenced at a temperature of 120 °C; however, on being allowed to cool at ambient temperature, a slow restoral of these pre-diminished O_2_ levels was found in oil blends pre-heated at a frying temperature of 180 °C. Therefore, it appears that intermittent cooling periods involved in the repeated use of frying oils during recycling frying episodes facilitate their absorption of atmospheric O_2_.

#### 2.4.1. Acrolein 

Acrolein is generated during the frying, cooking or processing of lipid-containing foods [22,23,24], especially those rich in ω-3 FAs such as oily fish products, and also artefactually peroxidised dietary fish oil provisions or supplements in general [25]. An estimated mean concentration of acrolein of 0.51 mmol/kg was found in samples of five types of cooking oil heated to 80 °C and aerated for a period of 20 hr. [26]. Notably, in view of its high volatility, this unsaturated aldehyde was detected in emissions arising from *n* = 4 heated cooking oil products in China [27] at levels varying from 49 µg/L in peanut oil to 392 µg/L in rapeseed oil (the latter oil has a relatively high content of the ω-3 FA linolenic acid (as linolenoylglycerols), one major PUFA source of this aldehyde). It should also be noted that selected ingredients present in commercially-available breading systems and batter can also give rise to acrolein in fried food matrices [24]. 

#### 2.4.2. HNE and HHE

Estimates of the concentrations of HNE alone in French fry samples collected from *n* = 6 U.S. fast-food restaurants [28] were found to range from 8 to 32 μg/100 g (0.51 to 2.05 μmol/kg), values corresponding to 12–50 μg for a standard ‘large’ sized 154 g serving. Moreover, assuming a mean frying oil uptake of 12% (w/w) (range 1-33% (w/w) [3]), our laboratory’s ^1^H-NMR-based estimate of the mean HNE content of 154 g potato chip portions is *ca.* 30 µg, a value which is in very good agreement with those found in [28] (assuming no chemical reactions of this LOP with potato chip biomolecules, e.g. , proteins and amino acids, which is, however, unlikely). Furthermore, these determined values are not dissimilar to the above Korean estimates. Our estimates have also confirmed that HNE accounts for only ≤ 1% of the total molar α,β-unsaturated aldehyde content of fried potato chips (relative amounts of 4-hydroxy-*trans*-2-alkenals found in thermally-stressed sunflower, corn and canola frying oils were <10% of the total measured [12]). Moreover, HNE levels previously determined in sunflower oil were found to be *ca.* 350 and 430 μmol/L when it was thermally-stressed at 190 °C for prolonged 17.5 and 20.0 hr. durations, respectively [29]. These large differences observed between the HNE contents of fried potato chips and the oils in which they are fried are presumably explicable by the higher reactivity of 4-hydroxy-*trans*-2-alkenals with HNE-scavenging potato chip biomolecules than that of *trans*-2-alkenals and *n*-alkanals, and/or an enhanced level of their degradation or further oxidation therein when expressed relative to those for the other aldehyde classes detectable.

Human exposure to 4-hydroxy-*trans*-2-alkenals in vegetable frying oils, fish and shellfish in Korean diets has been previously assessed using GC/MS/SIM as an analytical strategy, along with National Health and Nutrition Survey data to evaluate dietary intake patterns [30]. From these results, the combined HNE and HHE exposure was estimated to be only 16.1 µg per day (approximately 75% of which was HNE), i.e., 0.3 μg/kg for a mean Korean human body weight of 60 kg. Notwithstanding, the risks posed to humans could not be determined, despite the known toxicological actions of these aldehydes. However, on consideration of their basal tissue concentrations, the researchers involved concluded that the dietary availability of such agents may not present a significant human health risk.

### 2.5. Risk Assessments of Aldehyde Intake in Humans: Estimated Margin of Exposure (MOE) Values 

Acceptable daily intake (ADI) is a very important parameter for the evaluation of risks to humans presented by dietary and environmental toxins, for example, and represents the maximum amount of a chemical substance that can be ingested on a daily basis throughout an entire lifetime with no appreciable health risk. For food additives or contaminants, this ADI parameter is usually computed and then employed to determine its risk status via comparisons of it to mean and associated confidence interval values for estimated human exposure and/or intake levels. However, for food contaminants and additives, the ADI may also be termed the tolerable daily intake value (TDI) value. 

ADI values are usually obtained from the lowest no observed effect level (NOAEL), which is derived from long-term in vivo animal model investigations. Hence, such ADI indices arise from the application of a safety or uncertainty factor to the NOAEL value of the most sensitive testing species. This safety factor, which is most commonly 100, is applied in view of the requirement to allow for ‘between-species’ differences and variabilities, and also those featured in their toxicokinetic and toxicodynamic properties. As an example, and for the purpose of comparative evaluations with dietary aldehydes, in 2010 Tardiff et al. [31] performed a safety evaluation of ingested acrylamide using a ‘state-of-the-art’ physiologically-based toxicokinetic model, and TDI (ADI) values for this food toxin was found to be 40 µg/kg BW per day (equivalent to 2.8 mg for a 70 kg BW human), but for cancer only 2.6 and 16 µg/kg BW per day for this agent and its glycidamide metabolite respectively (equivalent to only 182 µg and 1.12 mg/day respectively for a mean 70 kg BW human). The margin of exposure (MOE) values (equation 1) of aldehydes, and LOPs in general, should be employed in risk determinations, since these consider the benchmark dose lower confidence limit (BMDL_10_), a parameter which represents the lower 95% confidence interval limit of the amount (dose) of an aldehyde to give rise to the occurrence of a toxic effect when expressed relative to that of a control:


MOE = BMDL10 (µg/kg BW/day)/EDI (µg/kg BW/day)
(1)

For acrolein, acetaldehyde and formaldehyde, these BMDL_10_ values are 360 [32], 5600 [33] and 2800 µg/kg BW/day [34] respectively. These values have been previously documented by Ferreira et al. [35], and Peterle et al. [36].

On the basis of these figures, estimated MOE values are 360/33.6 = 10.7 for acrolein; 5600/137 = 40.9 (Europe) and 5600/274 = 20.4 (USA) for acetaldehyde; and 2800/(21–200) = 14 to 133 for formaldehyde. EDI values for acrolein, acetaldehyde and formaldehyde were obtained from Wang et al. [19], [37] and [38] respectively. MOE values which are lower than a value of 10,000 indicate a potential WHO-defined health risk [39].

For the purpose of this Commentary paper, we have also estimated MOE values for the potato chip contents of the most predominant aldehydes derived from the thermo-oxidation of linoleoylglycerols, and the fragmentation of their hydroperoxides (Table 2). These estimates were computed on the assumption of humans consuming a single specified potato chip servings daily, but these can be readily adjusted to those consuming averages of 2 or 4 such servings per week my multiplying by the 2/7 and 4/7 factors for amounts available, and corresponding 7/2 and 7/4 ones for MOE estimates, respectively. Clearly, these estimates are lower or strikingly lower than the WHO limit of 10,000 reported (especially for the α,β-unsaturated aldehydes), even when considering an average two portion intake per week. 

Estimated amounts of aldehydes (mg) and contents (ppm) for typical fried potato chip portion sizes of 71, 154 and 400 g (proportionate acrolein mass-equivalent values are provided in brackets). These values correspond to the most predominant aldehydes derived from the thermo-oxidation of linoleoylglycerols. Margin of exposure (MOE) values for aldehyde contents were estimated using the acrolein-equivalent mass values only, and assuming that each potato chip portion represented a mean daily intake for those with a high level of fried food intake. The BMDL_10_ value of acrolein was used for the *trans*-2-octenal and *trans,trans*-deca-2,4-dienal estimates, and that for acetaldehyde was used for the *n*-hexanal one.

## 3. Fate and Adverse Health Effects of Primary and Secondary LOPs in Humans and Animal Model Systems Following Dietary Ingestion 

### 3.1. Lipid Hydroperoxide Aldehyde Precursors (CHPDs and HPMs): Gastrointestinal Interactions, Metabolism and Biotransformations, In Vivo Absorption, Toxicity and Deleterious Health Effects 

Lipid hydroperoxides can potentially give rise to a series of intestinal disorders, including colorectal cancer [40], and their ability to interfere with both molecular and cellular processes, and hence exert a clinically significant impact on intestinal integrity, is responsible for these actions.

Historically, investigations focused on an exploration of the acute toxicity of highly purified methyl linoleic hydroperoxide (MLH) were performed by Cortesi and Privett as early as 1972 [41]. Indeed, the median lethal dose (MLD) value of intravenously (*i.v.*)-injected MLH was found to be 0.70 mmol/kg (*ca.* 230 mg/kg) of body weight (BW) in adult male rats. However, single oral dosages of this agent, which were 10-fold higher than those administered via the *i.v.* route, gave rise to no observable deaths in these experimental animals, an observation indicating either their failure to be absorbed in vivo, or their metabolic modification within the gastrointestinal (GI) tract (e.g., reduction to conjugated hydroxydiene species of a much lowered toxicity), accounted for this phenomenon. For the *i.v.*-treated animals, the major adverse effect observed was localized within the lungs, which enlarged from fluid accumulation and oedema; fatalities therefore arose from severe lung congestion and injury. 

However, it is important to note that since the above level of orally-administered MLH far exceeds that of the estimated daily human intake of lipid hydroperoxides, which is 1.5 mmol/kg (equivalent to 21.4 µmol/kg BW) [42]. 

In an earlier study [43], daily *i.v.* injections of a more realistic, lower dose of MLH (50 mg/day), or its continuous infusion at a rate of 206 μg/min., to experimental rabbits, was found to markedly diminish their α-TOH stores. Following 10–14 days, the injected animals displayed significant fatty degeneration and necrosis of the liver, along with creatinuria, and an accelerated muscular incoordination over those of an untreated control group. Although the creatinuria was circumvented by the oral administration of very high doses of α-TOH (100 mg/day), glutathione peroxidase-replenishing selenite exerted no blocking influence on both creatinuria and liver lesion incidence. For the MLH-infused group of animals, an elevated erythrocyte fragility and substantial creatinuria were observed. Therefore, chronic administration of these low MLH doses gave rise to a rapid consumption/degeneration of endogenous α-TOH, together with an increased incidence of deficiency symptoms for this antioxidant. 

A further study explored the metabolic transformations of orally-administered lipid hydroperoxides in carp both *in vitro* and in vivo [44]. Analysis of methyl ester reaction product derivatives arising from equilibration of 13-hydroperoxy-*cis*-9,*trans*-11-octadecadienoic acid with carp ‘acetone powder’ *in vitro* revealed that methyl 13-oxo-*cis*-9,*trans*-11-octadecadienate, methyl 13-hydroxy-*cis*-9,*trans*-11-octadeca-dienoate, methyl 11-hydroxy-*trans*-12,13-epoxy-9-*cis*-octadecenoate, and methyl 9-hydroxy-*trans*-12,13-epoxy-*trans*-10-octadecenoate were the four major metabolites identified, i.e., one oxodiene and one hydroxydiene species from redox routes, and two hydroxy-epoxy acids. Oral administration of U-^14^C-labeled MLH to carp at a level of 0.10 mL/100 g, equivalent to a very high dose of 2.68 mmol./kg, demonstrated that the predominant metabolites found in selected organs were hydroxy-octadecadienoate and oxo-octadecadienoate, with *ca.* 8% of the dosed ^14^C radiolabel remaining in the body following a 24 hr. period. Since hydroperoxy-octadecadienoates were found to be absent from carp organ lipid profiles, these data indicate that linoleate-derived CHPDs (the most common dietary CHPDs) are firstly intestinally redox-transformed to their corresponding hydroxy- and oxo-adducts, and are then absorbed into the fish circulatory system where they have access to essential organs and tissues. Hence, these observations suggest that although CHPDs are not absorbed in vivo, their intestinal primary redox metabolism products are, at least in carp. These observations are supported by further experimental animal system investigations described below.

Additionally, Kanazawa and Ashida [45] studied the catabolic fate of linoleic acid hydroperoxide in a rat GI system in order to determine the molecular nature of LOPs derived from this source, and which are absorbed into the systemic circulation. Low, albeit perhaps dietarily- relevant concentrations of this primary LOP (6.5 or 18 μmol/L) failed to penetrate into the intestines (presumably because of its rapid biochemical consumption prior to reaching this site), whereas higher doses (200 or 800 μmol/L) partially leached into this environment. In ^14^C radiolabel investigations, products generated therefrom comprised conjugated hydroxydienes (~4%), epoxy-ketones (~10%), aldehydes (~2.4%), and ~13% unidentified ^14^C-labelled species, along with 27% of the unmodified peroxide substrate. However, gastric tissue took up 25% of the label, and *ca.* 6% was found in the intestinal lumen and tissue as degraded aldehydes. Administration of an aldehyde mixture dose gave rise to the accumulation of significant amounts of HNE (Section S5), and the less toxic saturated aldehyde *n*-hexanal in the liver after a 15 hr. duration (both these aldehydes are known to specifically arise from the fragmentation of linoleic acid hydroperoxides). Therefore, evidence for the degradation of linoleic acid hydroperoxide to aldehydes in the stomach was provided by this study, and the researchers involved concluded that such secondary aldehydic LOPs are partially absorbed into the circulation.

Fortunately, the human intestinal system is set up with a battery of defense mechanisms to counter the toxicological onslaught of CHPDs from both endogenous and dietary sources, along with other ROS. Such defense barriers include peroxide-scavenging catalase, superoxide dismutase (SOD) and, most importantly for lipid hydroperoxides, the hydroperoxide-neutralising electron-donor thiol compound glutathione (GSH) and its peroxidase enzymes (GPx) [46,47,48]. Intriguingly, the GI tissue network is the only one which has the ability to express all four classes of GPx enzymes simultaneously, and the sole expression of GI-GPx in this system has indicated that it may be exclusively targeted to protect against the adverse in vivo absorption of dietary lipid hydroperoxides, and peroxides in general [49]. 

Interestingly, GI GPx blocks the shuttling of lipid hydroperoxides in CaCo-2 cells [50], which are of much value to intestinal absorption investigations since they differentiate to form a polarized epithelial cell monolayer serving as a physical and biochemical hurdle to low-molecular-mass molecules and ions. Kanner and Lapidot [51] were the first to demonstrate that ingested PUFAs were peroxidised within the gut, and for this purpose they investigated free radical-mediated processes taking place in the stomach’s acidotic environment which could, in principle, promote the generation of CHPDs from these precursors, along with the concomitant oxidation of further dietary substrates. Their results suggested that human gastric fluid serves as a highly appropriate ‘bioreactor’ matrix for accelerating the peroxidation of dietary PUFAs and additional dietary constituents, and also the potential harmful actions of ingested CHPD LOPs. Moreover, they also found that such localized stomach-based oxidation was completely suppressed by the inclusion of plant-derived dietary chain-breaking antioxidants, an observation which demonstrates the protective actions of such agents, and their beneficial health effects in vivo.

Similarly, Tullberg et al. [52] explored the oxidation of cod liver oil lipids during GI digestion, using models involving standardised digestion protocol-matched human digestive juice, and porcine bile and digestive juice media; fish oil mixed with water at a level of 0.13 mg/mL was employed as an initial meal. Malondialdehyde (MDA), HNE and 4-hydroxy-*trans*-2-hexenal (HHE) were analysed in these digests (using liquid chromatography/atmospheric pressure chemical ionization-mass spectrometry), as were free fatty acids (FAs) by gas chromatography-mass spectrometry (GC-MS); HHE specifically arises from the peroxidation of ω-3 FA sources. Results acquired showed that although aldehydic LOP generation was low during gastric digestion, it was enhanced in the duodenal digestive process. Aldehyde generation was accelerated when using human digestive juices over that found using the porcine system. Free FA liberation was only detectable during the intestinal phase of the protocol, and this parameter attained values of up to *ca.* 30%. 

Interestingly, stable hydroxymonoenes and conjugated hydroxydienes generated from the GI-based reduction of lipid hydroperoxides are also available in the human diet, and, like aldehydes, can also be absorbed from the gut into the systemic circulation [53]. Therefore, there is no shortage of controversy regarding their measurement in biofluids and tissues as biomarkers of ‘oxidative stress’ in vivo.

However, despite these considerations, it appears that fried foods, which represent one major source of dietary LOPs, contain little or no lipid hydroperoxide precursors of aldehydes [12], which as noted above also exert a range of toxicological effects when administered via the *i.v.* route in animal model studies [41], and also in *in vitro* evaluations. Such secondary aldehydic LOPs are more stable than CHPDs and HPMs when introduced into complex food and biological matrices (the latter including human biofluids and tissues), which both contain relatively high levels of many LOP-reactive scavenging agents, including hydroperoxide function-reducing electron donors, and aldehyde-consuming amine and thiol functions present in a wide variety of biomolecules of both low- and high-molecular-mass. Moreover, in vivo, enzymes available for the redox interconversion of hydroperoxides to hydroxydienes, along with the oxidation and/or reduction of aldehydes to their corresponding carboxylic acid and alcohol adducts, respectively, are readily available. Additionally, such aldehydes may reversibly react with food alcohols and/or carbohydrates to from hemiacetals and acetals.

### 3.2. Secondary Aldehydic LOPs: Dietary Ingestion, Gastrointestinal Fate, In Vivo Absorption, Metabolism and Toxicological Effects 

**Figure 4:**
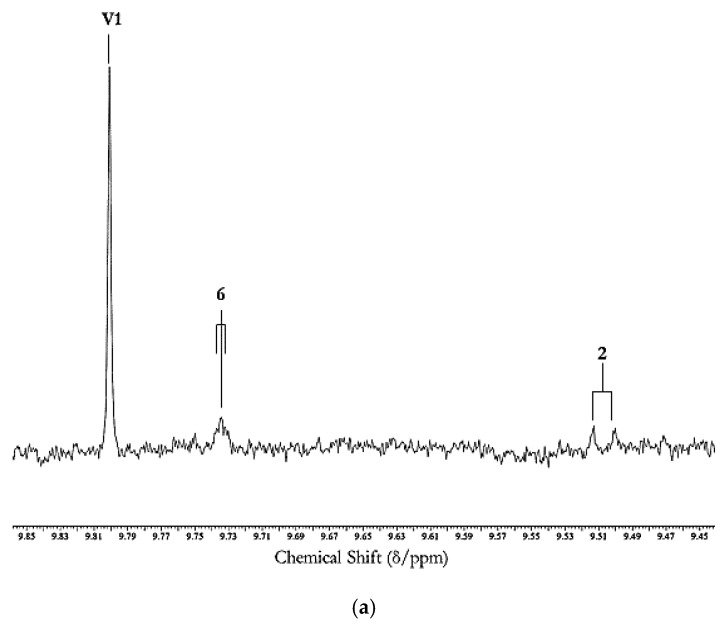

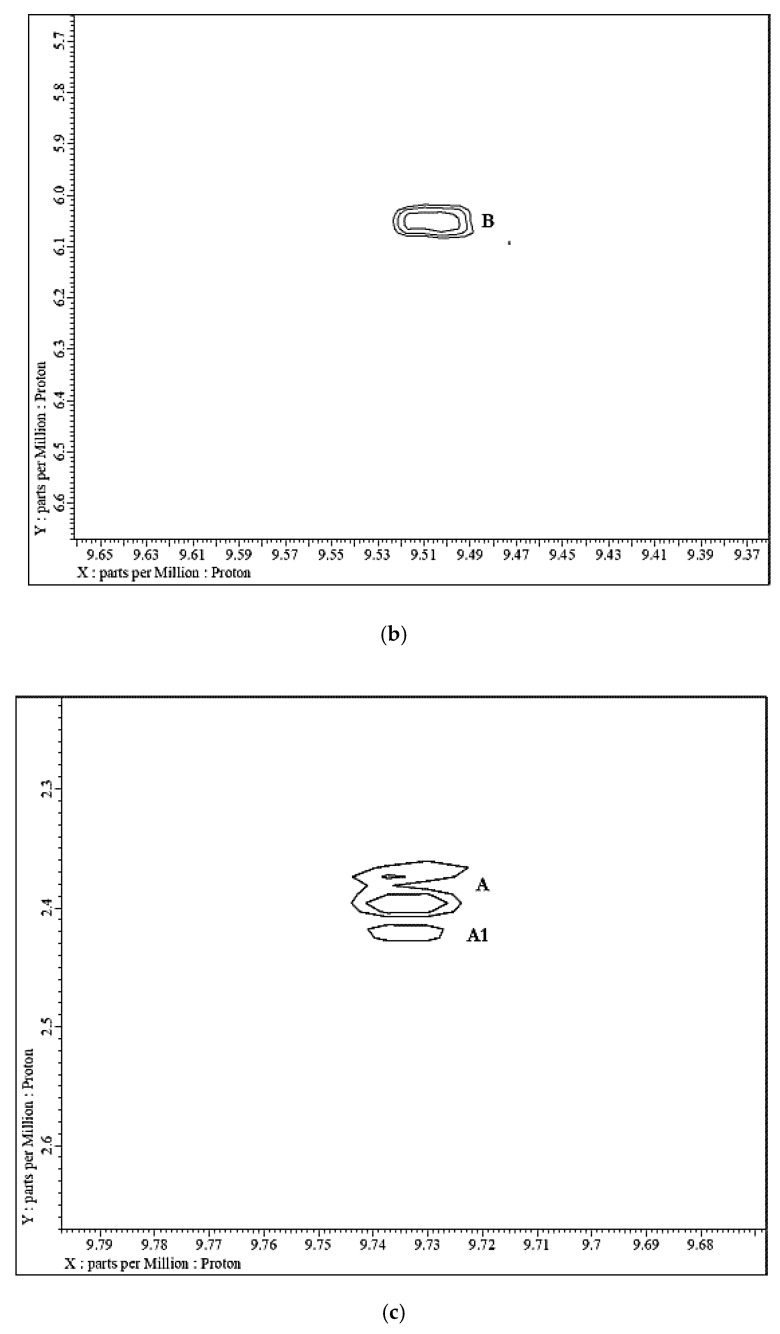

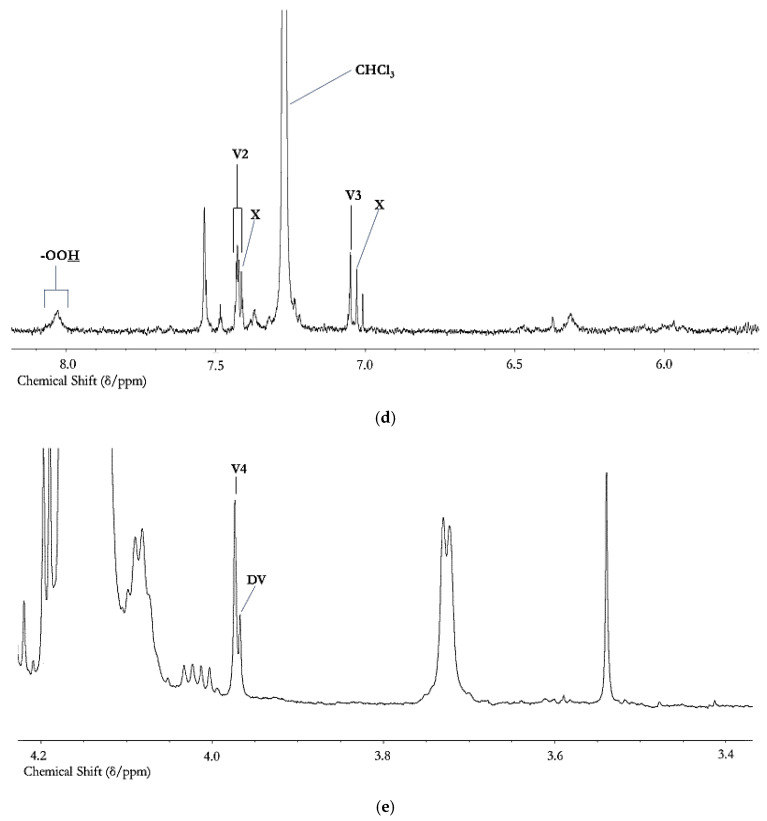
600 MHz 1D ^1^H and 2D ^1^H-^1^H correlation spectroscopy (COSY) NMR spectral profiles of a C^2^HCl_3_ extract of a commercially-available chocolate hazelnut spread product. (**a**) Expanded 9.40–9.90 ppm region of a 1D spectrum of this extract showing an intense –CHO function resonance arising from the flavouring agent vanillin (abbreviated V1), along with ^1^H-NMR-detectable traces of *trans,trans*-2,4-alkadienals (2) and long-chain *n*-alkanals (6). (**b**) Expanded 5.655–6.670 (F1 axis) and 9.364–9.660 ppm (F2 axis) region of a ^1^H-^1^H COSY spectrum acquired on this extract, revealing connectivities between the C1-CHO and C2-CH=CH- resonances of *trans,trans*-2,4-alkadienals. (**c**) Expanded 2.230–2.670 (F1 axis) and 9.668–9.797 ppm (F2 axis) region of the ^1^H-^1^H COSY spectrum shown in (**b**), showing differential molecular couplings between one major (A) and one relatively minor (A1) long-chain *n*-alkanal species. (**d**) and (**e**), Expanded 5.7–8.2 and 3.4–4.2 ppm regions of the 1D spectrum shown in (**a**) respectively, with resonances ascribable to the C5H/C6H (V2) and C2H (V3) aromatic, and C3-OCH_3_ (V4) protons of vanillin indicated. DV represents a tentative assignment to the C3-OCH_3_ function of divanillin, a vanillin oxidation product. Further abbreviations: -OOH, lipid hydroperoxide-OOH function resonance; CHCl_3_, residual chloroform; X, residual chloroform ^13^C satellite.

The molecular nature, toxicities and health hazards potentially presented by aldehydic LOP toxins have been previously explored in some detail, as have analytical strategies available for their determination and monitoring, e.g. , in fried food sources, and human/animal biofluids and tissues, for probing their in vivo absorption, biodistribution, metabolism and urinary excretion (an example of the ^1^H NMR analysis of aldehydes, specifically LOPs and vanillin, in a typical non-fried food product is shown in Figure 4). Indeed, the toxicological and pathogenic properties conceivably arising from the ingestion of aldehydic LOP-containing COs heated according to standard frying practices (in the form of CO-absorbing fried foods for humans), and also aldehyde model systems, include their potential roles in the development and perpetuation of cardiovascular diseases [54,55,56], their carcinogenic [57,58,59,60,61], gastropathic [62], pro-inflammatory [63], and teratogenic properties [64], contributions towards neurodegenerative disorders [65], their hypertensive effects [66]; the development and perpetuation of diabetes [67];and respiratory and pulmonary complications, the latter especially for acrolein [68]; this list is inexhaustive. The inhalation of volatile aldehydes and other carbonyl compounds by workers employed in poorly-ventilated fast-food/restaurant retail outlets is also considered to pose a major threat to human health [69], particularly with reference to established links between an increased incidence of lung cancer and cooking oil fume inhalation amongst such personnel [13,70,71,72]. Indeed, since a wide range of aldehydic LOPs such as acrolein (the lowest homologue *trans*-2-alkenal) have boiling-points (b.pts) below or far below standard frying temperatures (*ca.* 180 °C), cooking oil fumes are very rich indoor air pollutant sources of these toxins.

#### 3.2.1. In Vivo Absorption of and Metabolic/Biotransformation Routes for Aldehydic LOPs 

The GI tract is continually exposed to toxic aldehydes, and subsequent to digestion they are absorbed into the lymphatic system, or directly into the systemic circulation [73]. Indeed, in 1998, our laboratory demonstrated that typical *trans*-2-alkenals generated during the thermal stressing of PUFA-containing frying oils (*trans*-2-pentenal and -nonenal) are indeed absorbed from the gut into the systemic circulation in vivo, then metabolised by a process involving the primary addition of GSH across their electrophilic carbon-carbon double bonds, and finally excreted in the urine as C-3 mercapturate alcohol derivatives, i.e., as *N*-acetyl-*S*-(3-hydroxypentyl)-L-cysteine and -(3-hydroxy-nonyl)-L-cysteine derivatives, respectively, in experimental rats [73]. However, the administered levels of these aldehydes were as high as 10 and 100 mg/kg. Generation of these metabolites also involves reduction of their chemically-reactive aldehyde/aldehyde hydrate (-CHO/-CH(OH)_2_) functions to primary alcohol species via the actions of hepatic alcohol dehydrogenase. These results were consistent with the findings made in [50], which provided evidence for the at least partial absorption of such aldehydes into the circulation.

However, it should also be noted that this study found that at a 16 hr. post-dosing time-point, approximately 15% of the orally-administered dose of *trans*-2-nonenal was oxidatively transformed to its corresponding carboxylic acid metabolite within the stomach [73].

Consistently, following the subcutaneous injection of the simplest *trans*-2-alkenal acrolein to rats, *N*-acetyl-*S*-(3-hydroxypropyl)-l-cysteine was detected and isolated as a key urinary excretion product [74], and these results ae also fully consistent with our ^1^H NMR-based urinary metabolic screening investigations [73], including the hepatic metabolic reduction of the aldehyde functions to alcohol derivatives. However, for these experiments, acrolein was administered by the subcutaneous injection of a 1% (v/v) solution in arachis (peanut) oil into the lumbar region; the vehicle may itself have served as a source of aldehydic LOPs, especially if allowed to peroxidise during periods of storage or solution preparation. 

In a scientifically elegant and highly informative early study published in 1985, McGirr et al. [75] found that a significantly high proportion of dietary MDA is covalently linked to dietary proteins, and an acid-labile urinary metabolite (the N^α^-acetyl derivative of the lysine-MDA enaminal N^ε^-(2-propenal) lysine) was detectable in experimental rats following oral administration of a serum albumin-MDA adduct at a level of 2 mg MDA equivalents/kg BW. Furthermore, this compound was also demonstrated to be a major urinary metabolite of this dialdehyde administered as its sodium enolate salt via stomach intubation. Elevated concentrations of this metabolite were excreted by rats fed a diet rich in highly-peroxidisable cod liver oil. However, these researchers were also able to identify low levels of this metabolite in the urine of fasted rats, and this observation provided evidence that it is also formed as a product derived from the in vivo peroxidation of PUFAs, in addition to its ingestion as a dietary LOP (such as those formed during high temperature frying practices in the human diet), or alternatively, through the prolonged storage of PUFA-containing foods. Injection of MDA as its sodium enolate salt to fasted animals markedly increased its urinary concentration, as expected. In view of the acid lability of N^ε^-(2-propenal) lysine, it is possible that free MDA may be liberated from this primary Schiff base product, and perhaps also from more prevalent dietary protein lysyl residue adducts, in the GI tract (particularly the stomach), so that it may be ingested into the systemic circulation as a free (non-adducted) agent. 

One recent key investigation appears to have resolved the longstanding critical question regarding whether there is some clinically-significant in vivo absorption of 4-hydroxy-*trans*-2-alkenals, potentially one of the most toxic classes of α,β-unsaturated aldehydes available in human dietary sources [76]. Details of this study are provided in Section S5 (Supplementary Materials). 

Since HNE is universally considered to represent a very important secondary LOP, its metabolic fate has been extensively investigated. An exhaustive review of the roles of 4-HNE in health and disease is provided in [77], including a detailed evaluation of its metabolic and biotransformation products. However, important examples of studies of its metabolic fate both in vivo and *in vitro* are also provided in Section S5 of the Supplementary Materials. Interestingly. HNE-modified proteins also appear to be key features of metabolic diseases, and hence offer potential to serve as effective biomarkers for such conditions [78]. 

#### 3.2.2. Associations between Dietary Fried Food Aldehyde Concentration Patterns and Those of Human Blood Plasma: Potential Tracking of Dietary LOPS In Vivo?

In 2000, Mak et al. [79] determined a total of 22 individual aldehydes in circulating arterial blood plasma samples collected from *n* = 8 patients with congestive heart failure (CHF), along with those from an equivalent number of age-matched participants with normal left ventricle (LV) function, i.e., non-CHF controls. Aldehydes were determined *via* a GC/MS bioanalytical strategy, and these included long- and short-chain *n*-alkanals, *trans*-2-alkenals, 4-hydroxy-*trans*-2-alkenals, *trans,trans*-2,4-alkadienals, MDA and the dietary flavouring agent furfural. Mean plasma concentrations, or ranges for the mean aldehyde concentration values of specific structural homologues within each class, are provided in Table 3 for both control and CHF groups, as are full ranges for the individual sampling values found in *n* = 36 samples of potato chips collected from fast-food/take-away restaurant outlets.

The blood plasma results acquired in [79] demonstrated that CHF patients had significantly higher levels of total aldehydes, together with a range of unsaturated ones (specifically, *trans*-2-alkenals and 4-hydroxy-*trans*-2-alkenals, the latter including HHE and HNE), and furfural. Conversely, the normal LV function control group involved had significantly higher levels of *n*-alkanals over those of the CHF patients. Furthermore, the dietary flavouring agent furfural was by far the most predominant aldehyde present, i.e., 37 and 44% of the total aldehydes determined in control subjects and CHF participants respectively) and was found be significantly upregulated in the latter. Furfural is not a LOP, but in addition to its potential genotoxic and carcinogenic properties [73], this food flavourant has been shown to give rise to the accumulation of ROS and cellular damage in *Saccharomyces cerevisiae* [80].

However, aldehydes of the 2,4-alkadienal class monitored in these samples only featured *trans,trans*-hepta- and *trans,trans*-2,4-nonadienals, and the only other di-unsaturated aldehyde monitored was *trans,trans*-2,6-nonadienal. Moreover, *cis*- and *trans*-deca-4-enals were measured as a combined sum. Additionally, this study was complicated by (1) the very high incidences of comorbidities in the male participants recruited to it (mean within-group ages *ca.* 60 years), specifically diabetes, hypertension, and hyperchloesterolemia in both groupings, and (2) medical therapies received by them, i.e., β-blockers, nitrates, ACE inhibitors and calcium channel blockers in both groups, and additionally diuretics in the CHF one. Notably, all vitamin supplements were withheld from participants for a minimum duration of 7 days prior the study, and all oral medications were withheld on the morning of the investigation.

From these results, we therefore elected to perform a comparative statistical evaluation of these blood plasma LOP profiles in terms of the mean molar levels of different classes of aldehydes determined therein expressed as a proportion of the total LOP-relevant aldehyde concentration found in the samples analysed, i.e., those within the above control and CHF groups, to those of the same mean molar ratios of the aldehyde classification contents found in frequently-consumed fried potato chip samples collected from fast-food restaurants (Table 2 and Table 3), specifically those fried in commonly-utilized vegetable oil frying media, as noted in Section 2.3 above. The use of molecular ratio variables for this exercise is, however, quite fortuitous, since they are expected to be less sensitive to the potential influences of a range of latent generic variables such as participant BMIs, ages, etc.

For this purpose, blood plasma levels of furfural were excluded from the computation of proportionate aldehyde contents since it is not a LOP, and nor was it detectable in any of the fried potato chip samples analysed by ^1^H-NMR analysis Unfortunately, it was also not possible to compute the relative proportions of alka-2,4-dienals in the above two blood plasma groups, since *trans,trans*-2,4-decadienal, the major *trans,trans*-2,4-alkadienal arising from the peroxidative deterioration of linoleoylglycerols (Section S2), was not determined in [79], and neither was HHE, the major 4-hydroxy-*trans*-2-alkenal derived from the decomposition of CHPDs generated from the oxidation of ω-3 FAs, e.g. , α-linolenoylglycerols. Short-chain aldehyde concentrations provided in this report were those for *n*-butanal only – since this was the only such analyte included, these values were also removed from the dataset prior to statistical analysis, although they do remain valuable, since such aldehydes predominantly arise from the peroxidation of ω-3 FAs [12]. Therefore, each proportionate aldehyde class considered comprised those of *n*-alkanals, *trans*-2-alkenals, 4-hydroxy-*trans*-2-alkenals and MDA only, and all proportions computed represented the concentrations of each of these LOPs divided by the sum total of them, plus those of all possible alkadienals found. In view of these limitations, results obtained from these comparative evaluations should be treated with some caution.

The mean relative proportions (ratios) of the concentrations of long-chain *n*-alkanals:*trans*-2-alkenals:4-hydroxy-*trans*-2-alkenals:MDA in these three groups of samples were compared and statistically tested for any significant differences between them. Expressed as percentages of the total aldehydes detectable (minus contributions from furfural), these ratios were: 40:31:0.20:0.60 for fried potato chips (mean percentages for a newly-acquired ^1^H-NMR dataset, *n* = 36); 46:30:9:2 for normal LV function (control) subject blood plasma; and 26:39:17:2 for CHF patient blood plasma. Direct comparison of these proportions for the potato chip sample profiles with those of the control blood plasma group showed that although the *trans*-2-alkenal and, to a lesser extent, long-chain *n*-alkanal values were quite similar for this comparison, those of 4-hydroxy-*trans*-2-alkenals were much elevated in the latter, and these data indicate that, in addition to post-ingestional, aldehyde class-dependent modifying factors such as differential rates and extents of their absorption, metabolism, chemical reactivity, protein adduct formation and biodistribution, etc. between each aldehyde class considered, this aldehyde classification appears to arise from in vivo peroxidation processes. Moreover, although the proportionate MDA levels remained small for both these groups, such mean values were elevated approximately 4-fold in the normal LV function blood plasma one.

However, a further major consideration is the dietary availability of all aldehydes considered, i.e., what proportion of them are ‘free’ and what are constituted as adducts with food proteins (as noted for MDA [75]), alternative biomacromolecules, or low-molecular-mass nutrient metabolites such as free amino acids?; such adducts may represent latent sources of these toxins, which may be liberated within the GI system, for example. Notably, our laboratory determines the ‘free’, non-adducted form of these aldehydes in fried food products, and hence our estimated values (Table 2 and Table 3) will presumably represent underestimates of the total taken up from COs during frying practices. 

A permutation testing strategy performed via partial redundancy analysis (PRDA) on the log_10_-transformed proportionate aldehyde level dataset (involving 10^4^ permutations) revealed that aldehyde classification-conditioned differences observed between the three sample groups were statistically significant (*p* = 0.049), as indeed were those ‘between-aldehyde classifications’ (*p* = 0.009), the latter being expected, of course (the log_10_-transformation was required to counteract within-sample negative correlations between proportionate/percentage variables). These significant differences were clearly manifested by 4-hydroxy-*trans*-2-alkenals and MDA being much greater in the normal LV function (control) blood plasma profiles over those of fried potato chips. However, they also arise from the CHF blood plasma group having upregulated proportionate *trans*-2-alkenal and 4-hydroxy-*trans*-2-alkenal levels (over both the control plasma and potato chip serving groups), and significantly higher proportionate MDA concentrations than the fried potato chip group. This significant ‘’between-sample group’ effect observed is also explicable by the large differences observed between the proportionate levels of total *n*-alkanals between the CHF group and the two others compared. 

Therefore, the observation of very similar fractional aldehyde contents of both *n*-alkanals and *trans*-2-alkenals in the large potato chip and smaller control blood plasma sampling groups may serve to indicate that such LOPs are dietary-derived. If this is the case, then the in vivo ‘conservation’ of their proportionate levels may also reflect the overall lower, albeit differential chemical/biochemical reactivities of these classes of aldehydes than those of 4-hydroxy-*trans*-2-alkenals and MDA, following their ingestion by humans. Of particular note, in vivo, *n*-alkanals serve as substrates for pyruvate dehydrogenase, but α,β-unsaturated aldehydes are not affected by this enzyme [81]. Moreover, as noted above, unsaturated aldehydes readily take part in Michael addition reactions with GSH to form their primary detoxification GSH conjugate products [82,83], but *n*-alkanals clearly do not (although they may form Schiff base adducts with the terminal amino function of this tripeptide). However, the rather substantial differences observed between these two groups’ proportionate 4-hydroxy-*trans*-2-alkenal and MDA concentrations certainly indicate, but do not confirm, that such toxins may be generated from in vivo lipid peroxidation processes. 

Proportionate total 4-hydroxy-*trans*-2-alkenal levels in the CHF blood plasma group were also significantly greater than those of the normal LV function control group (*ca.* 2-fold), but this observation was reversed for long-chain *n*-alkanal concentrations, the latter results being consistent with our proportionate levels estimated in fried potato chips. The markedly elevated proportionate value of CHF blood plasma levels of the former class of aldehydes may have been expected in view of an enhanced level of in vivo oxidative stress associated with this condition. However, if this was the case, why was it that the mean level of furfural, a non-LOP dietary flavouring agent, was also significantly increased from 2.45 µmol/L in the control group to 4.06 µmol/L in the CHF one (*p* < 0.01)? Possibly these differences are also partially explicable by differing dietary regimens between these two groups, perhaps an increased level of aldehyde-loaded fried food consumption and/or an enhanced furfural intake in the latter (this flavouring agent is readily absorbed subsequent to administration by any route [84])? Study participants were not fasted for a minimum required period prior to the collection of blood samples in this study, so it certainly appears that such aldehydes may at least partially arise from such dietary, or perhaps alternative exogenous sources. Further possible limitations of the study reported in [79] are detailed in Section S6 (Supplementary Materials). 

However, the authors of [79] also suggested that differences in the aldehyde profiles between their two groups may arise from those between the FA compositions of their diets [73], i.e., with a possible higher peroxidised PUFA and therefore aldehyde content of those received by the CHF one, but they also indicated that such systematic dietary variations between them were unlikely. 

In the control group of participants, the order of decreasing total blood plasma total *trans*-2-alkenal concentrations were (peroxidised FA source(s) in brackets, with L, α-Ln, γ-Ln, O, Po and Ar representing predominant linoleoyl-, α-linolenoyl-, γ-linolenoyl-, oleoyl-, palmitoleoyl- and arachidonylglycerols respectively) *trans*-2-octenal (L) > *trans*-2-hexenal (α-Ln - minor aldehydic LOP, but also a major dietary flavouring agent (Section 3.1)) > *trans*-2-heptenal (O and L) > *trans*-2-nonenal (Po, Ar and γ-Ln, but also a food flavouring agent); that for *n*-alkanals was *n*-heptanal (L) > -nonanal (O) > -octanal (O) > -hexanal (Ar/L, but also derived from the decomposition of *trans,trans*-2,4-decadienal [11]); and that for 4-hydroxy-*trans*-2-alkenals was HHE (α-Ln) > HNE (L and Ar) >>> 4-hydroxy-*trans*-2-decenal (L) ≈ 4-hydroxy-*trans*-2-octenal (unknown peroxidised FA sources). As expected, for the 4-hydroxy-*trans*-2-alkenals determined, the most predominant ones were those arising from the sequential peroxidation of α-linolenoyl- (HHE) and linoleoyl-/arachidonoylglycerols (HNE). 

Using the somewhat broad assumption that a highly significant fraction of at least some of these blood plasma aldehyde levels arise from dietary sources, it should be considered that those found therein represent only residual concentrations, i.e., what remains following their in vivo consumption through their metabolic fate in the GI system, in vivo absorption and then further metabolism thereafter in organs such as the liver, along with any biotransformation of them in human blood plasma and other environments, e.g. , the generation of protein carbonyl species from the reaction of α,β-unsaturated aldehydes with plasma proteins such as human serum albumin and gamma-globulins, and additional Schiff base products arising from the reactions of all possible aldehydes with free primary and secondary amine functions present in selected biomolecules, for example. ‘Between-aldehyde class’ differences in the rates and extents of their consumption will also account for those observed between their relative blood plasma concentrations. In principle, since α,β-unsaturated aldehydes are more chemically-reactive than saturated ones [82], should we perhaps expect higher *n*-alkanal:*trans*-2-alkenal ratios in human blood plasma than what is found in fried food products? Indeed, this ratio is already significantly > 1 in fried potato chip samples (mean ± SEM 1.39 ± 0.10 for our dataset (Table 2); 95% confidence intervals 1.18–1.61, *p* < 0.01) than it is in the culinary oil sources of these aldehydes, and this has been attributed to their differential levels of reactivity with potato proteins, amino acids and further biomolecules between these two classes of LOP toxins [3,14]. This was indeed the case in the above normal LV function group plasma profiles explored, the ratio being 1.53; however, this difference observed was found not to be statistically significant from that found in fried potato chips (one sample t-test). Notwithstanding, it may be conjectured that these proportionately lower control group blood plasma *trans*-2-alkenal levels may also arise from a higher level of reactivity of them in vivo. Interestingly, the fractional blood plasma MDA aldehydic LOP content in these control participants (only 2%) was found to be *ca.* 3-fold greater than that observed in potato chip samples (0.60%). Nevertheless, this observation again confirms that MDA represents only a minor secondary LOP.

Overall, also important is the observation that the total unsaturated aldehyde content of normal LV function patient blood plasma is significantly greater than that of saturated aldehydes, and this is indeed also the case for estimated weight percentage human dietary intakes of these toxins by humans in Refs. [19,85,86], i.e., an unsaturated:saturated aldehyde ratio of 5:2 in mg/kg units [85,86]. Corresponding weight percentage (ppm) values for fried potato chip samples were found to be a very similar value of *ca.* 3:1 [12]. 

A further study performed by Ogihara et al. in 1999 [87], which determined the blood plasma concentrations of secondary aldehydic LOPs in premature infants with and without chronic lung disease (CLD), was, however, limited to only 3 long-chain *n*-alkanals and 4 *trans*-2-alkenals, together with HNE. Full details of this study are available in Section S7 of the Supplementary Materials.

However, it is clear that further research investigations targeted on dietary patterns, human intake, GI fate, absorption, biodistribution and further metabolism of such dietary LOPs are required in order to ratify potential relationships between their dietary availabilities and those detected in human biofluids and tissues. Although not simply conceivable for all patient and age-matched control groups investigated in human trials, it is also thoroughly recommended that for future clinical studies focused on explorations of oxidative stress in vivo (particularly the in vivo generation of LOPs such as reactive aldehydes), all participants involved should be fasted for a sufficient minimal time period prior to the collection of biofluid or biopsy samples for analysis. Such an approach will presumably overcome any interferences or confounding effects arising from dietary LOP sources.

## 4. Atherosclerosis and Its Cardiovascular Disease Sequelae 

Covalent structural modification of lysyl and further selected amino acid residues of the apo-B protein composite of low-density-lipoprotein (LDL) by various aldehydes (including *trans*-2-alkenals, 4-hydroxy-*trans*-2-alkenals, acrolein and malondialdehyde, but not exclusively so) affords its uptake by macrophages to form foam cells, which in turn give rise to artery-blocking fatty streaks. Indeed, Staprans et al. [56] discovered that feeding an oxidized lipid-rich diet to New Zealand white rabbits culminated in a 100% increase in fatty streak lesions within the aorta over those fed an unoxidized lipid control diet. Interestingly, rabbits receiving the oxidized lipid diet were found to have a >100% increase in total cholesterol in the pulmonary artery (predominantly as cholesteryl esters). HNE- and MDA-modified proteins have been previously identified in atherosclerotic lesions using immunological methods and techniques (reviewed in [88]). Such aldehydes, which can also be generated in vivo, are also implicated in a range of pathologies arising from or linked to atherosclerosis, such as their cardiovascular disease sequelae, and complications arising from poorly-controlled types 1 and 2 diabetes.

Major aldehydic LOPs associated with the chemopathology and pathobiology of atherosclerotic oxidant injury have included *trans*-2-alkenals, 4-hydroxy-*trans*-2-alkenals such as HNE, MDA and 4-ketoaldehydes [86], although it should be noted that the most predominant ones present in fried food sources are *trans*-2-alkenals, *trans,trans-*2,4-alkadienals and *n*-alkanals (Section 3.1 [3,14]). Moreover, those arising from dietary sources also include 4,5-epoxy-*trans*-2-alkenals, *cis,trans*-alka-2,4-dienals and hydroperoxy- and hydroxy-*trans*-2-alkenals, for example. As delineated above, all these aldehydes readily react with proteins to form relatively stable protein-aldehyde adducts via Schiff base and Michael addition reactions, and these potential biomarker species may be identified and determined in *in vitro* models involving LDL, and in biosamples collected from animals in models of atherosclerosis, together with those from human patients with potentially enhanced risks, or clinical signs and symptoms of this disorder. Moreover, some aldehydes have been shown to induce intracellular oxidative stress assaults, and also activate stress signaling pathways that exert effects on cellular responses to extracellular stimulating agents [89]. 

Earlier investigations, which focused on exploring the pathological roles of aldehyde-modified proteins have demonstrated that LDL-aldehyde adducts have an enhanced recognition by macrophages, and the uptake of these species is therefore significantly increased in these cells [90]. Moreover, Steinberg et al. [91,92] first recognized that aldehyde adducts of the apolipoprotein B (Apo B) component of LDL transforms this lipoprotein to a pro-atherogenic form which is readily taken up by macrophages to generate foam cells. Further studies which focused on the Apo B moiety of ‘oxidised’ LDL, featured MDA as Nε-(2-propanal)-lysine adducts [93] and 1-amino-3-iminopropene MDA-lysine cross-links [94]; acrolein adducts, including N-(3-methylpyridinium) lysine [95] and 3-formyl-3,4-dehydropiperidine species [96,97]; and HNE-derivatized adducts, e.g. , enaminal class HNE-histidine and HNE-lysine species [98]. 

More recently, Tamamizu-Kato et al. [99] demonstrated that acrolein markedly impaired the functional integrity of Apo E, an exchangeable anti-atherogenic apolipoprotein when present at a 10-fold molar excess, along with heparin-, lipid- and lipid-receptor-binding; experiments were performed using recombinant Apo E, and immuno-blotting employing an acrolein-lysine-specific antibody. These studies are fully consistent with the detection of acrolein in atherosclerotic lesions [96], and acrolein-modified LDL was also found to induce the generation of foam cells from macrophages [100].

Furthermore, it has been demonstrated that reactive aldehydes suppress mitochondrial respiration [101], modify ion-channel conductance pathways [102], and diminish myofilament sensitivity and cardiac contraction [103]. Intriguingly, Wang et al. [19] found that acrolein effectively propagates myocardial ischaemic injury and suppresses nitric oxide (NO^●^)-induced cardioprotection in mice by a mechanism involving attenuation of protein kinase C_ϵ_ (PKC_ϵ_) signal transduction. In 2011, Ishmail et al. [104] found that long-term oral exposure to acrolein, at a level consistent with the human intake of unsaturated aldehydes, gave rise to a dilated cardiomyopathy phenotype in C57BL/6 mice, and from these studies concluded that corresponding effects in humans may be induced by their exposure to this aldehyde. Therefore, human exposure to environmental/dietary sources of acrolein and other α,β-unsaturated aldehydes may provide a rational foundation for heart failure.

Since acrolein induces myotube atrophy *in vitro*, and acrolein-inhalable cigarette smoking serve as major risk factors for skeletal muscle deterioration (atrophy), a very recent investigation [105] focused on the mechanism of this phenomenon discovered that low doses of this aldehyde significantly inhibited myogenic differentiation *in vitro*, a process which may occur through suppression of the serine-threonine protein kinase (Akt) signalling pathway. Mice with or without glycerol-induced muscle injury were exposed to 2.5 and 5 mg/kg BW/day acrolein in distilled water via the oral route for 4 weeks in order to investigate its effects on muscle wasting and regeneration. Acrolein’s ability to induce muscle wasting was confirmed in this animal model system, and muscle regeneration was also found to be retarded. Hence, these data are fully consistent with acrolein’s potential role in the pathogenesis of myogenesis and disease-linked myopathy. At the cellular level, exposure to acrolein exerts a wide range of toxic effects, e.g. , membrane damage, immune dysfunction, endoplasmic reticulum stress, and mitochondrial disruption, along with oxidative stress and protein and DNA adduction [106].

Relationships between the consumption of fried foods in the diet and the risks of cardiovascular and related diseases have recently been extensively reviewed by Gadariju et al. [107], to which readers are referred for further information. This review outlines current evidence available on associations between human cardiovascular diseases, hypertension, diabetes and obesity, and estimates of the fried food consumption of population cohorts. However, data acquired in this survey of many publications focused on this topic were predominantly based on questionnaires to capture fried food intake information, and the study experimental designs involved were limited to case-control and cohort investigations. However, on the basis of this (Ref. [107]) review, there is convincing evidence available to support clear linkages between the risks of these non-communicable chronic diseases and an increased frequency of fried food consumption, i.e., ≥ 4 times per week. 

In the context of metabolic syndrome cluster conditions, further investigations have focused on the effects of diets containing high contents of oxidised frying oils on the development and/or progression of type 2 diabetes, and those conducted by Chiang et al. [67] have demonstrated that such diets can give rise to lowered levels of insulin secretion and hence glucose intolerance. The mechanism for these chemopathological impairments appears to involve an oxidative damage-mediated alteration of glucose metabolism, a process which affects the secretion of insulin by the pancreatic islets. However, such effects were shown to be circumvented by α-TOH supplementation, an observation supporting the role of this chain-breaking lipid-soluble antioxidant in consuming highly toxic primary LOO^●^ radical species, which in turn suppresses the degradation of any lipid hydroperoxides formed to biochemically-reactive aldehydes in vivo. 

## 5. Mutagenicity, Genotoxicity and Carcinogenicity of Secondary Aldehydic LOPs, and Potentially Their Dietary/Fried Food Sources

There is now a long and expansive history of research work which has focused on these area for more than 40 years or so. Indeed, much valuable information is available in this area, with acrolein, MDA and HNE being the most widely investigated, although there are some limitations with direct comparative evaluations of these results in view of the wide diversity of cell lines tested and their tissular sources. Notwithstanding, recent developments in the areas of epigenetic effects, i.e., histon modification and DNA methylation, are very encouraging e.g. , [108]. LOP genotoxicity is extensively reviewed in [109], and the mutagenicities of carbonyl compounds was fully established in the 1980’s.

Moreover, much evidence currently available indicates that aldehydes act as carcinogens [108,109,110,111,112], or in some cases at least have variable degrees of carcinogenic potential. Moreover, a working group of the International Agency for Research on Cancer (IARC) found that aldehyde-containing emissions arising from high temperature frying episodes are “probably carcinogenic to humans (Group 2A)” [111]. The study performed in [112] has provided a high level of evidence that oxidative stress mediated by α,β-unsaturated aldehydes significantly contributes towards cytotoxic and genotoxic cell damage, and these effects are, of course, critically dependent on the structural nature of the agents tested in this manner. Feron et al. [113] performed an overall assessment of the cancer risk status of a range of dietary aldehydes, and from this concluded that although acetaldehyde, crotonaldehyde and furfural do represent dietary risk factors, this was not the case for acrolein, formaldehyde, citral and vanillin. However, it was not possible to evaluate this risk factor for MDA, glycidaldehyde (an acrolein metabolite), benzaldehyde, cinnamaldehyde and anisaldehyde in view of unavailability of sufficient data. These researchers also concluded that dietary-sourced aldehydes should be screened for their mutagenic, cytotoxic and cytogenic activities, and emphasised that such screenings should be prioritised on the basis of their degree of human exposure and expected mechanisms of action, the latter of which is now a rapidly expanding field of study. Notwithstanding, this evaluation review [113] is now very dated, and since that time (1991) there have been major advances in research information and data available on both the dietary availability of these aldehydes, their in vivo absorption and biodistribution, along with their mechanisms of action and target organs, in addition to that based on their exertion of deleterious toxicological and carcinogenic effects. 

Attack of endogenous DNA bases by chemically-reactive aldehydic LOPs can represent very important aetiologies of cancer and human genetic diseases in general. Indeed, such structurally-modified DNA adducts arising therefrom can give rise to frameshift mutations [114]. MDA represents a relatively minor aldehydic LOP arising from the peroxidation of ω-3 FAs containing ≥ 3 carbon-carbon double bonds such as eicosapentaenoic (EPA) and docosahexaenoic acid (DHA) acylglycerol derivatives (containing five and six double bond units respectively), and which are prevalent constituents of marine oils. 

**Figure 5:**
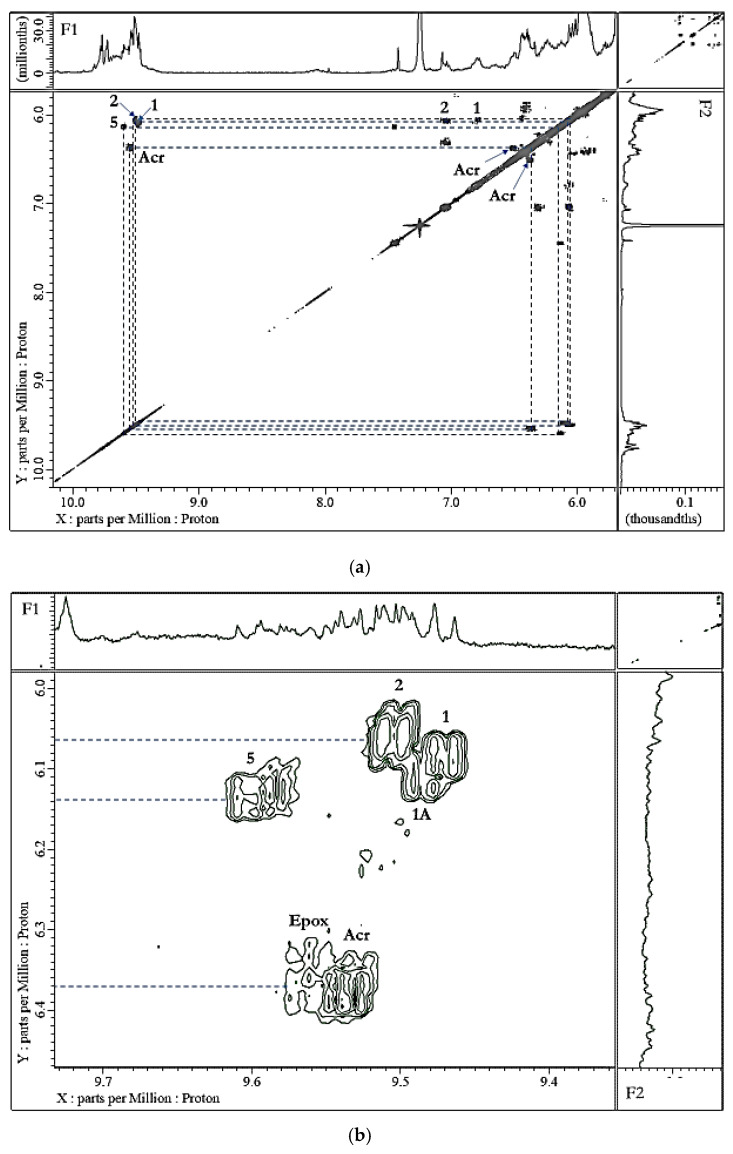

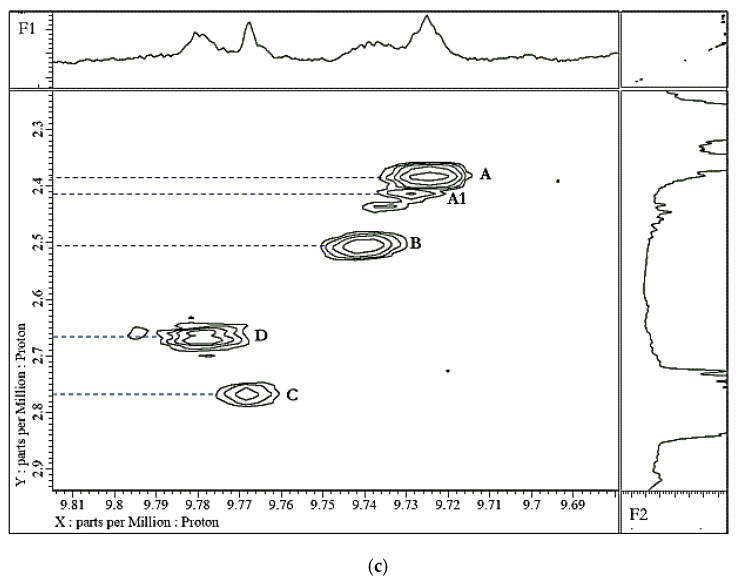
(**a**) 5.7–10.2 ppm regions of single-pulse (1D) and two-dimensional (2D) ^1^H-^1^H COSY spectra of a commercial cod liver oil product exposed to a LSSFE for a period of 90 min. at 180 °C, with ^1^H chemical shift scales (ppm) on the F1 (ordinate) and F2 (abscissa) axes. (**b**) Expanded 5.98–6.47 (F1 axis) and 9.36–9.73 ppm (F2 axis) region of the above ^1^H-^1^H COSY spectrum, revealing linkages between the C1-CHO, C2-CH=CH- (and in some cases C3-CH=CH-) resonances of *trans*-2-alkenals, *trans,trans*-2,4-alkadienals, *cis,trans*-2,4-alkadienals, acrolein and 4,5-epoxy-*trans*-2-alkenals (1, 2, 5, Acr and Epox, respectively). A1 represents a connectivity between the δ = 9.484 and 6.119 ppm resonances, and is tentatively assigned to a *trans*-2-alkenal classification with a significantly different carbon chain length range than that giving rise to the characteristic 9.480 ppm signal. (**c**) Expanded 2.33–2.94 (F1 axis) and 9.68-9.91 ppm (F2 axis) region of the ^1^H-^1^H COSY spectrum shown in (**a**), exhibiting clear distinctions between connectivities arising from three long-chain (A, A1 and B) and one short-chain (D) *n*-alkanal classification. C represents the ^1^H-^1^H correlation for the -CHO and α-CH_2_ function protons of 4-oxo-*n*-alkanals. Samples were prepared for ^1^H-NMR analysis by the method described in [11], and spectra were acquired on the NMR facility described in Figure 2.

As an illustration of the pattern of aldehydic LOPS generated from the peroxidation of ω-3 FAs, Figure 5 shows two-dimensional (2D) 600 MHz ^1^H-^1^H correlation spectroscopy (COSY) NMR profiles of a commercially-available sample of cod liver oil which was exposed to a prolonged (90 min.) episode of thermal stressing at a standard frying temperature of 180 °C in order to explore its peroxidative resistivity. Although CLO products are predominantly employed as dietary supplements and not for frying or cooking purposes, this exposure represents an extreme for the oxidation of ω-3 FAs and further PUFAs therein during increasing periods of storage and exposure to atmospheric O_2_ at ambient temperature, and is also a more analytically-specific approach than alternative, high temperature-dependent methods for monitoring the oxidative susceptibility of such oils products since it has the ability to determine the molecular nature and levels of a wide range of LOPs simultaneously. Data acquired demonstrates the major advantages offered by this technique, specifically the ability to distinguish between, and electronically integrate resonances arising from a variety of aldehydes and aldehyde classes, including acrolein and 4,5-epoxy-*trans*-2-alkenals. Moreover, this analytical approach was also able to provide much valuable information regarding the molecular natures of five or more saturated aldehyde classifications, including both long- and short-chain ones (the latter including ω-3 FA hydroperoxide-derived propanal).

Notwithstanding, such FAs are only present at relatively low levels in most vegetable-based COs, which are rich in ω-6 linoleoyl-, but only limited or deplete in ω-3 linolenoylglycerols, such as sunflower oil with ≤ 0.20-0.30% (w/w) [12]. However, relatively higher concentrations of ω-3 FAs are present in canola and soybean oils, which have approximately 10% and 7% (w/w) of them respectively. Linseed oil is a notable exception, but this oil is far too dangerous to employ for standard shallow- or deep-frying episodes in view of its highly explosive nature, although it is still quite commonly employed for traditional Chinese wok cooking purposes! Fortunately, Belgium and France have regulations which sensibly limit the amount of this FA for use in frying oils to only 2% (w/w) [115], as have some other countries such as Chile.

ω-3 FAs, e.g. , α-linolenic acid-containing acylglycerols in rapeseed, soybean and linseed oils, and those containing EPA and DHA in marine oils, are more susceptible to peroxidation than the linoleoylglycerols predominant in many vegetable oils, and also give rise to a differential pattern of aldehydic LOPs following peroxidation (e.g. , acrolein, 4-oxo-*trans*-2-alkenals and low-molecular-mass *n*-alkanals such as propanal). Therefore, distinction of their 1 and 2D ^1^H NMR profiles from those of ω-6- and ω-9-rich vegetable COs is a relatively facile process. As expected, ^1^H-^1^H COSY linkages for acrolein were not detectable in spectra acquired on thermally-stressed samples of sunflower oil (data not shown). Interestingly, 4-hydroxy-*trans*-2-alkenals were readily ^1^H NMR-detectable in thermally-stressed linoleoylglycerol-rich vegetable oils, together with oleoylglycerol-rich palm oil (predominantly as HNE, with ^1^H NMR signals located at δ = 9.59 (*d*, C1-CHO), 6.82 (*dd*, C3-CH=CH-), 6.31 (*dd*, C2-CH=CH-) and 4.53 ppm (*m*, C4-CHOH) [12]), but not in heated marine oils, in which the corresponding aldehyde generated from fragmentation of such FA hydroperoxides is HHE.

Below, the potential or proven mutagenic, genotoxic and carcinogenic actions of MDA, *trans*-2-alkenals in general, 4,5-epoxy-trans-2-alkenals, and acetaldehyde and formaldehyde, are reviewed and discussed, as are those of acrolein, the latter with special reference to the exposure of humans to wok cooking episodes. Associations cancer risk and fried food intake levels are also described. Section S5 (Supplementary Materials) provides an outline of the toxicological properties and potential adverse health effects of HNE, and its lower HHE homologue. 

### 5.1. MDA

Early studies involving an *E. coli* mutagenesis system revealed that MDA is indeed mutagenic in cells which feature active DNA-repair systems, and these results indicated that this aldehyde had the ability to induce inter-strand cross-linking (fluorescent products were detected from such reaction systems) [116]. Much later, this dialdehyde was found to react with the DNA base adduct guanine to form the exocyclic adduct, pyrimido(1,2-α)purin-10(3*H*)-one derivative (M_1_G) [117], and therefore when MDA is absorbed in vivo [75], it has the ability to generate this derivative. The M_1_G adduct has been detected in selected healthy human tissues, including colorectal mucosa [118], and it induces sequence-dependent frameshift mutations and base pair substitutions in bacteria and in mammalian cells. This finding suggests a potential role for the M_1_G lesion in the induction of mutations commonly related to human diseases. Another early study [119] found that administration of MDA as it enolate anion sodium salt (throughout a 0.1–10.0 μg/g/day dosage level range) to mice in drinking water for a duration of 12 months caused dose-related hyperplastic and neoplastic alterations to liver nuclei, but no gross hepatic tumours were generated. However, addition of MDA to the medium of cultured rat skin fibroblasts gave rise to nuclear abnormalities at added concentrations of only 1.0 µmol/L, despite a cellular uptake of only 4%. MDA/(3-hydroxy acrolein) have been shown to exert cancer-initiating activities in female Swiss mice [120,121].

### 5.2. Trans-2-Alkenals

The mutagenicities of 2-hexenal, -heptenal, -octenal and -nonenal have been previously evaluated in bacterial systems [122], and each of these was found to exert significant effects at µmol/L concentrations; since 2-hexenal occurs naturally in a range of foods, it has received particular focus in such investigations. Testing of these aldehydes, along with 2-pentenal, in V79 Chinese hamster cells at added levels of 3.0–300 µmol/L demonstrated that all of them gave rise to a dose-dependent enhancement in 6-thioguanine-resistant mutant frequency effect, which increased with increasing molecular size [123]. Furthermore, 2-nonenal at doses of only 0.10 and 10 µmol/L was found to give rise to notable sister chromatid exchanges (SCEs), although no chromosomal aberrations, nor micronuclei, were observed in these studies. A further report, focused on *trans*-2-hexenal detoxification and its DNA adduct formation in humans [124], is also featured in Section S8 of the Supplementary Materials. However, although estimates of the mean daily intake of *trans*-2-hexenal for a ‘normal’ diet are 4.2 mg per mean 70 kg BW human, that for a *trans*-2-hexenal-rich diet is as much as 42–147 mg/day [125], a range substantially greater than the 12.5 mg 95^th^ percentile intake estimate of Ref. [124]. 

In 2005, Nadasi et al. [125] evaluated the potential carcinogenicity of *trans*-2-hexenal, for which humans have a dietary pattern-dependent continuous intake, and for this purpose monitored Ha-ras and p53 gene expression alterations, together with tumour development in mice and rats following its administration. For short-term experiments, this study involved CBA/Ca(H-2K), AKR/J(H-2K) and C3He-mg(H-2K) mice (6–8 weeks old) and Long-Evans, Wistar and Fischer 344 rats (6 females and 6 males of each strain), each receiving 3 × 50 mg/kg BW *trans*-2-hexenal in a corn oil vehicle orally (age-matched controls received the same volume of unspiked corn oil). Animals were autopsied 24, 48 and 72 hr. following administration of the aldehyde or its vehicle alone. However, in a long-term study, mice and rats received 150 mg/kg body weight of *trans*-2-hexenal in total intraperitoneally (*i.p.*), i.e., 50 mg/kg on the 1st, 8th and 15th days of the investigation, and were then autopsied following an 18-month survival period. Any developed tumours were removed and 5-µm formalin-fixed, and paraffin-embedded sections were routinely stained by haematoxylin/eosin, and then examined by light microscopy. In the short-term study, no gene alterations were noted 24–72 hr. post-administration. However, *ca.* 14% of the 72 mice and rats within the long-term study were found to develop malignant tumours at the 18-month time-point follow-up evaluation. Therefore, despite exerting no effects on the expression of both onco- and suppressor genes, this reactive α,β-unsaturated aldehyde displayed a significant carcinogenic potential, which is potentially explicable by its epigenetic effects, i.e., they appear to be non-genotoxic carcinogens; in general, consistencies between the genotoxic and carcinogenic effects of compounds is only *ca.* 90% [125]. This may serve to explain the lack of genotoxic risk found for this aldehyde reported in [124].

A further noteworthy point is that since *trans*-2-hexenal arises from other food sources such as fruits, especially bananas [125], its overall total daily human intake is expected to be inflated by a consideration of fried food consumption, which may sometimes exceed more than one fried meal per day, and also possibly the human consumption of serving portions of fried food sources of this α,β-unsaturated aldehyde greater than 154 g. However, this agent is not one of the more predominant ones derived from the peroxidation of UFAs [12]. 

A study which reported comparisons of the cytotoxic and mutagenic properties of the natural product 2-cyclohexene-1-one with those of a range of dietary aldehydes is discussed in Section S9 (Supplementary Materials). 

### 5.3. Acrolein and Chinese Wok Cooking

Early investigations demonstrated that both *i.p.* and intravesicular administration of acrolein to rats gave rise to anomalous levels of cellular proliferation and hyperplasia of bladder urothelium and epithelium [126]. Furthermore, a greater incidence of an abnormal DNA (2-deoxyadenosine)-acrolein adduct has been found in liver [127], oral [128] and bladder cancers [129]. Further information regarding the carcinogenic potential of acrolein as an inhaled or ingested toxin are outlined in Section S10 (Supplementary Materials).

One epidemiological study reported in 2013 found an elevated incidence of lung cancer in non-smoking Chinese women who cooked/fried food at very high temperatures using a traditional Chinese-style wok process, and hence had a high level of exposure to cooking oil fumes arising therefrom [130]. Supporting laboratory evidence for this hypothesis was provided by the observation that these women participants excreted significantly higher creatinine (Cn)-normalised concentrations of acrolein and crotonaldehyde (the latter the next higher *trans*-2-alkenal homologue from acrolein) as their mercapturate metabolites. No differences were found between these groups for corresponding urinary levels of benzene mercapturate. In their conclusion, the authors therefore recommended that domestic kitchen proprietors/users should act to alleviate human exposure to toxic and carcinogenic cooking oil fumes generated during traditional wok cooking styles by ensuring that these areas are sufficiently ventilated at the sites involved. A similar recommendation should, of course, also apply to all commercial/restaurant cooking sites, albeit to a greater, more expansive extent. Highly detailed reviews of the molecular mechanisms featured in acrolein toxicity is provided in [106] and [131]. Information regarding the potential carcinogenic properties of *trans,trans*-2,4-decadienal present in linoleoylglycerol-rich cooking oil fumes is available in Section S11 (Supplementary Materials). A related epidemiological study performed in 2002 [132] is related in the Supplementary Materials (Section S10). Notably, wok frying practices are also expected to markedly promote the oxidative deterioration of PUFAs, since these stir-frying approaches generally use only small volumes of oil per food portion (say, 10–40 mL), and therefore the oil surface area is very large, and exposure to atmospheric O_2_ is maximised. Moreover, trace catalytic transition metal ions from fried foods will promote both the peroxidation of UFAs, and also the breakdown of CHPDs and HPMs to aldehyde fragments, etc. Similarly, adventitious trace metal ions such as Cu(II) derived from the wok metal alloy material itself may provide catalytic sources for these processes.

Intriguingly, when applied dermally to mice and rats, acrolein’s glycidaldehyde metabolite exerts carcinogenic properties [106,133]. Additionally, acrolein is a major lung cancinogen present in cigarette smoke [134]. Vinyl chloride, which is structurally related to acrolein, has been identified as a carcinogen in both animals and humans [135]. In cell culture experiments, acrolein can exert cytotoxic properties at concentrations of < 0.1 µmol/L [136].

Major health threats posed by aldehydes such as acrolein and crotonaldehyde present in cigarette smoke are also worthy of much consideration; however, this aspect of aldehyde toxicology is beyond the scope of this work. Notwithstanding, it is important to note that reliable estimates of the amounts of ingestible aldehydes available in single, average-sized servings of fried potato chips are not too dissimilar to those derived from the smoking of a mean daily allocation of 25 cigarettes [137].

### 5.4. Crotonaldehyde

In 1986, Chung et al. [138] orally-administered crotonaldehyde (the next higher α,β-unsaturated homologue of acrolein), which is mutagenic without metabolic activation [139], to F344 rats in drinking water at concentrations of either 0.60 or 6.00 mmol/L for a 113-week period, and histologically evaluated liver tumours in these groups against an untreated age-matched control one. At the lower dose level, crotonaldehyde was found to induce neoplastic lesions in the liver in 9/27 rats; a further 9 had neoplastic nodules, and 2 had hepatocellular carcinomas. At the higher dose level, however, this aldehyde gave rise to severe liver damage in 43% of these animals, with the remaining 57% developing abnormal liver cell foci. Although results acquired also indicated that crotonaldehyde was a weaker tumorigen than the established carcinogen N-nitrosopyrrolidine (NPYR), they provided strong evidence for its carcinogenicity. Indeed, the incidence of liver tumours in rats treated with crotonaldehyde and NPYR at equivalent doses (0.60 mmol/L) was 87 and 33% respectively. α,β-Unsaturated aldehyde concentrations of 0.60 and 6.00 mmol/L are not at all dissimilar to those of total *trans*-2-alkenals found in vegetable-based culinary oils exposed to high-temperature frying durations [12]. In fact, the higher level is lower than those typically determined in repeatedly-used frying oils. Much further information focused on the carcinogenicity of aldehydes may be found in [58] and [140].

### 5.5. 4,5-Epoxy-Trans-2-Alkenals

In 2017, the FGE.19 EFSA Panel on Food Contact Materials, Enzymes, Flavourings and Processing Aids concluded that ‘4,5-epoxydec-2-(*trans*)-enal (FL-no: 16.071) does raise a safety concern with respect to genotoxicity and, therefore, it cannot be evaluated according to the Procedure.’ [141]. As noted in our studies [12], 4,5-Epoxy-*trans*-2-alkenals represent *ca*. 10 molar % of the total α,β-unsaturated aldehyde contents of PUFA-rich corn or sunflower oils when thermally-stressed according to laboratory-simulated shallow frying episodes at 180 °C.

### 5.6. Acetaldehyde and Formaldehyde

Salaspuro [142] found that aldehyde and alcohol dehydrogenase gene polymorphisms (ALDH2 and ADH respectively) are associated with excessive acetaldehyde exposure, and substantially increase cancer risk in alcohol drinkers, observations which strongly supports the hypothesis that this saturated aldehyde represents a local carcinogen in oesophageal and gastric cancers. Interestingly, acetaldehyde can be classified as a tertiary LOP, since it arises from the deterioration of isomeric alka-2,4-dienals [143], or 2,3- or 4,5-epoxyaldehydes [144,145] during high temperature frying episodes. Section S3 (Supplementary Materials) provides information on dietary sources and estimated dietary intakes of acetaldehyde and formaldehyde, most notably alcoholic beverages for the former. A further study involved human cells collected from patients with a faulty copy of the BRCA2 breast cancer gene to investigate mechanisms associated with aldehyde-mediated cancer induction [146], and the investigators found that formaldehyde exposure leads to the degradation of cellular BRCA2 protein. In those with one faulty copy of its gene (approximately 1 in 100 humans), this process reduces this protein’s concentration below that which is deemed sufficient for efficient DNA repair; this process therefore facilitates the induction of cancer. 

The International Agency for Research on Cancer (IARC) has classified formaldehyde, another known aldehydic LOP, as a human carcinogen [147]. Moreover, in 2011, the National Toxicology Program, an interagency program of the Department of Health and Human Services also classified formaldehyde as a known human carcinogen in its *12^th^ Report on Carcinogens* [148].

### 5.7. Impact of Fried Food Intake on Cancer Risks in Humans 

Particularly notable are epidemiological studies focused on the impact of fried food intake on cancer risk. In 2013, Stott-Miller et al. [149] explored links between the male human consumption of fried foods and prostate cancer risk, and following a review of dietary intake data from more than 3000 participants, found that this condition was more prevalent amongst those who frequently consumed deep-fried foods, particularly French fries, fried chicken, fried fish and doughnuts. These results provide strong evidence for a relationship between fried food intake and prostate cancer risk; results were found to be more highly significant for a more aggressive disease status. Additionally, a meta-analysis of published data [150] found that greater fried food intakes induced an estimated 35% enhancement of prostate cancer risk. 

Knecht et al. [151] found evidence for a positive association between the human intake of fried meat and combined breast, endometrium and ovarian cancers in women, i.e., female hormone-related cancers. Moreover, Bosetti et al. [152] investigated the role of fried food intake on laryngeal cancer risk in a case-controlled study focused in Italy and Switzerland (>500 and 1200 cases and negative hospital controls respectively), and discovered a significantly elevated risk for participants who had high consumption rates of fried potatoes (odds ratio 1.9), meat (1.6), fish (3.1) and eggs (1.9). 

Both genotoxic and carcinogenic risks linked to the ingestion of repeatedly-boiled sunflower oil were investigated by Srivastava et al. [153], and this study found that its oral administration to Wistar rats resulted in a dose-dependent induction of aberrant cells and micronuclei; such dosing also depleted antioxidant enzyme availabilities. Moreover, this treatment also influenced hepatic foci, along with significant decreases in liver mass. 

Woutersen et al. [154] reviewed both animal model and epidemiologic studies focused on the effects of dietary fat consumption on the risks of breast, colorectal, pancreatic, and prostate cancers, and found that its increasing intake exerted a significant influence on prostaglandin and leukotriene biosynthetic routes, and that these properties represented a universal mechanism for such adverse effects. These researchers also reported that the 50% lethal dose (LD_50_) values for acrolein in rabbits and mice were 7 and 40 mg/kg, i.e., there is a wide ‘between-species’ sensitivity to this α,β-unsaturated aldehyde. 

## 6. Potential Mechanisms for the Toxicity and Health Effects of Dietary Aldehydes 

Saturated aldehydes (both short- and long-chain) act as ‘hard’ electrophiles, exerting their toxic actions through chemical reactions with the primary and secondary amine functions, for example that of protein lysyl or histidyl residue side-chains. However, α,β-unsaturated aldehydes and additional alkenals, along with α-oxoaldehydes, act as ‘soft’ electrophiles which preferentially react with ‘softer’ thiol/thiolate functions within protein, peptide (critically GSH) and free cysteine residues. In general, chemopathological mechanisms available for the toxicity of LOPs can be defined as either direct or indirect. Direct mechanisms include the formation of adducts with biomolecules, e.g. , reactions of aldehydes with biochemically-critical proteins and DNA in vivo (i.e., adduction routes), whereas indirect mechanisms may involve the albeit secondary triggering of mitochondrial, oxidative, and/or endoplasmic reticulum stress, with special reference to their associations with human diseases and any target tissues and organs affected.

Recently, Xie et al. [155] suggested a mechanism for the cytotoxicities of the two different classes of aldehydes, and these involved protein and/or DNA damage. They stipulated the importance of DNA repair processes as means for protection against damage provoked by less toxic saturated aldehydes, but not by the unsaturated ones, and hence surmised that inactivation of cells by the more toxic latter classes occurs via protein adduct formation. Furthermore, this review suggested that DNA inter-strand crosslinks, but not DNA-protein crosslinks, nor double-strand DNA breakages, are critical factors for DNA damage resulting from aldehyde attack. In addition, it appears that aldehyde cytotoxicity, which is DNA damage-independent, is mediated by the loss of intracellular GSH, which readily traps α,β-unsaturated aldehydes via Michael addition reactions. However, there are only very low, sub-micromolar levels of aldehydes available in vivo (i.e., residual concentrations following their metabolism, or protein/DNA adduct formation), for example those found in blood plasma [79]. Hence, thermodynamically, such diminished concentrations have the ability to only chemically consume an absolute maximum of their equivalent level of intracellular GSH (for 1:1 aldehyde-GSH conjugates), which represents only a very small fraction of the large intracellular pools of this scavenging thiol available in vivo, and which are often much higher millimolar concentrations, e.g. , 5.5–6.0 mmol/L in whole human blood, which is almost exclusively intracellular [156]. Therefore, it is conceivable that such low (micromolar or sub-micromolar concentrations) of aldehydes have the ability to ‘prime’ cells for a cascade of such damaging events following their uptake. 

However, this is certainly not expected to be the case within the GI system, where [aldehyde]:[thiol/GSH] concentration ratios are expected to be much greater (the fate of aldehydes therein is outlined in Section 3.2). However, aldehyde metabolites such as mercapturate conjugates of the α,β-unsaturated classes (both with and/or without their aldehydic functions reduced or oxidised to corresponding alcohol or carboxylate anion, respectively), potentially serve as valuable biomarkers of human exposure to these dietary toxins in biofluids such as human urine and blood plasma, as may be protein-conjugated aldehyde adducts in the latter. Indeed, blood plasma concentrations of both the low- and high-molecular-mass classes of these biotransformation products are likely to be present at much higher levels than the reactive aldehydic precursors themselves in such biofluids, which renders their detection and quantification more responsive to some lower sensitivity bioanalytical techniques. Moreover, mercapturate metabolites are also valuable for the reliable tracking of these aldehydic LOPs in vivo [117]. Interestingly, conceivably higher level thiol-unsaturated aldehyde Michael addition products in vivo, may also serve as latent sources of these reactive and more highly toxic LOPs [157]. It is also anticipated that the bulk of circulating α,β-unsaturated aldehydes may be covalently bound to the cysteine-34 residue of human serum albumin, since this protein represents the major source of thiol/thiolate anion in this biofluid, and is present at a concentration of 30 mg/mL (approximately 0.5 mmol/L, with a near-equivalent thiol concentration). However, alternative albumin amino acid-aldehyde conjugates have been observed for added acrolein in *in vitro* experiments, and these involve the covalent modification of its histidyl and lysyl residues [158]. 

A series of proteomic-based investigations have confirmed that unsaturated aldehydes inhibit functionally-important cellular enzyme activities by specifically targeting active-site cysteinyl residues therein, or more specifically their thiol/thiolate functions. For example, the impairment of glutathione S-transferase P1-1 (GSTP1-1) activity via Michael-type adduct generation at its Cys-47 residue by a whole range of α,β-unsaturated aldehydes and ketones (a critical factor for consideration, since this protein detoxifies xenobiotics through glutathione conjugation processes); suppression of mitochondrial sirtuin 3 (SIRT3) function by reacting with its Cys-280 residue; and inhibition of glyceraldehyde 3-phosphate dehydrogenase (GAPDH) activity by acrolein via its ability to form a Michael addition product at its active-site Cys-152 residue (reviewed in [159]). Notwithstanding, oxidation of thioredoxin 1 is also considered to represent an important mechanistic factor for consideration. 

Aldehydes readily diffuse across cellular membranes in view of their amphiphilic structures, and therefore have the capacity to covalently react with and hence modify the structure of biomolecules located within the cytoplasm and nucleus, for example. Hence, such damage may occur far from their site of generation if formed in vivo [160], and if produced or available extracellularly, they have the capacity to interact with adjacent cells located remotely from sites of UFA peroxidation; in such cases, it appears that plasma membrane proteins serve as the primary targets for aldehyde attack and adduct formation [161]. Such remote attack may be enabled and/or facilitated by the prior generation of Michael addition adducts with GSH or other endogenous thiols, which represent latent sources of these aldehydes [157]. Moreover, both endogenous and exogenous (dietary derived) aldehydic LOPs react with nuclear proteins, and hence attenuate protein expression via chemical reactions with transcription factors [162]. 

Pathologically-significant enzymatic targets for aldehydes critically depend on the cell type involved, and also on the pattern and distribution of such aldehyde-reactive proteins available, along with cell-penetrating aldehyde levels. Aldehydic protein adduct generation will undoubtedly have differential physiological ramifications for different protein targets with differing cellular functions, along with the precise molecular structure of the aldehyde toxin itself. Moreover, the abilities of such secondary LOPs to reach these targets will also be critically influenced by the intracellular availability of low-molecular-mass aldehyde scavengers such as thiols (predominantly GSH and L-cysteine), other scavenging free amino acids such as L-histidine and L-lysine, and biomolecules with free primary or secondary amine functions (e.g., the secondary metabolite dimethylamine). Intriguingly, when present at low (non-toxic) concentrations, selected aldehydes (especially HNE) have the ability to interfere with signal transduction processes, block cellular proliferation and adhesion, and promote angiogenesis, differentiation and/or apoptosis in cancer cell lines by exerting an influence on the modulation of gene expression through the formation of covalently-modified protein and/or DNA adducts [163,164]. These effects appear to represent an example of an aldehyde-triggered cellular ‘priming’ process, which requires only very low levels of these induction agents. 

Although the biomolecular mechanisms of unsaturated aldehyde toxicity are directly linked to their abilities to form adducts at functionally-important, regulatory cysteine residues in key enzymes, and hence abrogate their functions, it appears that the onset of toxicological and associated adverse health effects are not controlled by the impairment of a single protein. Indeed, much data available now indicates that such toxins effectively suppress an electrophile-responsive proteome, which comprises cysteine-directed cell-specific proteins. Such considerations are markedly complicated by the wide range of aldehydes and aldehyde classes identified in both fried foods and their thermally-stressed CO sources. In addition to their differential dietary availability and intakes, such proteome inhibition will be strongly mediated by toxicokinetic parameters, including their GI reactivity and fate, the rate and extent of their in vivo absorption, subsequent metabolism and biodistribution, etc., which, in turn, are determined by their physicochemical characteristics such as electrophilicity and water solubility, etc. Also important is their ability to access such cysteinyl protein residue targets, which can be limited for at least some unsaturated aldehydes which have structural steric hindrance [155,159].

Fortunately, powerful batteries of aldehyde-metabolising enzymes are available for the rapid consumption and removal of aldehyde toxins in the cardiovascular system [165]. However, gene polymorphisms, which modify the efficacies and extents of aldehyde removal, may significantly contribute towards human susceptibilities to exposure to these agents, and allelic variations in the highly polymorphic glutathione S-transferase P has major catalytic function consequences regarding its abilities to metabolise aldehydes, e.g. , acrolein and crotonaldehyde, effectively. Similarly, polymorphisms in aldehyde-reducing aldo-keto reductases, and/or aldehyde-oxidising aldehyde dehydrogenases and cytochrome P450s may also have deleterious health implications. Recent evidence has indicated that a large number of the genes encoding for aldehyde-metabolising enzymes are triggered by selected natural plant products, for example diallyl-disulphide and -trisulphide, and dithiol-2-thione (present in garlic and cruciferous vegetables respectively), and hence these agents and their plant sources may serve to offer humans protection against dietary aldehydic onslaughts [165]. 

### 6.1. Important Considerations for HNE and HNE

In 2019, Sottero et al. [166] critically reviewed the adverse health effects of secondary aldehydic LOPs and oxysterols; however, their considerations of the former were limited to HHE and HNE, which are formed in only relatively low quantities during the thermal-stressing of linolenoyl- and linoleoylglycerols respectively, in culinary frying oils and consequently fried foods, when expressed relative to those of the more predominant α,β-unsaturated aldehydes (*trans*-2-alkenals and *trans,trans*-alka-2,4-dienals). Such 4-hydroxy-alkenals readily diffuse from the gut into the blood circulatory system, as do *trans*-2-alkenals [73] and many additional aldehyde classes. 

Subsequent to the digestion of ω-3 FA-rich foods, significant concentrations of HHE were found to be generated in both static and dynamic *in vitro* systems modelling gastric and intestinal digestion [2,3,4]. Further evidence for the in vivo absorption of dietary sources of both HHE and HNE has been provided by Awada et al. in 2012 [76], who demonstrated that the former aldehyde accumulated in mouse blood after the feeding of these animals with high fat, ω-3 FA-containing diets for a period of 8 weeks. Similarly, this study revealed that mice which were originally fed with HHE had transient elevations in its plasma level. Moreover, enterocytes treated with that this LOP increased its barolateral medium concentrations [76], and taken together, these results provide evidence for its intestinal absorption. Additionally, radioactivity and radiolabelled HNE metabolites have been detected in the urine, faeces, intestinal contents and major organs of rats following the oral administration of tritiated HNE to these animals.

The persistence of α,β-unsaturated aldehydes, including HHE and HNE, following the *in vitro* digestion of food sources of them, particularly within the lipidic phases of digestion products, was confirmed by GC-MS analyses, and this again indicates that they are bioavailable to the GI tract for absorption [167] (further details on this are available in the Supplementary Materials, Section S5). However, aldehydes detectable in the blood and tissues of raw fish may also arise from the actions of lipoxygenases [168].

#### 6.1.1. In Vivo Generation of HNE/HHE from High and Sustained Dietary Supplementation with ω-3 PUFAs?

*In vitro* models have been developed in order to evaluate the adverse in vivo generation of HHE and HNE from dietary ω-3 FA sources, and these effectively mimic the digestion of vegetable cooking and marine oils [2]. Notably, the *in vitro* digestion of metmyoglobin-containing fish oil emulsions gave rise to the production of both HHE and HNE (*ca.* 2 and 7 µM, respectively) within this matrix [169]. Moreover, for salmon loin and minced beef of equivalent lipid contents, the formation of both these aldehydes was determined in such digestive fluids following the gastric and intestinal phases, and the maximal digestive fluid HNE level observed was *ca.* 2 µM for both food classes, whereas intestinal digestion of salmon oil gave rise to a higher concentration of HHE than that observed for minced beef (3.5 *versus* 2 µM) [170].

#### 6.1.2. Influence of CO Consumption on Blood Plasma Levels of HNE and HHE

Hence, it is of much importance to determine the concentrations of dietary-derived aldehydes in human peripheral blood following the dietary ingestion of cooking oil acylglycerol PUFAs, both unheated and those subjected to increasing numbers of high-temperature frying episodes. Indeed, one interesting study conducted by Calzada et al. [171] involved calculation of the plasma levels of healthy adult participants who were supplemented with dietary DHA (200–1600 mg/day) throughout a 14-day period. Although no change in HHE concentrations were observed at doses of 200 and 400 mg/day, progressively significant elevations in these values were observed the higher doses administered (800 and 1600 mg/day), and these reached 60 and 87 nmol/L respectively. However, it appears that these researchers did not perform essential quality checks on the peroxidation status of DHA samples administered in these investigations. Moreover, as noted above, the TBARS test featured in these studies has major artefactual and interference issues associated with its use as a means to determine secondary LOP levels, and the heating stage involved in the form of the test employed in this study was 96 °C for a 60 min. period, which is more than sufficient to peroxidise PUFAs in analytical samples. 

Although it is presumed that such HHE may arise from the in vivo peroxidation of fish oil DHA, it is, of course, conceivable that this secondary LOP was also present in the samples administered to the above experimental animals or humans; unfortunately, it certainly appears that the fish oil diets used for these experiments were not tested for aldehydic and other LOPs prior to their administration, and therefore HHE may itself have been directly administered along with fish oil EPA and DHA. HHE is a product of DHA and not EPA peroxidation [172], and this observation is consistent with that of Nagagawa et al.’s [173].

HNE is detectable at sub-micromolar levels in human cells, tissues and biofluids, and its ‘free’, non-adducted concentrations in human plasma is 3–125 nM; such values have been shown to be markedly enhanced (0.1–1.0 µM) in human disease such as coronary and peripheral artery diseases [174], and rheumatoid arthritis [175].

However, as noted throughout here, 4-hydroxy-*trans*-2-alkenal levels present in used linoleoylglycerol-rich culinary frying oils are always ≤10% of their total aldehyde content, and less so in potato chip samples [12], a likely consequence of the increased reactivity of this and indeed other classes of α,β-unsaturated aldehydes towards food biomolecules such as proteins, peptides and amino acids, along with acetal/hemiacetal-forming carbohydrates and alcohols, over those of less reactive saturated aldehydes. 

#### 6.1.3. Haem Oxygenase-1 Expression and Dietary Marine Oil Supplementation: Potential Beneficial Role of HHE 

Since haem oxygenase-1 (HO-1) exerts protective actions against a range of diseases, the role of ω-3 FAs, which are involved in the induction of its expression both *in vitro* and in vivo, is of much interest. Intriguingly, Nagagawa et al. [173] examined the ability of dietary supplementation of fish oil on the pattern of FAs and their peroxidation products (specifically HHE and HNE) on HO-1 expression within an extremely wide range of tissues (including liver and kidney) and the blood plasma of C57BL/6 mice, and found that both HHE concentration and HO-1 expression were upregulated following institution of this dietary regimen. Such changes were correlated with corresponding increases in DHA but not EPA levels. Overall, these results were proposed to be consistent with the hypothesis that DHA-derived HHE actually induces HO-1 expression, and that this aldehyde may be responsible for the HO-1-mediated protective effects exerted by dietary marine oils when generated from it in vivo.

#### 6.1.4. Cell Signalling by HHE and HNE

Briefly, studies focused on the potential involvements of both HHE and HNE in cellular signalling processes have indicated their roles in the great majority of signal transduction pathways [176,177], including redox homeostasis, and mediation of key transcription factor activities, e.g. , those of nuclear factor-κB (NF-κB), nuclear erythroid-related factor (Nrf2) and activator protein 1 (AP-1) [176,177,178,179]. Undoubtedly, their high level of reactivities with thiol(ate) and amine functions to form Michael addition (both) and Schiff base (the latter only) adducts is of crucial importance here.

#### 6.1.5. Aldehydes as the Dominant Carcinogens Present in Cigarette Smoke

Finally, a very recent observation of much significance has provided a high level of evidence that aldehydes represent the dominant carcinogens present in tobacco smoke which give rise to DNA damage, inhibit DNA repair in tobacco smoke carcinogenesis and also prevent many other tobacco smoke procarcinogens (including 4-(methylnitrosamine)1-(3-pyridyl)-1-butanone and polyaromatic hydrocarbons) from becoming DNA-damaging agents [180]. On the basis of these results, the authors of this paper proposed that toxic aldehydes represent the dominant tobacco smoke carcinogens. As noted in [12], the aldehyde contents of a typical large size serving of restaurant fried potato chips are not very dissimilar to those available for inhalation during the smoking of a 20–25 allocation of tobacco cigarettes [137].

## 7. Targeted Nutrition and Potential Interventional Routes for Eliminating or Alleviating Health Risks Associated with Dietary LOP Intake in Humans 

Potential strategies for alleviating or circumventing health hazards presented by dietary LOPs represents a widespread area for careful consideration. These include viable means for the determent of LOP generation in PUFA-rich frying oils such as their prior supplementation with heat-resistant lipid-soluble antioxidants, or the removal of these toxins via the treatment of repetitively-used COs with selected LOP-targeted filtration aid materials. However, a major drawback to this antioxidant fortification approach is that many studies have provided evidence that naturally-occurring or higher concentrations of plant-derived chain-breaking antioxidants such as α- or γ-TOH, or synthetic ones such as butylated hydroxytoluene (BHT), are only poorly effective in this context in view of the extremely high level of repetitive thermally-damaging peroxidative recycling bursts often encountered during standard high-temperature frying periods that COs are often exposed to [1]. Moreover, at least some of these antioxidants are thermally unstable, and they may also be significantly volatilised at these temperatures (*ca.* 180 °C) [9,73]. In view of these findings, the future availability of more powerful and more thermally-stable antioxidants, including a range of unusual molecules which are not normally considered to act in this capacity (natural or otherwise), may indeed develop into a productive area for future development. 

The employment of currently-available dimethylpolysiloxane polymers, which are surfactants and anti-foaming agents which also limit exposure of culinary oil surfaces to atmospheric O_2_ required for peroxidation, may also be effective for inhibiting LOP generation during deep-frying episodes; the future customised design and synthesis of more efficient or composite-function derivatives of these may therefore promote frying oil safety, along with an extension of the frying reuse periods of CO products. Additionally, technological approaches available involving methylcellulose or alternative ‘barrier’ agents, which block the uptake of LOP-loaded used PUFA-rich oils by foods fried therein [181], may also offer a solution to this critically important public health issue. Indeed, fried potato chip aldehyde toxin contents are strongly and positively correlated with their total lipid contents, i.e., they are related to the extent of frying oil uptake in this food LOP source [3]. Interestingly, our research work has also revealed that CO LOPs are predominantly present in the external batter layer of battered fried foods such as chicken or fish, with little or none detectable in the food component itself (Figure 3), and so this appears to represent a novel means of protecting fried foods against LOP uptake and human intake, if only consumers were prepared to remove the battered covering of such foods prior to eating! Unfortunately, this battered layer tends to serve as a very palatable, savoury and attractive component of such fried meat and fish food products. 

However, a notable and highly plausible prophylactic approach is the dietary supplementation of human fried food consumers (especially those with poor diets, or a high consumption rate of such foods) with suitable aldehyde-trapping therapies. For example, the amino acid L-cysteine, which is equipped with an aldehyde-consuming side-chain thiol/thiolate function [164], and/or suitable chain-terminating antioxidants, although it should be noted that the latter interventional action will only serve to potentially terminate the further generation of directly-ingested lipid hydroperoxides in the GI system, and hence block their degradation to more stable aldehydes and other fragmentation product toxins. Alternatively, prior fortification of this bioenvironment with relatively high levels of such ingested antioxidants may effectively impair the peroxidation of ingested UFAs which can be triggered therein [51,52]. 

One further possible anti-aldehyde strategy involves the antihypertensive drug hydralazine, which reacts with acrolein and crotonaldehyde to form stable reaction products, for example (1*E*)-acrylaldehyde phthalazin-1-ylhydrazone (*E*-APH) and (1*Z*)-acrylaldehyde phthalazin-1-ylhydrazone (*Z*-APH) from acrolein, and (1*E*,2*E*)-but-2-enal phthalazin-1-ylhydrazone (*E*-BPH) and (1*Z*,2*E*)-but-2-enal phthalazin-1-ylhydrazone (*Z*-BPH) from crotonaldehyde [182]. This drug is similarly reactive towards other 2-alkenals, as is its structural analogue dihydralazine. Hydralazine therefore blocks the cellular toxicity exerted by acrolein, and other 2-alkenals arising as secondary LOPs, and in 2011 Leung et al. [183] found that this treatment (described as an ‘anti-acrolein’ initiative, but not exclusively limited to trapping only this 2-alkenal) significantly diminished myelin damage and improved behavioural outcome in an experimental mouse model system of autoimmune encephalomyelitis. 

Alternatively, plausible targeted manipulations of human levels and activities of aldehyde-neutralising enzymes could also serve as a means for combating the deleterious exposures to aldehyde toxins. Indeed, an improved understanding of the biomolecular mechanisms involved in the induction or stimulation of such enzymes with, for example, selected sulphur-containing natural plant products [165], may provide valuable information regarding specific therapeutic targets which, when activated, may offer an enhanced level of cellular protection against the adverse health effects of exogenous aldehydes. 

Notwithstanding, perhaps the best strategic protective approach is for consumers, together with restaurant and fast-food outlet proprietors, to simply employ COs with only limited PUFA contents for frying and cooking purposes. Notwithstanding, the avoidance of fried meals cooked in PUFA-rich oils is not easily achievable when consumers dine in restaurants or purchase take-out fast food products (especially if they request such frying oil identity information from restaurant staff). Such a development will serve as the most palpable, easily instigated, and consumer-controllable approach for directly avoiding or minimizing aldehyde-mediated adverse health effects. One recent study demonstrated that a MUFA-rich algal frying oil, which contained *ca.* 90% oleoylglycerols and only ~ 5% (w/w) PUFAs, generated only very low levels of aldehydic toxins when exposed to both actual and laboratory-simulated frying episodes (deep- and shallow-frying processes respectively) [12].

From all the studies reviewed here, evaluation of the possible health-threatening effects and disease risks of dietary LOPs realistically remains a dauntingly complex task, since these considerations should be made with special reference to recommended maximum human daily intake (MHDI) values for these hazardous agents, i.e., those stipulated by relevant regulatory health authorities and organizations. However, currently documented values are either very limited to selected aldehydic LOPs such as acrolein, outdated, or even inconsistent between regulatory bodies, i.e., very few are available. Similar considerations also apply to outdated or unrealistic estimates for the MHDI values of such toxins, either from dietary or other sources. One approach employed to date, however, is the determination of an ‘acrolein-adjusted’ MHDI index, which has been employed to relate potential values for higher molecular weight 2-alkenals to that available for its lowest homologue class member, acrolein [3,14]: this value is simply determined by dividing the molecular mass of acrolein by those of higher 2-alkenals (e.g. , , linoleoylglycerol hydroperoxide-derived *trans*-2-octenal), and then multiplying this fractional ratio by the MHDI value of acrolein itself. Currently, the authors do not consider this approach to be completely satisfactory for *trans-* and *cis*-2-alkenals, and certainly not so for alternative aldehydic LOP classes such as *n*-alkanals, for example. Hence, the future consideration, establishment and ratification of many currently unavailable MHDIs for LOPs of known molecular identities also represent major demands for action. Consumer concerns regarding the nutritional and health properties of their foods strongly warrant such requirements [184]. 

In view of the above considerations, optimizations of combinations of food processing methods for eliminating or reducing the content of undesirable LOPs will be facilitated, together with corresponding assessments of the safety of fried and convenience foods, with special reference to the ever-changing consumer lifestyles of the global population.

Finally, as a further critically important factor, the multitude of previous investigational scientific reports available which focus on the possible beneficial health effects of dietary PUFAs should be thoroughly revisited, particularly with regard to those featuring feeding trials with human participants, or other related cohort epidemiological or meta-analysis studies. On reflection, it certainly appears that many of these previously conducted studies may be flawed, since the researchers involved have predominantly neglected the potentially substantial confounding adverse health effects associated with the intake of LOPs such as aldehydes, which were undoubtedly present or even prevalent in the oils or diets originally explored in such investigations. 

## 8. Conclusions 

Heating of culinary frying oils at temperatures associated with standard frying practices gives rise to the generation of very high concentrations of cytotoxic and genotoxic aldehydic LOPs from thermally-promoted, self-propagating oxygen-fuelled recycling peroxidative assaults occurring therein. These toxins penetrate into and hence are ‘carried’ by foods fried in such media, and therefore are available for human ingestion. Since the repeated dietary consumption of such LOPs, especially the α,β-unsaturated classes, may pose serious and chronic hazards to humans, the development of strategies for overcoming these threats is of paramount importance. Future clinical feeding trial or epidemiological investigations focused on explorations of the relationships between the incidence and/or severity of selected human diseases (such as coronary heart disease, cancer, etc.), and the frequency and level of dietary LOP ingestion, may therefore serve to decipher and clarify the nature of such relationships. Similarly, previously available reports that PUFA-laden cooking oils are ‘beneficial’ or ‘safe’ for human consumption after being employed for frying or alternative high temperature cooking purposes may be erroneous and inaccurate, since they predominantly fail to monitor or even consider any LOPs therein, nor the major public health threats posed by their human ingestion.

Following their in vivo ingestion, blockage of the activities and functional status of one or more intracellular protective enzymes at critical active-site cysteinyl residues appears to represent the most important mechanism for the cyto- and genotoxicities of unsaturated aldehydes. In general, aldehydes readily cross cell membranes and enter intracellular environments where they may exert such damaging actions. An analysis of the fractional concentrations of four classes of aldehydic LOPs in human blood plasma, a study performed here for the first time (Section 3.2.2), demonstrated that their mean *n*-alkanal:*trans*-2-alkenal ratio was similar to that observed in a fried potato chip dataset, and this may indicate that such aldehyde classes are at least partially dietary-derived, although there are, of course, many limitations to this form of evaluation. However, proportionate circulating levels of 4-hydroxy-*trans*-2-alkenals (including HNE and HHE) and MDA were found to be significantly much greater than those present in this commonly-consumed fried food source, and again allowing for the above limitations, these data suggest that these secondary LOPs arise from in vivo peroxidation episodes. In principle, secondary aldehydic LOPs ingested by humans have the ability to provoke further cellular ROS generation in vivo, a phenomenon which, in turn, may stimulate further aldehydic LOP generation and hence amplify and perpetuate any deleterious health effects inducible. 

The World Health Organisation (WHO) has indeed identified concerns with the toxicological and genotoxic potentials of aldehydes [185]. As an example, in 2002 they reported that there were >30 epidemiological case-control studies focused on populations exposed to formaldehyde (also a known LOP [186]), and their cancer incidence [187]. Whilst identifying significant concerns on the inhalation of aldehydes and linked respiratory tract carcinomas, this report makes little reference to data available in terms of risks arising from food sources. However, non-respiratory tract cancers were detected in populations exposed to the inhalation of this aldehyde, i.e., multiple myelomas, pancreatic, colon and brain cancers, amongst others. Furthermore, the European Union Scientific Committee on Consumer Safety reported on the ingestion of acetaldehyde and its carcinogenicity, reproductive toxicity and genotoxicity [187]; the cancer risk status of this LOP is detailed in Section S3 (Supplementary Materials). 

Similarly, in view of a potential role of *tt-*DDE in human carcinogenesis, and its widespread occurrence in food products, there remain increasing concerns regarding potential associations between dienaldehyde exposure and the development of human cancers [188]. These concerns are now strongly supported by the detection of this aldehyde and other α,β-unsaturated ones in fried foods and thermally-stressed (used) CO sources of this toxin [12], along with high levels detectable in fumes generated from linoleoylglycerol-rich cooking oils [189]. Additionally, a large amount of experimental evidence acquired from animal model system investigations have revealed powerful associations between reproductive and developmental toxicities and exposure to formaldehyde (extensively reviewed in Ref. [190]); such experiments have involved a range of exposure routes and dose levels, in different species.

Notably, since the potential adverse health effects of the low content food process contaminants acrylamide and MCPD derivatives have attracted much significant attention (both in scientific publications and the media), why is it that toxic aldehydic LOPs, which are present in fried foods at much greater concentrations, are not receiving a similar level of consideration? The authors have a high level of public health concern regarding this issue, not least because it is much more widespread, i.e., it is shared by many other researchers engaged in this increasingly important research area. Is it not the right time for health authorities and governmental food standards agencies to warn the public about these very important health threats? 

Critical factors which are most likely to play key roles in determining the nature and level of dietary LOP intake in humans, e.g. , , shallow *versus* deep-frying processes, and particularly their permeation into fried foods available for human consumption such as potato chips, beef patties, battered chicken portions, etc. should be further investigated. The availability for human consumption of high, toxicologically-significant (up to 25 ppm for each class) levels of the predominant classes of toxic aldehydes in servings of fried foods collected directly from fast-food retail outlets/restaurants, including ubiquitous, globally-accessible large chain ones, should also be considered in detail. Moreover, the rigorous establishment of currently unavailable ADIs and MHDIs for the extensive number of dietary aldehydic LOPs is also a key future prospect. Such requirements are of much importance in view of consumer stakeholder concerns regarding the nutritional and health properties, both positive and negative, prospectively offered by contemporary foods and dietary patterns worldwide.

## Figures and Tables

**Table 1:** Sources of environmental aldehydes (adapted from [19], with permission). References for these data are provided in the Supplementary Materials section of [19].

Source		Aldehyde(s)	Concentration
Water	Surface water (irrigation canal)	acrolein	20–200 mg/L
	Non – carbonated bottled water	Formaldehyde, acetaldehyde, nonanal, and methyl glyoxal	1.7–57.5 mg/L
	Carbonated bottled water	Formaldehyde, acetaldehyde, nonanal, and methyl glyoxal	3.9–197 mg/L
	Ground and surface water	Formaldehyde and acetaldehyde	4.5–12 mg/L
	Ozone – purified water	Formaldehyde and acetaldehyde	2–20 mg/L
Foods	Fruits	acrolein	10–50 mg/kg
	Vegetables	acrolein	10–590 mg/kg
	Donuts	acrolein	100–900 mg/kg
	Codfish fillets	acrolein	100 mg/kg
	Cheese	acrolein	290–1300 mg/kg
	Red wine	acrolein	3800 mg/kg
	Vinegar	acetaldehyde	1.06 gm/kg
	Wheaten bread	butanal	51 mg/kg
	Coffee	furfural	255 mg/kg
	Bread	propanal	31 mg/kg
	banana	2–hexenal	2 mg/kg
	Heated butter	2–pentenal	6 mg/kg
	Heated butter	2,4–nonadienal	1.5 mg/kg
	Vanilla	vanillin	23 g/kg
	Lime, peel oil	citral	130 g/kg
	Anise	anisaldehyde	25 g/kg
	Tangerine, peel oil	2,4–decadienal	500 mg/kg
	Heated lard	acrolein	109 mg/L
	Sunflower oil	acrolein	163 mg/L
Cigarettes	Mainstream	acrolein	10–140 mg/cigarette
	Mainstream	crotonaldehyde	18.5 mg/cigarette
	Mainstream	acetaldehyde	619 mg/cigarette
		Total aldehyde	777 mg/cigarette
	Side – stream	acrolein	100–1700 mg/cigarette
Ambient	Urban air	acrolein	0002–0.035 mg/m^3^
	Smoky interiors	acrolein	0.01–0.05 mg/m^3^

**Table 2:** Toxicological MOE Indices for Linoleoylglycerol Hydroperoxide-Derived Aldehydes in Fried Potato Chips.

	Aldehyde Classification		
Potato Chip Serving Size (g):	*trans*-2-Octenal	*trans,trans*-Deca-2,4-dienal	*n*-Hexanal
71 g	1.09 (0.48) MOE: 52.5	1.73 (0.64) MOE: 39.4	0.88 (0.50) MOE: 784
154 g	2.37 (1.04) MOE: 22.7	3.76 (1.40) MOE: 16.9	1.91 (1.08) MOE: 363
300 g	4.61 (2.01) MOE: 12.5	7.32 (2.72) MOE: 9.2	3.73 (2.10) MOE; 187
Estimated Aldehyde Content (ppm):	15.3 (6.8)	24.4 (9.0)	12.5 (7.0)

**Table 3:** Mean concentrations, or concentration ranges of these mean values (nmol/L) of aldehydes determined in the blood plasma of *n* = 8 patients with congestive heart failure (CHF) and *n* = 8 age-matched normal LV function controls by a GC-MS technique (adapted from [79]).

	Non-CHF Controls (nmol/L)	CHF Disease (nmol/L)	Fried Potato Chips (μmol/kg)
Long-Chain *n*-Alkanals (7)	69–573	42–339	19–560
Short-Chain *n*-Alkanals (1)	67	91	nd
*trans*-2-Alkenals (4)	106–527	163–874	0–430
4-Hydroxy-*trans*-2-Alkenals (4)	33–211	16–434	0.5–2.1 [68]
*trans,trans*-2,4-Alkadienals (2)	152–180	148–420	0–443
Malondialdehyde (MDA)	96	101	0–6 *
Furfural	2450	4060	nd

The bracketed numbers in the first (molecular classification) column refer to the number of aldehydes included for each classification specified for the blood plasma samples analysed. Also listed are the ranges of contents (µmol/kg) found for samples of fried potato chips (or French fries) purchased from fast-food/take-out restaurants (long- and short-chain *n*-alkanals, *trans*-2-alkenals, and *trans,trans*-alka-2,4-dienals were determined by our ^1^H-NMR analysis approach, but those for 4-hydroxy-*trans*-2-alkenals are those reported in Ref. [68] using an LC-MS method. However, both 4-hydroxy-*trans*-2-alkenals and furfural are also readily ^1^H-NMR-detectable and quantifiable. * MDA was specifically determined by a modification of the method outlined in [6], which involved the reaction of thiobarbituric acid (TBA) with this dialdehyde to form a pink/red chromophoric derivative, but only subsequent to its relatively specific extraction into an aqueous medium (mean ± SD first extraction efficacy: 78 ± 2%).

## Supplementary Materials

The following are available online at https://www.mdpi.com/2072-6643/12/4/974/s1. Section S1: Epoxy-Fatty Acid (Epoxy-FA) LOPs: in vivo Absorption and Toxicities; Section S2: Overview of Dietary Sources of LOPs; Section S3: Food Sources and Cancer Risks of Acetaldehyde and Formaldehyde; Section S4: Comparisons of the Dietary Availability and Ingestion of Aldehydic LOPs to those of *trans*-Fatty Acids, Acrylamide and Monochloro-Propanediol (MCPD) CO/Fried Food/Lipid Product Toxins; Section S5: in vivo Absorption, Metabolic Fate, Toxicology, and Adverse Health Effects of HNE and HHE; Section S6: Further Limitations of the Mak et al. Study (Ref. [79]); Section S7: Blood Plasma Aldehyde Concentrations in Infants with Chronic Lung Disease; Section S8: DNA Adduct Formation with and Detoxification of *trans*-2-Hexenal; Section S9: Cytotoxic and Mutagenic Potential of the Natural Product 2-Cyclohexene-1-one Evaluated against a Range of Dietary Aldehydes; Section S10: Acrolein as an Inhaled or Ingested Toxin of Carcinogenic Potential: a Special Case for Consideration; Section S11: Dietary Sources, Inhalation/Ingestion, Cytotoxicity and Genotoxicity of t,t-DDE.
