# Supplementary material for: Potential Adverse Public Health Effects Afforded by the Ingestion of Dietary Lipid Oxidation Product Toxins: Significance of Fried Food Sources
Source: Nutrients. 2020 Apr 1;12(4):974. doi: 10.3390/nu12040974 (PMC7254282; doi:10.3390/nu12040974)
Supplement: Supplementary file 1 [file nutrients-12-00974-s001.pdf]

Supplementary Material

Potential Adverse Public Health Effects Afforded by the Ingestion of Dietary Lipid Oxidation Product Toxins: Significance of Fried Food Sources

Martin Grootveld [1,*], Benita C. Percival [1], Justine Leenders [1] and Philippe B. Wilson [1]

[1] Leicester School of Pharmacy, De Montfort University, The Gateway, Leicester LE1 9BH, UK; P11279990@my365.dmu.ac.uk(B.C.P.); justine.leenders@dmu.ac.uk(J.L.); philippe.wilson@dmu.ac.uk(P.B.W.)

* Correspondence: mgrootveld@dmu.ac.uk;Tel.: +44-(0)116-250-6443

S1. Epoxy-fatty acid (epoxy-FA) LOPs: *In vivo* absorption and toxicities

Epoxy-FAs, *i.e.* FA acyl chains with epoxide rings, are generated from the direct attack of reactive LOO• radicals on olefinic carbon sites in both MUFAs and PUFAs, a mechanism giving rise to the generation of oxirane functions and alkoxyl radicals (LO•) [S1]; this process readily occurs during the exposure of UFA-rich edible oils to common frying episode temperatures [9,14]. Major epoxy-FAs are leukotoxin (9,10-epoxy-12-octadecenoate) and its diol derivatives from linoleic acid oxidation, which promote the degeneration and necrosis of leukocytes. Moreover, these toxins have been implicated in the pathogenesis of breast cancer, multiple organ failure, and impairments to the reproductive functions of rats [S2]. Leukotoxins also have the ability to disturb cell proliferation and the respiratory burst of neutrophils *in vitro* [S3]. Additionally, epoxy-stearate arising from the thermally-induced oxidation of oleoylglycerol FA chains [9,14] was found to give rise to cytotoxicity, and stimulate oxidative stress and apoptosis in HepG2 cells [S4], and a further study revealed that oxylipin epoxy-FAs may be involved in soybean-induced obesity in mice [S5]. Notably, high-resolution ^{1}H NMR analysis is a highly valuable technique for the detection and quantification of epoxy-FAs and their derivatives in thermally-stressed COs, including *trans*-9,10-epoxystearate, 9,10-epoxy-octadecanoate, 9,10-epoxy-12-octadecenoate (leukotoxin), 12,13-epoxy-9-octadecenoate (isoleukotoxin), *cis*-9,10-epoxystearate and 9,10-12,13-diepoxyoctadecanoate, along with primary alcohol LOPs [9,14].

Further studies have confirmed that these epoxy-FAs are also absorbed into the systemic circulation in humans [S6], and this consideration has major implications regarding the monitoring of oxidative stress in humans, just as it does for the absorption of dietary-available aldehydes [93].

In assessments of the ability of soluble epoxide hydrolase (sHE) to modify the mode of action and enhance the cytotoxicity of such FA epoxides, Greene *et al.* [S7] classified three groups of these compounds with respect to this determinant, and from their results suggested that long-chain derivatives of these oxidation products (as methyl esters) act as pro-toxins which are metabolised by sHE to their corresponding diols, which exert a higher level of toxicity. Furthermore, both leukotoxin and isoleukotoxin appear to be structurally optimised to serve this purpose. FAs and esters without epoxide, nor vicinal diol functions, were found to not exert any toxic effects. Recently, Wagner *et al.* reviewed the involvement of long-chain FA epoxides in nociceptive signalling processes [S8].

S2. Overview of Dietary Sources of LOPs

S2.1 Red meat, chicken and poultry

Lipid peroxidation represents the major reason for the deterioration of red meats and red meat products, including rancidity, undesirable odours, loss of essential UFAs and consequently toxic secondary LOP development, together with associated changes in meat textural qualities [S9-S11]. Of course, secondary aldehydic LOPs are chemically reactive towards a range of meat muscle biomolecules, particularly proteins, a process which leads to adverse sensory changes and nutritional

quality loss [S12]. However, the peroxidative stability of such meat products is dependent on the balance of pro- and antioxidants present, and the composition of oxidizable lipidic substrates therein, particularly PUFAs, cholesterol, proteins and selected pigments. Following slaughter, animal muscle cells become overloaded with reactive oxygen species (ROS), a process also occurring during refrigerator storage at 2–4 °C.

The secondary phase of lipid peroxidation involving the conversion of lipid hydroperoxides to secondary LOP fragments (Figure 1) occurs during the early post-slaughter stage, with biochemical modifications limited to the transformation of muscle to meat giving rise to optimal conditions for oxidation of the highly-unsaturated phospholipid (PL) fraction of sub-cellular membranes, since this process is no longer tightly regulated [S13,S14].

LOPs detectable in chicken meat may arise from inadvertently-peroxidised chicken feed (albeit very unlikely for hydroperoxides in view of their high reactivities and metabolic transformations), processing and packaging methods, and even heat exposure in chicken coops. Indeed, the presence of peroxidised oils in chicken feed [S15], or metal ions such as catalytic, pro-oxidant Cu(II) [S16] has been found to give rise to an enhancement of lipid peroxidation in the blood serum and liver of these animals [S17].

It also appears that 'warmed-over' flavour (WOF) represents a sensory attribute of LOPs detectable in meat products [S18]. The meat and poultry industries have, for many years, provided pre-cooked meats for the foodservice industry, i.e. airline, restaurant, institutional and military services. Indeed, in recent times severe outbreaks of food-borne diseases, predominantly those caused by undercooked beef, have led to a great deal of interest in the pre-cooking of most, if not all, meats sold to consumers. Implementation of this process effectively pasteurises or sterilises such meat products, thereby offering protection against such diseases. Unfortunately, the pre-cooking of uncured meats (e.g., beef patties) initiates a particular type of iron ion-catalysed lipid peroxidation which primarily involves the highly polyunsaturated muscle membrane phospholipids, a phenomenon giving rise to the rapid development of an off-flavour. Currently, there is no completely reliable method for the suppression of post-cooking lipid oxidation in meat. Moreover, some consumers are completely unable to detect WOF, whereas others exhibit varying degrees of sensitivity. Indeed, WOF is so common that the majority of consumers tend to accept meats with this flavour. Although much research work has been previously conducted in this area, little has been accomplished on the precise molecular nature and concentrations of toxic LOPs such as CHPDs and aldehydes, together with any further secondary LOPs generated. Therefore, it appears that further research work to determine the precise molecular nature and levels of these toxic LOPs in meat samples is required, both prior and subsequent to the development of WOF.

Developments in some modern technologies for the preservation of meat products, and the maximisation of their impacts on lipid stability, include high-pressure processing (HPP) treatments, limited microwave cooking durations, non-ionising UC-C radiation, infra-red heating, and the addition of antioxidant and transition metal ion chelators, etc., are discussed in detail in [S19].

S2.2 Fish products

Fish products are generally more susceptible to lipid peroxidation than meat ones in view of the high levels of peroxidatively-vulnerable ω-3 PUFAs present, particularly DHA and EPA. Although not a major focus of this commentary paper, the generation of cholesterol oxides in fish products are also notable, and also of at least some public health concern [S20].

Stress levels experienced by fish just prior to or at slaughter appears to represent one of the major explanations for deteriorations in flesh tissue, and this process engenders the activation of metabolic pathways which feature the generation of ROS, including lipid hydroperoxides [S21,S22]. Such primary LOPs may represent the main cause of increased peroxidation cascades experienced following death, and also during storage episodes. However, fortuitously these adverse effects may be successfully suppressed via the performance of bleeding episodes, which are able to diminish ROS concentrations and, of course, those of catalytic haemoglobin and related Fe(II)/Fe(III) haem pigment pro-oxidants

[S23]. Similarly, the frozen storage of fish products at –30 to –40 °C also hinders the peroxidation process. Indeed, freezing in this manner appears to retard peroxidative damage to the greatest extent [S24].

Additionally, these studies are also of some relevance to the direct shallow frying of ω-3 FA-laden fish such as tuna, which displays major losses of O-3 FAs, particularly EPA and DHA, when exposed to such high temperatures [S25]. Results obtained concerning the ^{1}H-NMR monitoring of the toxic aldehydic LOP dataset acquired on the CLO products when heated according to laboratory-based thermal stressing episodes at 180°C are also of a more generic significance to the food science and nutrition areas in view of their generation during the shallow frying of fish. Indeed, in one relevant study [S25], it was found that the shallow frying of Skipjack tuna portions of 74 cm length in double-refined sunflower oil for periods of up to 10.0 min. gave rise to losses of 69 and 84% (w/w) EPA and DHA contents respectively. Although a significant level of FA exchange between the oil medium and fried fish may partially account for these observations, this is, of course, also fully consistent with the thermally-induced peroxidation of tuna O-3 FAs as noted here. In general, the shallow frying period employed for fish is dependent upon the mass/thickness of the food product utilised, *e.g.* 10–12 min. for whole fish, and 3–6 min. for finfish fillets at temperatures ranging from 177–191 °C.

S2.3 Dairy products

PUFAs present in milk and milk products are susceptible to peroxidation, especially during pasteurisation processes (72 °C) ultra-heat treatment (UHT) and homogenisation practices employed by the dairy industry to circumvent spoilage and retain taste. Interestingly, a hedonic sensory analysis revealed that pasteurised milk products from grain-fed cows, which had higher LOP values, were preferred over that from cows fed a mixed ration [S26]. However, results from investigations involving analytical LOP and sensory testings, along with sensory characteristics of ω-3 FA-supplemented dairy products, were mixed [S27,S28]. Moreover, ultra-high pressure treatment (UHPT) appears to limit oxidation and hence influence product safety [S29]. Pereda *et al.* [S30] found that such treatment of milk at 300 MPa and a temperature of 30–40 °C gave rise to significantly lower hexanal and malondialdehyde (MDA) levels when compared to that treated at only 200 MPa, or when exposed to a high pasteurization temperature (90°C for a period of 15 s). Acrolein is detectable in some types of cheese, particularly the Egyptian Domiati type, which contains levels as high as 290–1024 μg/g [S31].

S2.4 Grain products

The manufacture of grain-based food products induces the peroxidation of PUFAs therein, although this phenomenon is critically dependent on food water content. For example, superheated steaming processes, which represent techniques for the roasting of rice, has been shown to give rise to significant rises in peroxide value and thiobarbituric acid-reactive substance (TBARS) indices in this food when water activity was not moderated [S32].

Low moisture foods appear to be more resistant to lipid peroxidation, and this observation is best rationalised by the emulsification of lipidic sources therein, a process which enhances the surface area of peroxidizable PUFAs and hence their level of exposure to the essential O_2 reactant. Moreover, food processing water sources may carry significant levels of catalytic transition metal ions (particularly those of iron and copper), which may promote peroxidation processes, along with the decomposition of lipid hydroperoxides to secondary LOPs such as aldehydes. On example involved the manufacture of crackers, in which such hydroperoxides were more stable in low moisture products than they were in bulk oils, meats and emulsions [S33]. Since this delayed the production of the secondary LOP hexanal, either of the above explanations are plausible (Equations S1 and S2, where L represents a metal ion-complexing ligand, and n the number of these complexed to the metal ion centre):

$$\mathrm{LOOH + Fe(II)L_n \rightarrow LO^{\bullet} + OH^{-} + Fe(III)L_n} \qquad \text{(S1)}$$

$$\mathrm{LOOH + Fe(III)L_n \rightarrow LOO^{\bullet} + H^{+} + Fe(II)L_n} \qquad \text{(S2)}$$

Occurrence of the lipid peroxidation cycle during non-thermal high pressure processing methods is pressure-dependent, and to date results acquired indicate that the application of lowered pressures of 200-350 MPa offer a higher level of resistance of food lipids against oxidation [S34]. As expected, replacement of PUFAs by SFAs provides grain-rich shelf foods with extended shelf-lives in view of a diminished level of lipid peroxidation therein. Notwithstanding, newer strategies for the protection of PUFAs against peroxidation involve microencapsulation techniques, in which particles or droplets are encapsulated in carbohydrates, polymers or even protein coatings.

S2.5 Fruits and vegetables

The natural function of *trans*-2-hexenal offers protection to plants against bacteria and fungi [S35]. Indeed, it has the ability to act in this capacity in view of its powerful mutagenic [S36-S38] and genotoxic [S39-S41] actions. Since humans have a permanent intake of this a,b-unsaturated aldehyde from vegetable and fruit products, with an estimated mean intake of 60 mg/kg BW (equivalent to 4.2 mg for a 70 kg average mass human) per day from a 'normal' diet, and of 0.6–2.1 mg/kg BW per day from foods rich in 2-hexenal such as bananas [125], there remains some concern that this genotoxin may play a significant role in human carcinogenicity.

Interestingly, acrolein may be formed during fruit-ripening episodes [S42,S43]. In 1991, Feron *et al.* [113] determined acrolein levels of <0.01 to 0.05 mg/kg in fruit; however, concentrations of up to 0.59 mg/kg were found in vegetables. Notwithstanding, unfortunately this study lacked information regarding sample collection location(s) and date(s), and also the numbers of such samples analysed.

Serrano *et al.* [S44] developed a new static headspace-gas chromatographic-mass spectrometric (SHS-GC-MS) technique to successfully monitor 15 different aldehydes in canned vegetable products, which arise as natural constituents or are present as disinection by-products. These aldehydes were detectable in both the solid and liquid phases of these products, and 13 of these carbonyl compounds were present at mean levels ranging from 2.2–39 μg/kg and 0.25–71 μg/L, respectively. Additionally, glyoxal and methylglyoxal found predominantly in the liquid phase at concentrations of 1.4–4.1 μg/L were attributable to their employment in treated water used for manufacturing purposes.

S2.6 Alcoholic beverages

Acrolein can also arise as an undesirable by-product of alcoholic beverage fermentation, and also from the storage and maturation of alcoholic drinks [113]. Although data available are very limited, a maximal concentration of 3.8 mg/kg acrolein has been documented for red wine [113], and mean levels in samples of United Kingdom lager beer were found to be 1.6 μg/L and 5.0 μg/L for fresh and aged samples respectively [S45]. Such trace concentrations (<10 μg/L) were also measured in a Canadian apple wine product [S46]. This secondary LOP has also been found in a range of non-alcoholic beverages such as tea and coffee, although no concentration information was provided [113].

Astonishingly, the extremely high level of acroleinfound in red wine [113], which is very nearly equivalent to 70 mmol/kg (Table 1, [19]), is greater than the total aldehyde concentration of a very highly thermally-stressed or reused PUFA-rich culinary frying medium such as sunflower or corn oils [14]. Therefore it appears that this commonly consumed beverage presents a number of serious health risks, which will clearly markedly negate any positive health benefits offered by the rich plethora of antioxidants, phenolic or otherwise, contained therein, and which continue to be reported in many scientific publications.

Alcoholic beverages in general serve as major sources of acetaldehyde, which amounts to an estimated 7.8 mg/day for a human of mean BW 70 kg. Full details of dietary sources of this staurated aldehyde are provided in section S3.

S2.7 Aldehydes as food flavouring agents

trans-2-Hexenal, a known secondary LOP, is something of an exception since it is widely used in the food industry as a flavouring agent. and humans have a permanent, and what might be now

considered a very frequent intake of it from vegetable and fruit sources [section S2.5]. Hence, its carcinogenic potential in humans remains a health concern. The 2.4 mg estimate for total *trans*-2-alkenals for a typical single 154 g fried food portion [14] is similar to that estimated for the mean daily human intake of 2-hexenal from such 'normal' diet food sources.

In 2004, a report from the sixty-first meeting of the Joint FAO/World Health Organisation (WHO) Expert Committee on Food Additives (JECFA) [S47] stated that '*α,β*-Unsaturated aldehydes are formed endogenously by lipid peroxidation of PUFAs (Frankel et al., [S48]) or can be consumed as naturally-occurring constituents of food (Stofberg and Grundschober, [S49]; Maarse et al., [S50]), and are only consumed to a minor extent as added flavouring agents.' However, at that point in time, this report was limited by the unavailability of reliable research data, and information regarding the preponderance of proportionately much higher levels of such aldehydic toxins in dietary fried and other food/environmental sources available for human consumption [3,14].

trans,trans-2,4-Decadienal (*t,t*-DDE) represents 50–60% of the total production of linear aliphatic alkadienal flavouring agents in both Europe and the USA [S47], and Section S11 of the Supplementary Materials section provides estimates of its daily human intake as a flavourant. Notably, the potential role of this a,b-unsaturated aldehyde as an indoor air pollutant toxin present in cooking oil fumes has been implicated in the development of lung cancer in restaurant workers, and this is also discussed in Section S11.

Further aldehydes which are added as flavouring agents to a wide range of frequently consumed foods include aromatic classes of these compounds, for example benzaldehyde, phenyl-acetaldehyde, and vanillin and its ethyl ester derivative. An example of the routine ^{1}H-NMR screening of food products for aldehydes is shown in Figure 4, which reveals that high levels of vanillin (3-methoxy-4-hydroxybenzaldehyde), and tentatively also one of its oxidation products, were present in a chocolate-hazelnut spread product, along with much smaller amounts of secondary *trans,trans*-2,4-alkadienal and *n*-alkanal LOPs. Similarly, furfuraldehyde (furfural) is also employed as a food flavourant, and also occurs naturally in a range of agricultural by-products such as wheat bran, oats and corncobs. Interestingly, cocoa and coffee contain as much as 55–250 ppm of this agent [S51], and there is evidence available that this aldehyde exerts genotoxic and carcinogenic actions, especially in the liver [S52]. Further aldehydic food flavouring agents are citral, methional and methylglyoxal [S53].

S2.8 Thermoplastic food packaging materials

Residual solvents derived from the coating, printing and lamination manfacturing practices represent the major sources of undesirable packaging odours, which may also arise from degradation of the polymeric packaging material itself, along with that of any adhesives, coatings and inks employed. Such adverse odours may result from the thermally-induced deterioration of processed packaging matrices, resin oxidation and/or the corona discharge-mediated oxidation of films and/or their additives. In view of these problems, researchers have targeted odour-scavening as a major developmental research area for extending the shelf-life of pre-packaged food products, and a series of anti-oxidant and aldehyde-scavenging agents have been refined and advanced for this purpose, the latter predominantly focused on hexanal. Alternative strategies have involved the development of oxygen-scavenging agents, activated carbon and molecular sieves.

Acrolein is also produced as a thermal degradation product of cellophane and polystyrene thermoplastics used to package foods [S54,S55]; however, to date there are no reports available which suggest its possible transfer to packaged food products.

S3. Food sources and Cancer Risks of Acetaldehyde and Formaldehyde

For acetaldehyde and formaldehyde, estimated mean daily dietary exposure quantities are 93 and 11 μg/kg respectively (equivalent to 5.6 and 0.66 mg, respectively, for a mean 60 kg body weight) in Korea [S56]. In that country, the major contributors towards these values are napa cabbage kimchi (15%), rice wine (11%), and beer (7%) for acetaldehyde (this aldehyde is a very common trace contaminant in alcoholic beverages), and white rice (11%), napa cabbage kimchi (4%) and

ramveon/instant noodles (4%) for formaldehyde. Additionally, for foods and their associated cooking/frying methods, acetaldehyde was found to be most prevalent in macerated dried shiitake, boiled garlic, cheonggukjang/rich soybean paste, and pan-fried yeast. For formaldehyde, however, cooked/prepared foods containing the highest levels of this toxin were dried Alaskan pollack, > dried shiitake boiled following maceration, > broiled dried Alaskan pollack > myeongran jeot/ cooked salted pollack roe > yeast > dried Alaskan pollack.

In 2009, Lachenmeier *et al.* [S57] conducted a highly detailed and prolific investigation of the mean exposure of human populations to acetaldehyde from alcoholic beverage sources alone, and this was estimated as 0.112 mg/kg BW/day (corresponding to 7.8 mg/day for a human of average BW 70 kg). The margin of exposure (MOE) value (defined as the ratio of its no-observed-adverse-effect (NOAEL) value to its theoretical, predicted, or estimated dose/concentration of human intake) was computed to be 498, and the life-time cancer risk was found to be 7.6 in 10,000. However, greater risks may be considered for those exposed to high levels of acetaldehyde contaminations, as noted for unrecorded alcoholic beverage consumption in Guatemala and Russia, and for which possible exposure risks are within the 1 in 1,000 range [S57]. However, both these life-time cancer risk values greatly exceed those of well-known established ones from environmental factors, which generally lie between 10^{-4} and 10^{-6} [S57].

Acetaldehyde is a quite common flavouring additive for many food products, *e.g.*, those of milk (fruit yoghurts), fruit juices, sweets, desserts, margarine and soft drinks, for example [113], and the FEMA has provided consumption level data for this toxin of 0.16 mg/kg BW/day from such flavouring applications alone [S58]. Corresponding estimates from JECFA were 0.16 and 0.18 mg/kg BW/day for the USA and Europe respectively [S59]. Exposures to this aldehyde arising from cosmetic use appear to be virtually negligible [S57]. However, the life-time cancer risk from ethanolic sources, which is metabolised to acetaldehyde *in vivo*, remains much greater than that of alcoholic beverage sources of this aldehyde, specifically 10^{-3} to 10^{-2} [S60].

S4. Comparisons of the Dietary Availability and Ingestion of Aldehydic LOPs to those of *trans*-Fatty Acids, Acrylamide and Monochloro-Propanediol (MCPD) CO/Fried Food/Lipid Product Toxins

Adverse health risks associated with human ingestion of the neuro- and reproductive system toxin, and potential carcinogen acrylamide (*ca.* 0.6 ppm only for potato chip products [S61]) are here considered almost negligible when compared to the estimated levels of cytotoxic and genotoxic aldehydes available for human consumption in fried foods [3,14]. Similarly, potato chip levels of established monochloro-propanediol (MCPD)/MCPD glycidyl ester adduct carcinogens are also very low (0.10–0.26 ppm [S62]). Estimated mean potato chip ppm levels of the most prevalent aldehydes arising from the peroxidation of linoleoylglycerol sources alone are 15, 24 and 13 ppm for *trans*-2-octenal, *trans,trans*-deca-2,4-dienal and *n*-hexenal respectively, whereas those from oleoylglycerol hydroperoxide substrates are 19 and 18 ppm for *trans*-2-decenal and *n*-nonanal respectively [14].

Moreover, the adverse health effects potentially exerted by a variety of aldehydic LOPs have a much broader span than those provoked by the dietary ingestion of *trans*-FAs, which are generally limited to their long-term effects on the development of coronary heart diseases [S63,S64], but not cancer. Additionally, on a mole-for-mole (molar) equivalence scale, the severity of the cytotoxic and genotoxic actions of such LOPs are much more substantial than any deleterious health effects exerted long-term by *trans*-FAs. However, it is important to note that despite this, from 1999 to 2002 the estimated mean US daily intake of *trans*-FAs (6.1 g, equivalent to 2.5% of energy) [S65] was, of course, substantially greater than that of dietary aldehydes; the *trans*-FA intake range within the fifth distributional quintiles was very broad, and ranged from 8.8 to 92.4 g. The major dietary sources of *trans*-FAs were reported to be cakes, biscuits, pies and pastries in this communication.

S5. *In vivo* Absorption, Metabolic Fate, Toxicology, and Adverse Health Effects of HNE and HHE

Animal model system-based investigations have demonstrated that chronic consumption of high levels of toxic aldehydic LOPs such as 4-hydroxy-*trans*-2-nonenal (HNE), a fragmentation product of

linoleoylglycerol hydroperoxides, and also generated in thermally-stressed culinary frying oils containing these precursors (albeit at significantly lower levels than those of *trans*-2-alkenals, *trans,trans*-2,4-alkadienals and *n*-alkanals [14]), amplifies tumour frequencies [13,S66], and significantly contributes towards the metabolic syndrome [S66]. It is also associated with the development and perpetuation of vascular and neurodegenerative diseases, the former including atherosclerotic lesion development [13,S67]. Many of these adverse effects have been observed at concentrations similar to those available *in vivo* (sub-micromolar for HNE). Intriguingly, early studies revealed that HNE has the ability to engender genotoxic events in lymphocytes and hepatocytes [13,128,S68,S69], and also modify cell-cell adhesion, induce reactive oxygen species (ROS) production, and diminish intracellular glutathione stores in lung microvascular endothelial cells [S70].

Awada *et al.* [76] explored the intestinal absorption of both HHE and HNE, and for this purpose they fed diets containing either unoxidized (control) or moderately-oxidised ω-3 FAs (with ω-6 to ω-3 % content ratios of 5–8) to mice, the latter diet containing much higher levels of both aldehydes than the unoxidized one. Other model rats featured in the study received orally-administered HHE against baseline control animals (mice were postprandially euthanized). Further experiments involved the *in vitro* equilibration of human Caco-2/TC7 cells with added HHE and HNE. Intestinal absorption of HHE was indeed observed, and this uptake was found to be related to the formation of its protein adducts (presumably via Michael addition and Schiff-base reactions), and an enhanced expression of glutathione peroxidase 2 (GPx2) and glucose-regulated protein 78, both *in vitro* and *in vivo*. Moreover, the oxidised ω-3 FA diets administered elevated the inflammatory biomarkers interleukin-6 (IL-6) and monocyte chemoattractant protein (MCP-1) in blood plasma, and also activated nuclear factor kappaB (NF-κB).

Therefore, this pioneering work provided powerful evidence for the *in vivo* absorption of 4-HHE and perhaps also 4-HNE; although blood plasma concentrations of both these reactive α,β-unsaturated aldehydes were elevated following oral administration of the oxidised lipid dietary regimen (substantially so for 4-HHE), that observed for 4-HNE was only minor (from 6 to 9 nmol/L), and this increase was found not to be statistically significant.

A summary of key studies focused on the metabolic fate of HNE in cells, organs and experimental animals are provided below:

(1) In packed erythrocytes, metabolic routes found featured a GSH conjugate and 4-hydroxynonenate (70 and 25% of total metabolites respectively) – the glutathione conjugate was subsequently resolved into 2 distinct species, *i.e.* glutathionyl-HNE and glutathionyl-1,4-dihydroxynonene [78].

(2) A differential but limited capacity to metabolise HNE in the lung and the brain, information supporting the propensity for trace amounts of this toxin to give rise to tissue injury within these organs during episodes of *in vivo* oxidative stress [S71]. However, in this investigation, metabolism to GSH conjugates by glutathione-S-transfer was observed, with the highest concentrations being observed in the lungs of rats and the livers of mice; protein-HNE adducts were found in all tissues evaluated, and $NAD(P)^+$ and NAD(P)H were found to substantially promote HNE's metabolism in the liver only.

(3) Siems *et al.* [S72] conducted a highly-intensive study investigating intracellular metabolism of HNE in a very wide range of mammalian cells and organs at added levels of either 1 or 100 μmol/L and a temperature of 37 °C, the former concentration corresponding to that found physiologically. At this lower level, typical blood serum levels of 0.1 to 0.2 μmol/L were found to be restored within 10–30 s, and most of the 100 μmol/L dose was metabolised within a 3 min. period. In hepatocytes and further cell types, the 1:1 GSH-HNE conjugate, along with the corresponding alcohol and carboxylic acid adducts of the added substrate, *i.e.* 1,4-dihydroxynonene, and hydroxynonenate respectively, were the primary products generated. β-Oxidation of the latter carboxylic acid metabolite was also shown. Chemical reactions of HNE with protein functional groups were observed, but only to a low extent (≤ 8%) of the total consumption level. However, generation of the mercapturate, cysteine-HNE and

glycine-cysteine-HNE Michael addition adducts was limited to only neutrophils and kidney cells. The involvement of such pathways provided evidence for antioxidant defence mechanisms in place to alleviate the potentially adverse chemical modification of proteins *in vivo*, *i.e.* those involving the generation of protein-carbonyl and alternative aldehyde-damaged species.

(4) Protein-HNE adducts predominantly involve cysteinyl, histidyl and lysyl residues as the major targets of electrophilic addition at the C-3 position of HNE to form Michael addition products (reviewed in [77]). Such adducts can give rise to major impairments in the normal biochemical functioning of a range of proteins, as documented in [S73]. HNE also has the ability to cross-link these biomacromolecules in view of its capacity to engage in both Michael addition and Schiff base reaction processes. Although Michael addition products are relatively stable, Schiff base reactions are physiologically slow and reversible [S74].

S6. Further Limitations of the Mak *et al.* Study (Ref. [79]).

Further potential limitations noted for the study reported in [79] were the possible impacts of therapeutic agents that both groups of patients were receiving. For example, inhibition of vasculature ROS generation in CHF group participants by ACE inhibitor therapies (n = 7 out of 8 patients), which conceivably could have underestimated the elevations observed in their plasma aldehydic LOP concentrations, if indeed they arose from *in vivo* lipid peroxidation. Notwithstanding, CHF-mediated upregulations of these secondary LOPs were perceived not to arise solely from the presence of coronary artery disease or its risk factors, the prevalence of which were found to be equivalent in the two groups compared.

S7. Blood Plasma Aldehyde Concentrations in Infants with Chronic Lung Disease

Ogihara *et al.* in 1999 [87] determined the blood plasma levels of aldehydic LOPs in premature infants with and without chronic lung disease (CLD), i.e. those of 3 long-chain *n*-alkanals, 4 *trans*-2-alkenalsand HNE only. In contrast to aldehydic LOP data available in [79], the age-matched non-CLD infants were found to have much higher levels of total *n*-alkanals (predominantly *n*-hexenal and -heptenal) than those of total α,β-unsaturated aldehydes (*i.e.* molar ratios of > 5), with HNE representing approximately 30% of the latter, more chemically-reactive classification. The relative abundances of these saturated and unsaturated aldehydes were in the order *n*-hexanal >> -heptanal >>> -pentanal, and HNE > *trans*-2-heptenal > -nonenal > -hexenal > -octenal, respectively. Notwithstanding, these researchers found that plasma levels of heptanal, 2-nonenal, and HNE were significantly elevated in CLD infants on the day of birth over those of non-CLD controls, but this was not the case at 4–6 days of age, and such results indicated that *in vivo* lipid peroxidation may be pathologically featured in neonatal CLD.

S8. DNA Adduct Formation with and Detoxification of *trans*-2-Hexenal

trans-2-Hexenal detoxification and DNA adduct generation in humans was tested in physiologically-based *in silico* models by Kiwamoto *et al.* [124]. In these experiments, *trans*-2-hexenal exposure gave rise to 0.039 adducts/108 nucleotides (nt) at its questionable quoted estimated mean dietary intake of only 40 μg/kg BW, and 0.18 adducts/108 nt at the corresponding 95th percentile of this estimated daily intake dataset (178 μg/kg BW, equivalent to 12.5 mg for a 70 kg human). However, these 108 nt-normalised levels were found to be up to 1,000-fold lower than the natural background DNA adduct values documented for disease-free humans (6.8–110 adducts/108 nt), and therefore it was concluded that the genotoxicity risk for *trans*-2-hexenal in humans was negligible. Notwithstanding, it is of much importance to note that the above estimated mean and 95th percentile values for the dietary intake of this aldehyde are only focused on a single *trans*-2-alkenal, and no allowance whatsoever has been made for the accumulative effects of a range of further aldehydes carried as toxicological hazards by fried food products, *i.e.* *trans*-2-octenal, -pentenal and -decenal, which represent the predominant

secondary *trans*-2-alkenal LOPs derived from the peroxidation of linoleoyl-, linolenoyl- and oleoylglycerols respectively [14].

S9. Cytotoxic and Mutagenic Potential of the Natural Product 2-Cyclohexene-1-one Evaluated against a Range of Dietary Aldehydes

Glaab *et al.* [S75] tested the cytotoxic and genotoxic potential of 2-cyclohexene-1-one, a natural product present in some tropical fruits and a soft drink contaminant, against those exhibited by the 2-alkenals *trans*-2-hexenal, *trans*-2-octenal, *trans*-2-nonenal, *trans,cis*-hexa-2,4-dienal, *trans,cis*-nona-2,6-dienal and cinnamaldehyde in V79 and Caco-2 cell lines. With the exception of *trans*-2-hexenal and cinnamaldehyde, all these α,β-unsaturated aldehydes displayed a much higher level of cytotoxicity than 2-cyclohexene-1-one following a 1.0 hr. incubation period. All aldehydes evaluated gave rise to higher levels of DNA damage than that exerted by 2-cyclohexene-1-one, apart from cinnamaldehyde. Intracellular glutathione levels also diminished following treatment with all these carbonyl compounds, as might be expected.

S10. Acrolein as an Inhaled or Ingested Toxin of Carcinogenic Potential: a Special Case for Consideration

In addition to its generation from the fragmentation of CHPDs arising from the peroxidation of ω-3 FAs (including α-linolenic acid, and fish oil-specific EPA and DHA) during high-temperature frying/cooking episodes, acrolein represents a special case for consideration since many recent intensive investigations have been focused on the adverse health effects of both dietary and/or environmental sources of it. This highly-reactive aldehyde is also liberated from the combustion of fuels, petroleum biodiesel, wood, paper and plastic sources, and is also a major toxin/carcinogen present in inhaled tobacco smoke [134].

One major, valuable consideration is the high level of diversity of tissues and organs in which acrolein has the ability to exert its damaging actions. In each case, there may be mechanisms which are either organ- or disease-explicit, in addition to more common non-specific ones, which may facilitate the design of targeted and non-targeted approaches, respectively, for the therapy and management of acrolein toxicity. However, in the case of orally-ingested exogenous acrolein, the GI system is the most important, primary site of acrolein attack and inducible damage.

Acrolein's ability to cause DNA damage (via chemical modification of its base moieties) and suppression of DNA repair pathways are features which engender its carcinogenicity. Notwithstanding, such actions remain a subject of considerable debate [S76]. When administered via the *i.p.* route, this reactive aldehyde, *i.e.* 75 and 150 nmol. per animal, elevated the frequency of DNA damage adducts, but not the incidence of tumours in both male and female B6C3F1 mice, an observation indicating that such DNA modifications did not correlate with cancer development [S77]. However, it should be noted that such doses are extremely small, and assuming a mean animal mass of 15 g, these values equate to only 1.87 and 3.75 μmol./kg, which is extremely low when compared to typical dietary aldehydic intake assaults, which in one study was reported as 5 mg/kg for the total unsaturated aldehyde intake value in humans [19], of which the proportion of dietary acrolein will vary according to variations in dietary patterns and frying oil use, including the oils and frying methods used, etc. For example, populations with high intakes of marine oils, which contain high contents of acrolein-generating ω-3 FAs, particularly those exposed to high temperature frying episodes which substantially promote their peroxidation. Similarly, Western diets using relatively high levels of soybean, canola or rapeseed oils as frying media, with low but nevertheless significant ω-3 FA contents (*ca.* 7–12% (w/w)), will also produce much higher levels of this potential aldehydic carcinogen.

An epidemiological study performed in 2002 [156] explored relationships between lung cancer risk in Chinese women and the use of locally-manufactured rapeseed and linseed oils for stir-frying practices, and these researchers found that for women who frequently performed stir-fry cooking methods, there was a significantly enhanced risk of lung cancer, especially when using rapeseed oil. As might be expected, this risk increased with the total number of years that they had been exposed to

cooking oil fumes. Moreover, some evidence for a contribution towards this risk value from exposure to linseed oil cooking fumes was also obtained. Incidences of eye and throat irritations arising from the employment of these oils for frying purposes also bestowed a significantly increased lung cancer risk. This investigation is of much interest and value, since both these oils contain relatively high levels of linolenoylglycerols, linseed oil exceedingly so: *ca.* 8% (w/w) for rapeseed, and as much as *ca.* 55% (w/w) for linseed oils. Both acrolein and MDA are liberated from the degradation of hydroperoxides generated from this and other ω-3 PUFAs, and do not arise from the fragmentation of ω-6 PUFAs such as vegetable oil-prevalent linoleoylglycerols, although acrolein can also potentially be formed from free glycerol oxidation. Therefore, it is conceivable that these specific aldehydes play a major contributory role towards the development of lung cancer (notably adenocarcinoma) in Chinese women exposed to wok cooking fumes, especially acrolein.

Acrolein also acts as a respiratory and pulmonary toxin, and hence has been implicated in a range of pulmonary conditions, *e.g.* pulmonary oedema, amplification of bronchial responsiveness [S78], and also chronic obstructive pulmonary disease (COPD) [28], the latter inducible and exacerbated by the inhalation of acrolein-and crotonaldehyde-rich tobacco smoke (estimated contents of these aldehydes in a daily 25-cigarette allocation of them are 0.62-3.5 [S79] and 1.8-5.7 mg [137] respectively).

Feroe *et al.* [S80] also investigated the influence of ingested acrolein intake on diabetes and insulin resistance, and for this purpose they explored associations between its urinary creatinine-normalised metabolites, specifically (N-acetyl-S-(3-hydroxypropyl)-L-cysteine and N-acetyl-S-(carboxyethyl)-L-cysteine, and their molar sum, in a very large number of participants with these disorders, who were recruited to the 2005-2006 National Health and Nutrition Examination Survey (NAHANES). This study demonstrated that both these conditions were positively associated with the excretion of both urinary metabolites, along with their summed total values. Some evidence for a dose-response relationship was also obtained.

S11. Dietary Sources, Inhalation/Ingestion, Cytotoxicity and Genotoxicity of *t,t*-DDE

Available reports [S81-S83] have estimated that the combined daily human intake of a total of 12 of these flavour enhancers was *ca.* 40 μg in Europe and *ca.* 120 μg in the USA, and that for *trans,trans*-deca-2,4-dienal alone was *ca.* 20 μg in Europe and *ca.* 70 μg in the USA (the estimated value for all the remaining flavouring agents in this group was stated as being within the 0.007–24 μg/day range). However, such estimates are unfortunately solely and loosely based on the intake of these agents, predominantly *t,t*-DDE, as added flavour-enhancing agents. Indeed, our estimate of 3.8 mg of total *trans,trans*-2,4-alkadienals (predominantly *t,t*-DDE) alone in a single 154 g portion of fried potato chips for human consumption is substantially greater than the above flavouring agent-based one, and therefore conceivable public health threats presented by its non-flavouring dietary intake are certainly worthy of consideration. Our mean±SEM estimate of 157 ± 43 μmol./kg for total *trans,trans*-alka-2,4-deienals [14] is not dissimilar to the maximal deep-frying value of *ca.* 65 $\mu mol.kg^{-1}$ (10–11 ppm) for *trans,trans*-deca-2,4-dienal alone in French fries estimated by Boskou *et al.* [S84]. However, our higher determined mean value [14] will also include contributions from all other *trans,trans*-alka-2,4-dienals present.

t,t-DDE, which arises from the thermolytic β-scission fragmentation of linoleoylglycerol hydroperoxides, exerts a high level of cellular toxicity in the liver and kidney [S85], along with its ability to induce the proliferation of GI epithelial cells [S86]. In view of its high reactivity towards DNA base adducts [S87], currently there are numerous concerns regarding strong linkages between its inhalation and/or ingestion and cancer development in humans. Since this class of aldehydes (*trans,trans*-2,4-alkadienals) are readily generated from the thermally-induced peroxidation of PUFAs within COs and foods, and also have an extensive incidence, albeit at much lower contents, as flavouring agents in food products [S47], *t,t*-DDE is considered by the National Cancer Institute (NCI) and National Toxicology Program (NTP) at the National Institutes of Environmental Health Sciences (NIEHS) as a major priority. Indeed, strong supporting evidence for a very disturbing increase in the incidence of lung adenocarcinoma in non-smoking women based in China, Hong Kong, Taiwan and Singapore has been

provided by a series of epidemiological studies [S88–S91], and this rise has been ascribed to their rather excessive exposure to cooking oil fumes [S88,S90–S92]. Although these fumes are, of course, highly complex, multicomponent human toxin admixtures [S93], and their compositions vary with their frying oil sources and frying conditions such as temperature, aldehydic LOPs such as *t,t*-DDE represent major constituents. Indeed, *t,t*-DDE is one of the most predominant α,β-unsaturated aldehydes detectable therein, along with acrolein [188].

The toxicological effects exerted by this reactive aldehyde towards a non-cancerous human bronchial epithelial cells (BEAS-2B) have been previously investigated [S86], and this study reported a significant boost to their ROS production capacity, and also changes to their GSH/glutathione disulphide ([GSH]:[GSSG]) balance, the latter of which is presumably ROS-mediated; significant decreases in [GSH]:[GSSG] ratios were observed when this cell line was exposed to 1–5 μM *t,t*-DDE short-term (48 hr.), and likewise only 0.10-1.00 μM long-term (up to 30 days). Furthermore, an increased cellular proliferation was observed when these cell lines were incubated with 1.0 μmol/L *t,t*-DDE for a 45 day period. The expression and release of the pro-inflammatory cytokines TNFα and interleukin 1B (IL-1B) was also observed. Since these cytokines are associated with the augmentation of tumours, *t,t*-DDE (and likewise other members of this class of aldehydes), may promote the development of lung epithelial cell tumours. Furthermore, *t,t*-DDE was found to give rise to oxidative stress, and exerted genotoxic actions towards human lung carcinoma A549 cells [S94].

References

S1 Neff, W. E.; Byrdwell W. C. Characterization of model triacylglycerol (triolein, trilinolein and trilinolenin) autoxidation products via high-performance liquid chromatography coupled with atmospheric pressure chemical ionization mass spectrometry. *J. Chromatogr. A*. 1998, 818, 169–186.

S2 Markaverich, B. M.; Crowley, J. R.; Alejandro, M. A.; Shoulars, K.; Casajuna, N.; Mani, S.; Reyna, A.; Sharp, J. Leukotoxins diols from ground corncob bedding disrupt estrus cyclicity in rats and stimulate MCF-7 breast cancer cell proliferation. *Environ. Health Persp.* 2005, 113, 1698–1704. doi: 10.1289/ehp.8231.

S3 Thompson, D. A.; Hammock, B. D. Dihydroxyoctadecamonoenoate esters inhibit the neutrophil respiratory burst. *J. Bioscience* **2007**, 32, 279–291.

S4 Liu, Y.; Cheng, Y.; Li, J.; Wang, Y.; Liu, Y. Epoxy stearic acid, an oxidative product derived from oleic acid, induces cytotoxicity, oxidative stress, and apoptosis in HepG2 cells. *J. Agricul. Food Chem.* **2018,** 66, 5237–5246. DOI: 10.1021/acs.jafc.8b01954

S5 Deol, P.; Fahrmann, J.; Yang, J.; Evans, J. R.; Rizo, A.; Grapov, D.; Salemi, M.; Wanichthanarak, K.; Fiehn, O.; Phinney, B.; Hammock, B. D.; Sladek, F. M. Omega-6 and omega-3 oxylipins are implicated in soybean oil-induced obesity in mice. *Sci. Rep.* **2017**, 7. DOI: 10.1038/s41598-017-12624-9

S6 Wilson, R.; Fernie, C. E.; Scrimgeour, C. M.; Lyall, K.; Smyth, L.; Riemersma, R. A. Dietary epoxy fatty acids are absorbed in healthy women. *Eur. J. Clin. Invest.* **2002**, 32(2), 79–83.

S7 Greene, J. F.; Newman, J. W.; Williamson, K. C.; Hammock, B. D. Toxicity of epoxy fatty acids and related compounds to cells expressing human soluble epoxide hydrolase. *Chem. Res. Toxicol.* **2000**, 13, 217–226. DOI: 10.1021/tx990162c

S8 Wagner, K.; Vito, S.; Inceoglu, B.; Hammock, B. D. The role of long chain fatty acids and their epoxide metabolites in nociceptive signalling. *Prostaglandins Other Lipid Mediat.* **2014**, 0, 2–12.

S9 Alfaia, C.P.M.; Alves, S.P.; Lopes, A.F.; Fernandes, M.F.E.; Costa, A. S. H.; Fontes C. M. G. A.; Castro, M. L; Bessa, R. J.; Prates, J. A. Effect of cooking methods on fatty acids, conjugated isomers of linoleic acid and nutritional quality of beef intramuscular fat. *Meat Science*. **2010**, 84, 769–777.

S10 Broncano, J.M.; Petrón, M.J.; Parra, V.; Timón, M.L. Effect of different cooking methods on lipid oxidation and formation of free cholesterol oxidation products (COPs) in Latissimus dorsi muscle of Iberian pigs. *Meat Science*. **2009**, 83, 431–437.

S11 Ana, Lúcia F.; Pereira; Virgínia Kelly G. Abreu. Lipid Peroxidation in Meat and Meat Products DOI: http://dx.doi.org/10.5772/intechopen.81533

S12 Guyon, C.; Meynier, A.; Lamballerie, M. Protein and lipid oxidation in meat: A review with emphasis on high pressure treatments. *Trends in Food Science and Technology*. **2016**, 50, 131–143.

S13 Martino, G.; Grotta, L.; Ponzielli, V. Influence of dehydrated Medicago sativa on quality characteristics of Marchigiana beef. *Animal Review*. **2014**c, 1:37–44.

S14 Min, B.; Ahn, D.U. Mechanism of lipid peroxidation in meat and meat products. *Food Science and Biotechnology*. 2005;14:152–163.
S15 Delles, R.M.; Xiong, Y.L.; True, A.D.; Ao, T.; Dawson, K.A. Dietary antioxidant supplementation enhances lipid and protein oxidative stability of chicken broiler meat through promotion of antioxidant enzyme activity. *Poultry Science*, **2014**, 93, 1561–1570.
S16 Ajuwon, O.R.; Idowu, O.M.O. Vitamin C attenuates copper - induced oxidative damage in broiler chickens. *Af. J. Biotechnol.*, **2010**, 9, 7525–7530.
S17 Wu, B., Cui, H., Peng, X. *et al*. Investigation of the serum oxidative stress in broilers fed on diets supplemented with nickel chloride. *Health*, **2013**, 5, 454.
S18 Addis, P.B. Occurrence of lipid oxidation products in foods. *Food Chem*. Toxicol. **1986**, 24, 1021–1030.
S19 Jackson, V.; Penumetcha, M. Dietary oxidised lipids, health consequences and novel food technologies that thwart food lipid oxidation: an update. *Intern. J. Food Sci. Technol*. **2019**, 54, 1981–1988.
S20 Dantas, N. M.; Sampaio, G. R.; Ferreira, F. S.; Labre, T. S.; Ferrazda, E. A.; Torres, S.; Saldanha, T. Cholesterol oxidation in fish and fish products. *J. Food Sci.* **2015**, 80, R2627–R2639.
S21 Giulia, Secci.; Giuliana, Parisi. From farm to fork: lipid oxidation in fish products. A review, *Ital. J. Animal Sci.*, **2016**, 15, 124–136, DOI: 10.1080/1828051X.2015.1128687
S22 Hematyar, N.; Rustad, T.; Sampels, S.; Kastrup Dalsgaard, T. Relationship between lipid and protein oxidation in fish. Aquac Res. **2019**, 50: 1393–1403. https://doi.org/10.1111/are.14012
S23 Maqsood, S.; Benjakul, S. Effect of bleeding on lipid oxidation and quality changes of Asian seabass (Lates calcarifer) muscle during iced storage. *Food Chem*. **2011**, 124, 459–467.
S24 Karlsdottir, M.G.; Sveinsdottir, K.; Kristinsson, H.G.; Villot, D.; Craft, B.D.; Arason, S. Effects of temperature during frozen storage on lipid deterioration of saithe (Pollachius virens) and hoki (Macruronus novaezelandiae) muscles. *Food Chem*. **2014**a, 156:234–242.
S25 Stephen, N. M.; Jeya Shakila, R.; Jeyasekaran, G.; Sukumar, D. Effect of different types of heat processing on chemical changes in tuna. *Food Sci. Technol.* **2010**, 47(2), 174–181. DOI: 10.1007/s13197-010-0024-2.
S26 Faulkner, H., O'Callaghan, T.F., McAuliffe, S.; Hennessy, D.; Stanton, C.; O'Sullivan, M. O.; Kerry, J. P.; Kilcawley, K. N. Effect of different forage types on the volatile and sensory properties of bovine milk. *Journal of Dairy Science*, **2018**, 101, 1034–1047.
S27 Welter, K.C.; Martins, C.M. de M.R.; Palma, A.S.V. de.; Martins, M. M.; dos Reis, B.R.; Bárbara Laís Unglaube Schmidt, B. L. U.; Netto, A. S. Canola oil in lactating dairy cow diets reduces milk saturated fatty acids and improves its omega-3 and oleic fatty acid content. *PLoS ONE*, **2016**, 11, e0151876.
S28 Ullah, R.; Nadeem, M.; Imran, M. Omega-3 fatty acids and oxidative stability of ice cream supplemented with olein fraction of chia (*Salvia hispanica* L.) oil. *Lipids in Health and Disease*, **2017**, 16, 1–8.
S29 Cappozzo, J.C.; Koutchma, T.; Barnes, G. Chemical characterization of milk after treatment with thermal (HTST and UHT) and nonthermal (turbulent flow ultraviolet) processing technologies. *Journal of Dairy Science*, **2015**, 98, 5068–5079.
S30 Pereda, J.; Ferragut, V.; Quevedo, J.M.; Guamis, B.; Trujillo, A.J. Effects of ultra-high-pressure homogenization treatment on the lipolysis and lipid oxidation of milk during refrigerated storage. *J Agric Food Chem.* **2008**, 27, 7125–7130. doi: 10.1021/jf800972m. Epub 2008 Jul 12.
S31 Collin, Sonia.; Osman, M.; Delcambre, S.; Elzayat, AI.; Dufour, J.P. Investigation of Volatile Flavor Compounds in Fresh and Ripened Domiati Cheeses. In: *J. Agricul. Food Chem* **1993**, 41, 1659–1663. http://hdl.handle.net/2078.1/49471
S32 Yodkaew, P.; Chindapan, N; Devahastin, S. Influences of superheated steam roasting and water activity control as oxidation mitigation methods on physicochemical properties, lipid oxidation, and free fatty acids compositions of roasted rice. *J. Food Sci.*, **2017**, 82, 69–79.
S33 Barden, L.; Vollmer, D.; Johnson, D.; Decker, E. Impact of iron, chelators, and free fatty acids on lipid oxidation in low - moisture crackers. *J. Agricul. Food Chem*, **2015**, 63, 1812–1818.
S34 PerezHerrera, A., Rangel-Zuñiga, O.A.; Delgado-Lista, J. Marin, C.; Perez-Martinez, P.; Tasset, I.; Tunez, I.; Quintana-Navarro, G-M.; Lopez-Segura, F.; Luque de Castro, M. D. *et. al.* The antioxidants in oils heated at frying temperature, whether natural or added, could protect against postprandial oxidative stress in obese people. *Food Chemistry*, **2013**, 138, 2250–2259.
S35 Major, R. T.; Marchini, P.; Sproston, T. Isolation from Gingko balboa of an inhibitor of fungus growth. *J Biol. Chem.* **1960**, 235, 3298–3299.

S36 Marnett, L. J.; Hurd, H. K.; Hollstein, M. C.; Levin, D. E.; Esterbauer, H.; Ames, B. N. Naturally occurring carbonyl compounds are mutagens in Salmonella-tester-strain TA 104. *Mutat. Res.* **1985**, 148, 25–34.

S37 Eder, E.; Deininger, C.; Neudecker, T.; Deininger, D. Mutagenicity of β-alkyl substituted acrolein congeners in the Salmonella typhimurium strain TA 100 and genotoxicity testing in the SOS-chromotest. *Environ. Mol. Mutag.* **1992**, 19, 337–345.

S38 Canonero, R.; Martelli, A.; Marinari, U. M.; Brambilla, G. Mutation induction in the Chinese hamster lung V79 cells by five alk-2-enals produced by lipid peroxidation. *Mutat. Res.* **1990**, 244, 153–156.

S39 Eder, E.; Scheckenbach, S.; Deininger, C.; Hoffman, C. The possible role of α,β-unsaturated carbonyl compounds in mutagenesis and carcinogenesis. *Toxicol. Lett.* **1993**, 67, 87–103.

S40 Eisenbrand, G.; Schuhmacher, J.; Gölzer, P: The influence of glutathione and detoxifying enzymes on DNA damage induced by 2-alkenals in primary rat hepatocytes and human lymphoblastoid cells. *Chem. Res. Toxicol.* **1995**, 8, 40–46.

S41 Dittberner, U.; Eisenbrand, G.; Zankl, H. Genotoxic effects of the α,β-unsaturated aldehydes 2-trans-butenal, 2-trans-hexenal and 2-trans,6-cis-nonadienal. *Mutat. Res.* **1995**, 335, 259–265.

S42 Kallio, H.; Linko, R.R. Volatile monocarbonyl compounds of arctic bramble (Rubus arcticus L.) at various stages of ripeness. *Zeitschrift für Lebensmittel-Untersuchung und -Forschung*, **1973**, 153, 23–30.

S43 Hayase, F.; Chung, T-Y.; Kato H. Changes of volatile components of tomato fruits during ripening. *Food Chemistry*, **1984**, 14, 113–124.

S44 Serrano, M.; Gallego, M.; Silva, M. Quantitative analysis of aldehydes in canned vegetables using static headspace–gas chromatography–mass spectrometry. *J. Chromatog. A*, 2017, 1524, 21–28. https://doi.org/10.1016/j.chroma.2017.09.067.

S45 Greenhoff. K.; Wheeler. R.E. Analysis of beer carbonyls at the part per billion level by combined liquid chromatography and high pressure liquid chromatography. *J. Instit. Brewing*, **1981**, 86, 35–41.

S46 Subden, R.E.; Krizus, A.; Akhtar, M. Mutagen content of Canadian apple eau-de-vie. *Canad. Instit. Food Sci. Technol. J.* **1986**, 19,134–136.

S47 Williams, G. WHO Food Additives Series: 52. Aliphatic, alicyclic, linear alpha,beta-unstaurated di- and trienals and related alcohols, acids and esters. Prepared by the Sixty-first meeting of the Joint FAO/WHO Expert Committee on Food Additives (JECFA). World Health Organization, Geneva, 2004. International program on chemical safety (IPCS) INCHEM Home. http://www.inchem.org/documents/jecfa/jecmono/v52je14.htm

S48 Frankel, E. N.; Neff, W.E.; Brooks, D. D.; Fujimoto, K. Fluorescence formation from the interaction of DNA with lipid oxidation degradation products. *Biochim. Biophys. Acta* **1987**, 919, 239–244.

S49 Stofberg, J.; Grundschober, F. Consumption ratio and food predominance of flavoring materials. *Perfumer Flavorist*. **1987**, 12, 27.

S50 Maarse, H.; Visscher, C. A.; Willemsens, L. C.; Boelens, M. H. *Volatile Components In Food—Qualitative And Quantitative Data*. 1999, Zeist: Centraal Instituut Voor Voedingsonderzioek TNO.

S51 Hoydonckx, H. E.; Van Rhijn, W. M.; Van Rhijn, W.; De Vos, D. E.; Jacobs, P. A. Furfural and Derivatives. In *Ullmann's Encyclopedia of Industrial Chemistry*. Wiley-VCH: Weinheim, Germany, 2007. doi:10.1002/14356007.a12_119.pub2

S52 Kroes, R. WHO Food Additives Series: 42. Furfural. Prepared by the Fifty-first meeting of the Joint FAO/WHO Expert Committee on Food Additives (JECFA). World Health Organization, Geneva, **1999**. International program on chemical safety (IPCS) INCHEM Home.

S53 Weerawatanakorn, M.; Wu, J-C.; Pan M-H.; Ho, C-T. Reactivity and stability of selected flavor compounds. *J. Food Drug Anal.* **2015**, 23, 176–190. ISSN 1021–9498. https://doi.org/10.1016/j.jfda.2015.02.001. (http://www.sciencedirect.com/science/article/pii/S1021949815000277)

S54 Robles, E. Thermal decomposition products of cellophane. McClellan Air Force Base, CA, US Air Force Environmental Health Library (Report AD-752515), **1968**.

S55 Zitting, A.; Heinonen, T. Decrease of reduced glutathione in isolated rat hepatocytes caused by acrolein, acrylonitrile, and the thermal degradation products of styrene copolymers. *Toxicology* **1980,** 17, 333–341.

S56 Risk assessment of aldehydes. Scientific Report, National Institute of Food and Drug Safety Evaluation, Ministry of Food and Drug Safety., **2016,** Korea.

S57 Lachenmeier, D. W.; Kanteres, F.; Rehm, J. Carcinogenicity of acetaldehyde in alcoholic beverages: risk assessment outside ethanol metabolism. *Addiction* **2009**, 104, 533–550. doi:10.1111/j.1360-0443.2009.02516.x
S58 Burdock, G. A. *Fenaroli's Handbook of Flavour Ingredients*, 5th edn. **2004**. *Boca Raton*, FL: CRC Press.
S59 Joint FAO/WHO Expert Committee on Food Additives (JECFA). Saturated aliphatic acyclic linear primary alcohols, aldehydes, and acids. In: WHO Food Additives Series 40. Safety Evaluation of Certain Food Additives and Contaminants. Geneva, Switzerland: World Health Organization. **1998**, 148–188.
S60 Rehm, J.; Room, R.; Taylor, B. Method for moderation: measuring lifetime risk of alcohol-attributable mortality as a basis for drinking guidelines. *Int. J. Methods Psychiatr. Res.* **2008**, 17, 141–151.
S61 Powers, S. J.; Mottram, D. S.; Curtis, A.; Halford, N. G. Acrylamide concentrations in potato crisps in Europe from 2002 to 2011. *Food Addit. Contam. Part A-Chem. Anal. Control Expos. Risk Assess* **2013**, 30, 1493–1500.
S62 Zelinková, Z.; Doležal, M.; Velíšek, J. 3-Chloropropane-1,2-diol fatty acid esters in potato products. *Czech. J. Food Sci.* **2009**, 27, S421–424.
S63 Ascherio, A.; Stampfer, M. J.; Willett, W. C. *Trans*-fatty acid intake and risk of myocardial infarction. *Circulation* **1994**, 101, 94–101.
S64 Mozaffarian, D.; Aro, A.;.Willett, W. C. Health effects of *trans*-fatty acids: experimental and observational evidence. *Eur. J. Clin. Nutr.* **2009**, 63, S5–S21; doi:10.1038/sj.ejcn.1602973
S65 Kris-Etherton, P. M.; Lefevre, M.; Mensink, R. P.; Petersen, B.; Fleming, J.; Flickinger, B. D. Trans fatty acid intakes and food sources in the U.S. population: NHANES 1999–2002. *Lipids* **2012**, 47, 931–940. Epub 2012 Aug 18.
S66 Zhong, H.; Yin, H. Role of lipid peroxidation derived 4-hydroxynonenal (4-HNE) in cancer: focusing on mitochondria. *Redox Biol*. **2015**, 4, 193–199. doi:10.1016/j.redox.2014.12.011
S67 Mattson, M. P. Roles of the lipid peroxidation product 4-hydroxynonenal in obesity, the metabolic syndrome, and associated vascular and neurodegenerative disorders. *Exp. Gerontol*. **2009**, 44, 625–633. doi:10.1016/j.exger.
S68 Eckl, P. Genotoxicity of HNE. *Molec. Asp. Med.* **2003**, 24, 161–165. DOI: 10.1016/S0098-2997(03)00010-4
S69 Eckl, P. M.; Ortner, A.; Esterbauer, H. Genotoxic properties of 4-hydroxyalkenals and analogous aldehydes. *Mutat. Res.* **1993**, 290, 183–192.
S70 Usatyuk, P. V.; Parinandi, N. L.; Natarajan, V. Redox Regulation of 4-hydroxy-2-nonenal-mediated endothelial barrier dysfunction by focal adhesion, adherens, and tight junction proteins. *J. Biol. Chem.* **2006**, 281, 35554–35566. DOI 10.1074/jbc.M607305200
S71 Zheng, R.; Dragomir, A. C.; Mishin, V.; Richardson, J. R.; Heck, D. E.; Laskin, D. L.; Laskin, J. D. Differential metabolism of 4-hydroxynonenal in liver, lung and brain of mice and rats. *Toxicol. Appl. Pharmacol.* **2014,** 279, 43–52. doi:10.1016/j.taap.2014.04.026
S72 Siems, W.; Grune, T. Intracellular metabolism of 4-hydroxynonenal *Molec. Asp. Med.* **2003**, 24, 167–175.
S73 Schauer, J. Basic aspects of the biochemical reactivity of 4-hydroxynonenal. *Molec. Asp. Med.* **2003**, 24, 149–159.
S74 Castro, J. P.; Jung, T.; Grune, T.; Siems, W. 4-Hydroxynonenal (HNE) modified proteins in metabolic diseases. *Free Rad. Biol. Med.* **2017**, 111, 309–315.
S75 Glaab, V.; Collins, A. R.; Eisenbrand, G.; Janzowski, C. DNA-damaging potential and glutathione depletion of 2-cyclohexene-1-one in mammalian cells, compared to food relevant 2-alkenals. *Mutat. Res. – Genet Toxicol. Environ. Mutagen.* **2001**, 497, 185–197.
S76 Abraham, K.; Andres, S.; Palavinskas, R.; Berg, K.; Appel, K. E.; Lampen, A. Toxicology and risk assessment of acrolein in food. *Mol. Nutr. Food Res.* **2011**, 55, 1277–1290.
S77 Von Tungeln, L. S.; Yi, P.; Bucci, T. J.; Samokyszyn,V. M.; Chou, M. W.; Kadlubar, F. F.; Fu, P. P. Tumorigenicity of chloral hydrate, trichloroacetic acid, trichloroethanol, malondialdehyde, 4-hydroxy-2-nonenal, crotonaldehyde, and acrolein in the B6C3F(1) neonatal mouse. *Cancer Lett.* **2002**, 185, 13–19.
S78 Agency for Toxic Substances and Disease Registry, Toxicological profile for acrolein. US Department of Health and Human Services, Public Health Service. Atlanta, GA, USA **2007.**
S79 Esterbauer, H.; Schaur, R. J.; Zollner, H. Acrolein: Chemistry and biochemistry of 4-hydroxynonenal, malonaldehyde and related aldehydes. *Free Rad. Biol Med.* 1991, **11**, 81–128. [PubMed: 1937131].
S80 Feroe, A, G.; Attanasio, R,; Scinicariello, F. Acrolein metabolites, diabetes and insulin resistance. *Environment. Res.* **2016**, 148, 1–6.
S81 National Academy of Sciences *Evaluating the Safety of Food Chemicals*. NAS: Washington DC, USA **1982**.
S82 International Organization of the Flavor Industry. *European inquiry on volume use*. Unpublished report, Submitted to WHO by the Flavor and Extract Manufacturers Association of the United States, Washington DC, USA **1995**.

S83 Lucas, C. D.; Putnam, J. M.; Hallagan, J. B. *Flavor and Extract Manufacturers Association of the United States 1995 Poundage and Technical Effects Update Survey*, Flavor and Extract Manufacturers Association of the United States: Washington, DC, USA, **1999**.

S84 Boskou, G.; Salta, F.N.; Chiou, A.; Troullidou, E.; Andrikopoulos, N.K. Content of trans,trans-2,4-decadienal in deep-fried and pan-fried potatoes. *Eur. J. Lipid Sci. Technol.* **2006**, 108, 109–115.

S85 Hageman, G.; Verhagen, H.; Schutte, B.; Kleinjans, J. Biological effects of short-term feeding to rats of repeatedly used deep-frying fats in relation to fat mutagen content. *Food Chem. Toxicol.* **1991**, 29, 689–698.

S86 Chang, L. W.; Lo, W-S.; Lin, P. *Trans,trans*-2,4-decadienal, a product found in cooking oil fumes, induces cell proliferation and cytokine production due to reactive oxygen species in human bronchial epithelial cells. *Toxicol. Sci.* **2005**, 87, 337–343. doi:10.1093/toxsci/kfi258.

S87 Carvalho, V. M.; Asahara, F.; Di MascioIvan, P.; Arruda de Campos, P. Cadet, J.; Medeiros, M. H. G.. 1, N6-etheno-2′-deoxyadenosine adducts from trans,trans-2,4-decadienal and trans-2-octenal. *Adv. Exp. Med. Biol.* **2001**, 500, 229–232.

S88 Ko, Y. C.; Cheng, L. S-C.; Lee, C-H.; Huang, J-J.; Huang, M-S.; Kao, E-L.; Wang, H-Z.; Lin, H-J. Chinese food cooking and lung cancer in women non-smokers. *Am. J. Epidemiol.* **2000**, 151, 140–147.

S89 Tan, Y. K.; Wee, T. C.; Koh, W. P.; Wang, Y. T.; Eng, P.; Tan, W. C.; Seow. A. Survival among Chinese women with lung cancer in Singapore: A comparison by stage, histology and smoking status. *Lung Cancer* **2003**, 40, 237–246.

S90 Zhong, L.; Goldberg, M. S.; Parent, M. E.; Hanley, J. A. Risk of developing lung cancer in relation to exposure to fumes from Chinese-style cooking. *Scand. J. Work Environ. Health* **1999**, 25, 309–316.

S91 I. T. S. Yu; Chiu, Y-l; Au, J. S. K.; Wong, T-w.; Tang, J-l. Dose-response relationship between cooking fumes exposures and lung cancer among Chinese non-smoking women. *Cancer Res.* **2006**, 66(9), 4961–4967. DOI**:** 10.1158/0008-5472.CAN-05-2932

S92 Zhong, L.; Goldberg, M. S.; Gao, Y. T.; Jin, F. Lung cancer and indoor air pollution arising from Chinese-style cooking among non-smoking women living in Shanghai, China. *Epidemiology* **1999**, 10, 488–494.

S93 Shields, P. G.; Xu, G.X.; Blot, W.T.; Fraumeni, J.F.; Trivers, G.E.; Pellizzari, E.D.; Qu, Y.H.; Gao, Y.T.; Harris, C.C. Mutagens from heated Chinese and U.S. cooking **o**ils**.** *JNCI J Natl Cancer Inst.* **1995**, 87(11), 836–841. doi: 10.1093/jnci/87.11.836

S94 Wu, S. C.; Yen, G. C. Effects of cooking oil fumes on the genotoxicity and oxidative stress in human lung carcinoma (A-549) cells. *Toxicol. In Vitro* **2004**, 18, 571–580.
